# A Novel Adaptive Superb Fairy-Wren (*Malurus cyaneus*) Optimization Algorithm for Solving Numerical Optimization Problems

**DOI:** 10.3390/biomimetics10080496

**Published:** 2025-07-27

**Authors:** Tianzuo Yuan, Huanzun Zhang, Jie Jin, Zhebo Chen, Shanshan Cai

**Affiliations:** 1 Faculty of Health Sciences, University of Macau, Macau 999078, China; cc31642@umac.mo; 2 Stony Brook Institute, Anhui University, Hefei 230039, China; r32214025@stu.ahu.edu.cn; 3 Taizhou Institute of Zhejiang University, Taizhou 318000, China; chenzhebo@tzizju.cn; 4 Division of Biomedical and Life Sciences, Faculty of Health and Medicine, Lancaster University, Lancaster LA1 4YG, UK

**Keywords:** superb fairy-wren optimization algorithm, adaptive switching framework, covariance matrix, numerical optimization

## Abstract

Superb Fairy-wren Optimization Algorithm (SFOA) is an animal-based meta-heuristic algorithm derived from Fairy-wren’s behavior of growing, feeding, and avoiding natural enemies. The SFOA has some shortcomings when facing complex environments. Its switching mechanism is not enough to adapt to complex optimization problems, and it faces a weakening of population diversity in the late stage of optimization, leading to a higher possibility of falling into local optima. In addition, its global search ability needs to be improved. To address the above deficiencies, this paper proposes an Adaptive Superb Fairy-wren Optimization Algorithm (ASFOA). To assess the ability of the proposed ASFOA, three groups of experiments are conducted in this paper. Firstly, the effectiveness of the proposed improved strategies is checked on the CEC2018 test set. Second, the ASFOA is compared with eight classical/highly cited/newly proposed metaheuristics on the CEC2018 test set, in which the ASFOA performed the best overall, with average rankings of 1.621, 1.138, 1.483, and 1.966 in the four-dimensional cases, respectively. Then the convergence and robustness of ASFOA is verified on the CEC2022 test set. The experimental results indicate that the proposed ASFOA is a competitive metaheuristic algorithm variant with excellent performance in terms of convergence and distribution of solutions. In addition, we further validate the ability of ASFOA to solve real optimization problems. The average ranking of the proposed ASFOA on 10 engineering constrained optimization problems is 1.500. In summary, ASFOA is a promising variant of metaheuristic algorithms.

## 1. Introduction

There are many complex problems in today’s society, most of which can be transformed into optimization problems. Optimization problems usually contain three factors: objective function, constraints, and decision variables [1]. With the advancement of science and technology, real-world optimization problems are becoming more and more complex, moving towards high-dimensional, multimodal, nonlinear, and multi-constrained directions [2]. Traditional optimization algorithms, such as gradient descent [3], Newton’s method [4], and quasi-Newton method [5], rely on accurate mathematical analytical forms and constraint specification [6]. When addressing complex real-world problems characterized by non-convex, discontinuous, or high-dimensional solution spaces, traditional optimization methods are no longer applicable [7]. Meta-heuristic algorithms have been proposed to better solve these problems with complex structures. Meta-heuristic optimization algorithms are used to find approximate solutions to difficult problems and are often defined as problem-independent algorithms [8]. Due to their high efficiency and high dimensionality, good performance, and wide range of applications in solving mathematical and engineering problems, metaheuristic optimization algorithms have been widely used to solve discontinuous and highly nonlinear problems. Over the past decade, metaheuristic algorithms have gradually replaced traditional algorithms and continue to influence the industry. The metaheuristic optimization algorithm consists of two phases, global search (exploration) and local search (exploitation). In the exploration phase, the algorithm looks at the entire solution space and explores new search regions to maintain diversity. In the optimization phase, the search is mainly performed in the neighborhood of the current solution to find better values. As an excellent class of stochastic algorithms, they are applied in many areas such as UAV mission planning problems [9,10,11], multilevel threshold image segmentation problems [12,13,14], feature selection problems [15,16,17], hyperparameter optimization problems [18,19], wireless sensor network coverage optimization problem [20,21], and energy scheduling optimization problems [22,23].

**Figure 1:**
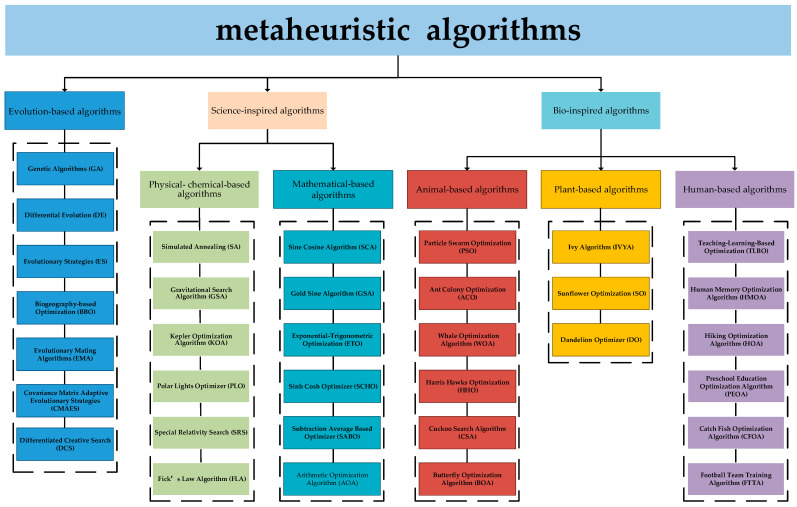
The classification of meta-heuristic algorithms.

Meta-heuristic algorithms, depending on their source of inspiration, can be divided into three broad categories: evolution-based algorithms (EAs), science-inspired algorithms (SIAs), and bio-inspired algorithms (BIAs). The specific classification structure is shown in Figure 1. EAs are a class of metaheuristic algorithms inspired by natural evolutionary mechanisms, of which the best known are Genetic Algorithm (GA) [24] and Differential Evolution (DE) [25]. GA originates from natural selection and genetics mechanisms to solve optimization problems through selection, crossover, and mutation operations. DE utilizes the difference vectors between individuals to guide the search. Other EAs include Evolutionary Strategies (ES) [26], Biogeography-based Optimization (BBO) [27], Evolutionary Mating Algorithm (EMA) [28], Covariance Matrix Adaptive Evolutionary Strategies (CMAES) [29], and Differentiated Creative Search (DCS) [30]. SIAs draw inspiration primarily from physical principles, chemical laws, and mathematical theorems. Thereby, SIAs can be further categorized into physical–chemical-based algorithms and mathematical-based algorithms. Simulated Annealing (SA) [31] and Gravitational Search Algorithm (GSA) [32] are the best-known physical–chemical-based algorithms. Others include, but are not limited to, Kepler Optimization Algorithm (KOA) [33], Polar Lights Optimizer (PLO) [34], Special Relativity Search (SRS) [35], and Fick’s Law Algorithm (FLA) [36]. From the time when Sine Cosine Algorithm (SCA) [37] was proposed, more mathematical-based algorithms have been proposed such as Gold Sine Algorithm (GSA) [38], Exponential–Trigonometric Optimization (ETO) [39], Sinh Cosh Optimizer (SCHO) [40], Subtraction Average Based Optimizer (SABO) [41] and Arithmetic Optimization Algorithm (AOA) [42]. BIAs are inspired by social behaviors or characteristics of animals, plants, and humans. Animal-based algorithms are a large group of metaheuristic algorithms that solve optimization problems by modeling collaborative foraging, natural enemy avoidance, and migration among animals. The most classic of these are Particle Swarm Optimization (PSO) [43] and Ant Colony Optimization (ACO) [44]. Apart from those, there are some widely used animal-based algorithms such as Whale Optimization Algorithm (WOA) [45], Harris Hawks Optimization (HHO) [46], Cuckoo Search Algorithm (CSA) [47], and Butterfly Optimization Algorithm (BOA) [48]. In addition, several animal-based algorithms have recently been proposed, including but not limited to the Tuna Swarm Optimization (TSO) [49], Crayfish Optimization Algorithm (COA) [50], Secretary Bird Optimization Algorithm (SBOA) [51], Parrot Optimizer (PO) [52], Elk Herd Optimizer (EHO) [53], and Hippopotamus Optimization Algorithm (HOA) [54]. Plant-based algorithms are a class of meta-heuristic algorithms that have emerged in recent years, including Ivy Algorithm (IVYA) [55], Sunflower Optimization (SO) [56], Dandelion Optimizer (DO) [57]. Human-based algorithms derive from various types of human social behaviors and norms. Teaching–Learning-Based Optimization (TLBO) [58] is the most typical example of this category and is inspired by the process of knowledge transfer between teachers and students in the educational system. Human Memory Optimization Algorithm (HMOA) [59], Hiking Optimization Algorithm (HOA) [60], Preschool Education Optimization Algorithm (PEOA) [61], Catch Fish Optimization Algorithm (CFOA) [62] and Football Team Training Algorithm (FTTA) [63], and Gold Rush Optimizer (GRO) [64] are also in this category.

The Superb Fairy-wren Optimization Algorithm (SFOA) is a recently emerged animal-based meta-heuristic algorithm proposed by Jia et al. in 2024 [65]. SFOA focuses on modeling three different behaviors of the Superb Fairy-wren, which include juvenile growth, reproduction, and rearing, as well as avoidance of natural predators. The SFOA is demonstrated to have excellent optimization capability through experiments on 41 different functions, engineering constraint problems, and feature selection problems. However, SFOA has some shortcomings. Its switching mechanism is not enough to adapt to complex optimization problems, and it faces a weakening of population diversity in the late stage of optimization, leading to a higher possibility of falling into local optima. In addition, its global search capability needs to be improved. The shortcomings of SFOA motivate us to improve it. On the other hand, the no free lunch theory states that no single algorithm can satisfy all possible optimization problems. This is one of the motivations for us to improve SFOA.

Based on the above reasons, this paper provides appropriate improvements to SFOA for enhancing its performance. This work proposes ASFOA that integrates three improvement techniques. First, we propose an adaptive switching framework to dynamically select different update strategies to better adapt to different complex problems. We propose a three-factor bootstrapping strategy that utilizes the dominant group, the dominant individual, and the individual itself to jointly guide the population evolution, enhance the information exchange among individuals, and enrich the population diversity. In addition, the original danger threshold updating method is improved to make it more adaptable to the optimization process. In order to verify the performance of ASFOA, this paper compares it with different categories of classical/highly cited/newly proposed algorithms on the CEC2018 and CEC2022 test sets. Three statistical methods, which include the Friedman test, the Wilcoxon rank sum test, and the Nemenyi test, were applied to thoroughly analyze the experimental results. The main contributions of this paper are as follows:

(1) The ASFOA is proposed, integrating an adaptive switching framework, a three-factor synergistic guidance strategy, and an adaptive danger threshold updating strategy.

(2) An adaptive switching framework is designed to dynamically select search strategies that make SFOA adapt to different optimization problems.

(3) A three-factor synergistic guidance strategy is introduced to utilize individuals or groups with different characteristics for guidance, which can enrich population diversity and strengthen the global searchability.

(4) An adaptive danger threshold updating strategy is proposed to increase search diversity and improve algorithm robustness.

(5) ASFOA is compared with classical/highly cited/newly proposed metaheuristics on multiple test sets in different dimensions. The test results reveal the performance of ASFOA in dealing with numerous optimization problems.

The rest of this paper is organized as follows: The three different steps of SFOA are shown in Section 2. In Section 3, we present the framework and time complexity of the proposed ASFOA. Section 4 provides experimental results comparing classical/highly cited/newly proposed metaheuristics on different benchmark functions with different dimensions. In addition, 10 engineering-constrained optimization problems were used to test ASFOA’s ability to solve real-world optimization challenges. Finally, the conclusion and discussion are given in Section 5.

## 2. Superb Fairy-Wren Optimization Algorithm

A new animal-based metaheuristic algorithm, Superb Fairy-wren Optimization Algorithm, was proposed by Jia et al. in 2024. The SFOA simulates different behaviors of Superb Fairy-wrens, including young birds’ growth, breeding, and feeding, as well as avoiding natural enemies. The specific steps are as follows.

### 2.1. Random Initialization

Similarly to most animal-based metaheuristic algorithms, SFOA randomly generates initial populations in the problem space. The population consists of N members, and the initial position of $i^{th}$ member $X_{i}^{ini}$ is represented by Equation (1).

(1) $$X_{i}^{ini}=lb+rand\left(1,D\right)\times \left(ub-lb\right)$$

where ub and lb define the upper and lower search space bounds. $rand\left(1,D\right)$ is a vector of *D*-dimensional random numbers whose each element is uniformly distributed between 0 and 1.

### 2.2. Young Birds Growth Stage

The first stage of the SFOA is the young bird growth stage. In this phase, the population constantly updates the position of the young birds as they grow, enabling a global search. Specifically, SFOA utilizes Equation (2) to update the position of each member.

(2) $$X_{i}^{new}=X_{i}^{old}+rand\left(1,D\right)\times \left(ub-lb\right)+lb$$

where $X_{i}^{old}$ denotes the current position of the $i^{th}$ member and $X_{i}^{new}$ denotes its updated position.

### 2.3. Breeding and Feeding Stage

The breeding and feeding stage is the second stage of the SFOA. In this stage, the SFOA updates the positions of the population members by modeling the teaching mechanism of Superb Fairy-wren during breeding and feeding as shown in Equation (3).

(3) $$X_{i}^{new}=a\times X_{best}+\left(X_{best}-X_{i}^{old}\right)\times \sin\left(\left(ub-lb\right)\times \left(2+\frac{2FEs}{MaxFEs}\right)\right)$$

where a is a constant with a value of 0.8 according to the source literature. $X_{best}$ is the position of the global best member (in the case of the minimization problem, with the minimum fitness). FEs represents the number of fitness function evaluations. MaxFEs represents the max number of fitness function evaluations.

### 2.4. Avoiding Natural Enemies Stage

The final stage of the SFOA is the avoiding natural enemies stage. By simulating the defense mechanism of Superb Fairy-wren to avoid predation by natural enemies, SFOA updates the position of the population. This stage enhances the algorithm’s search range in the problem space and its ability to utilize local search, as shown in Equation (4).

(4) $$X_{i}^{new}=X_{best}+X_{i}^{old}\times l\times b\times \sin\left(\frac{\pi }{2}\times \left(1-\frac{FEs}{MaxFEs}\right)\right)$$

where l is a *D*-dimensional random vector obeying the Levy distribution. b is a constant with value 0.2.

### 2.5. Implementation of SFOA

At each iteration, SFOA selects one of three stages to be used for population updating. The young birds’ growth stage is mainly used for global exploration. The breeding and feeding stage prefers local exploitation, and the avoiding natural enemies stage is used to increase the randomness of the SFOA’s exploration in space. For SFOA, the probability of executing the young birds’ growth stage is the same as the probability of executing the breeding and feeding stage, as well as the avoiding natural enemies stage. Therefore, we decide which stage to execute by judging the size of $r_{1}$ and 0.5 at each iteration. When $r_{1}$ > 0.5, the SFOA executes the young birds’ growth stage; conversely, the SFOA executes one of the remaining two stages. Further, for the breeding and feeding stage and the avoiding natural enemies stage, the SFOA selects according to the magnitude of the danger threshold s, as shown in Equation (5). Specifically, when s > 20, the avoiding natural enemies stage is executed. Conversely, the breeding and feeding stage is applied. The execution mechanism of SFOA is shown in Equation (6).

(5) $$s=r_{2}\times 20+r_{3}\times 20$$

(6) $$X_{i}^{new}=\left\{\begin{array}{l} X_{i}^{old}+rand\left(1,D\right)\times \left(ub-lb\right)+lb,r_{1}>0.5\\ a\times X_{best}+\left(X_{best}-X_{i}^{old}\right)\times \sin\left(\left(ub-lb\right)\times \left(2+\frac{2FEs}{MaxFEs}\right)\right),\;r_{1}\leq 0.5\;and\;s\leq 20\\ X_{best}+X_{i}^{old}\times l\times b\times \sin\left(\frac{\pi }{2}\times \left(1-\frac{FEs}{MaxFEs}\right)\right),\;r_{1}\leq 0.5\;and\;s>20 \end{array}\right.$$

where $r_{1}$ is a random number in [0,1] range. Both $r_{2}$ and $r_{3}$ are random numbers with a standard normal distribution.

## 3. The Proposed ASFOA

Although the performance of SFOA has been proven, it is still restricted when facing complex problems. In order to lift the performance limitation of SFOA, this section combines three strategies to tune SFOA and proposes ASFOA. The details are as follows.

### 3.1. Adaptive Switching Framework (ASF)

In metaheuristic algorithms, the ability to balance exploration and exploitation is the key to the algorithm. WOA and Grey Wolf Optimizer (GWO) [66] realize the switch from exploration to exploitation by controlling the parameters, but this linear approach is not applicable to all problems. For SFOA, it uses random selection to switch between exploitation and exploration. This approach is somewhat favorable to balance exploitation and exploration, but too random selection may lead the algorithm to explore when it needs to explore and exploit when it needs to explore. Therefore, it is necessary to design a switching mechanism to assist the SFOA in choosing the appropriate search behavior at the appropriate stage. To this end, this paper proposes an adaptive switching framework (ASF), which aims to dynamically select the search strategy according to the search process. The framework is able to adaptively select different strategies based on their historical success rates as follows.

(7) $$Sw_{f}^{new}=Sw_{f}^{old}+\frac{0.05\times \left(1-Sw_{f}^{old}\right)\times \left(\frac{S1_{better}}{S1}-\frac{S23_{better}}{S23}\right)\times FEs}{MaxFEs}$$

where $Sw_{f}^{new}$ is the adaptive switching parameter. S1 denotes the number of members executing the young birds growth stage. $S1_{better}$ denotes the number of members with better fitness after executing the young birds’ growth stage. S23 denotes the number of members that performed the breeding and feeding stage and the avoiding natural enemies stage. $S23_{better}$ denotes the number of members whose fitness became better after performing the breeding and feeding stage and the avoiding natural enemies stage. In ASFOA, the initial value of $Sw_{f}^{ini}$ is set to 0.5 since there is no a priori knowledge. Moreover, if $Sw_{f}^{new}$ is outside of [0.1, 0.9], it will be replaced by the limit value (0.1 or 0.9) closest to the generated value.

### 3.2. Three-Factor Synergistic Guidance Strategy (TSG)

SFOA conducts global exploration through Equation (2). The analysis shows that Equation (2) expands the search range by applying a random perturbation to each member. However, it ignores the possibility that populations have the potential for rapid clustering at later stages, which can severely reduce population diversity. In addition, during global exploration, this blind search ignores the exchange of information between individuals, making it difficult for the population to find promising regions to search. Since SFOA may execute Equation (2) throughout the search process, this weakens the convergence ability. To solve the above problems, this paper proposes a three-factor synergistic guidance strategy (TSG). In this paper, we replace the young birds’ growth stage with TSG. Specifically, TSG utilizes valid information from dominant groups, dominant individuals, and individuals themselves. The dominant group is used to build the covariance matrix. By establishing a feature coordinate system for the current population, the algorithm’s dependence on the original fixed coordinate system is reduced and the algorithm’s rotation is enhanced without deformation, thereby enhancing the algorithm’s performance in solving indivisible problems (i.e., problems that are difficult to decompose into independent subproblems or localized problems with a high degree of coupling between different parts of the problem). The dominant individual is a randomly selected individual from the top three individuals in terms of fitness, which effectively ensures convergence ability while avoiding falling into a local optimum to some extent. The information about the individual itself is used to move from the current position, expanding the search range. The mathematical model of TSG is denoted as follows.

(8) $$X_{i}^{new}=\frac{\left(X_{i}^{old}+X_{wmean}+X_{better}\right)}{3}+G_{i},G_{i}\sim N\left(0,Cov\right)$$

(9) $$Cov=\frac{1}{0.5N}\sum_{i=1}^{\left|Q\right|}\left(X_{i}^{Q}-X_{wmean}\right)\times {\left(X_{i}^{Q}-X_{wmean}\right)}^{T}$$

(10) $$X_{wmean}=\sum_{i=1}^{\left|Q\right|}\omega_{i}\times X_{i}^{Q}$$

(11) $$\omega_{i}=\ln\left(\left|Q\right|+1\right)/\left(\sum_{i=1}^{\left|Q\right|}\left(\ln\left(\left|Q\right|+1\right)-\ln\left(i\right)\right)\right)$$

where $X_{wmean}$ is the weighted average position of the dominant group. $X_{better}$ is a member randomly selected from the top three individuals with the best fitness. Cov is the covariance matrix based on the dominant group. $\left|Q\right|$ is the number of the dominant group, which in this paper consists of the top half of the individuals with the best fitness. $X_{i}^{Q}$ denotes the members in the dominant group. $\omega_{i}$ is the weighting coefficient of each individual, which denotes the degree of influence on $X_{wmean}$.

### 3.3. Adaptive Danger Threshold Updating Strategy (ADS)

**Figure 2:**
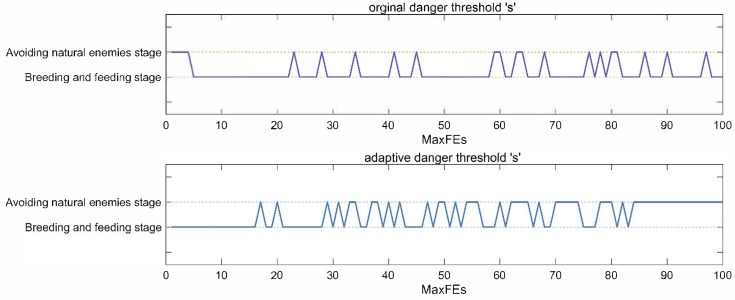
Visualization of changes in danger threshold *s*.

The SFOA switches between the Breeding and Feeding stage and the Avoiding natural enemies stage by calculating the danger threshold s. The second stage of the SFOA focuses on precision exploitation, while the third stage allows for a wider search range and enhanced exploitation. This stage can help it escape from local optimization, which is an advantage brought by Levy flight. However, as shown in Figure 2, SFOA performs breeding and feeding stage more throughout the search process, which is not conducive to its escape from local optimality. Therefore, we designed an Adaptive Danger Threshold Update Strategy (ADS). As shown in Figure 2, the new danger threshold updating strategy increases the frequency of executing the third stage; meanwhile, in the later stage, more third stages can effectively help SFOA jump out of the local optimum. Therefore, we use Equation (12) to calculate the danger threshold s.

(12) $$s=\frac{FEs}{MaxFEs}\times 20+r_{4}\times 20$$

where $r_{4}$ is a random number in [0,1] range.

### 3.4. The Pseudo-Code and Flowchart for ASFOA

**Figure 3:**
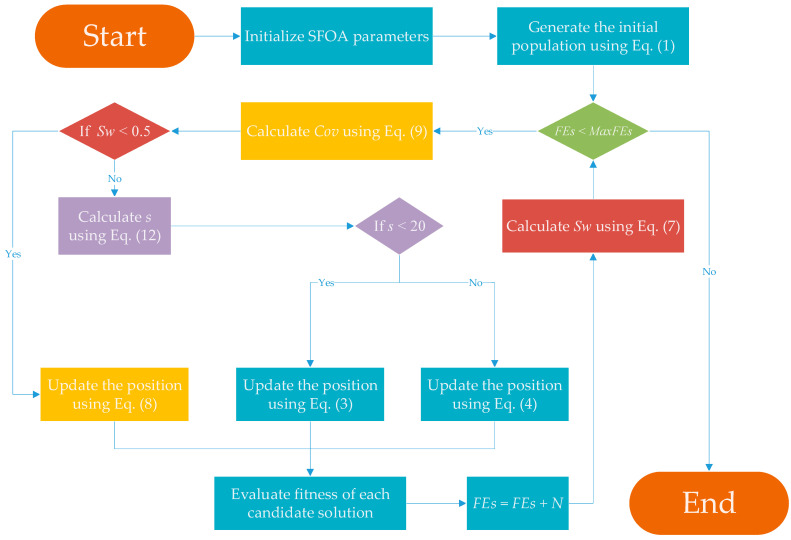
Flowchart of the proposed ASFOA.

To show the specific steps of ASFOA more clearly, its pseudo-code and flowchart are shown here, as shown in Algorithm 1 and Figure 3.
**Algorithm 1** Pseudocode of ASFOA1. Input: The number of candidate solution *N*, Dimension *D*, The max number of fitness evaluation *MaxFEs*2. Output: The best candidate solution *X*_best_3. Generate the initial population using Equation (1)4. **While** *FEs* < *MaxFEs*5.      Calculate *Cov* using Equation (9)6.      **For** *i* = 1: *N*7.              **If** $Sw_{f}^{new}$ < 0.58.                   Update the position using Equation (8)//Three-factor synergistic guidance strategy9.              **Else**10.                   Calculate *s* using Equation (12)//Adaptive danger threshold updating strategy11.                   **If** *s* < 2012.                          Update the position using Equation (3)//Breeding and feeding stage13.                   **Else**14.                          Update the position using Equation (4)//Avoiding natural enemies stage15.                   **End if**16.              **End if**
17.      **End for**18.      Evaluate fitness of each candidate solution19:      *FEs* = *FEs* + *N*20.      Calculate $Sw_{f}^{new}$ using Equation (7)//Adaptive switching framework21. **End while**

### 3.5. The Time Complexity of ASFOA

Time complexity analysis is used to evaluate the computational cost of an algorithm. For SFOA and ASFOA, the time complexity depends on three factors: the problem dimension *D*, the number of populations *N*, and the number of iterations *T*.

For SFOA, the time complexity of population initialization is $O\left(N\times D\right)$. At each iteration, SFOA updates the population using only one strategy, so the time complexity of updating the population is $O\left(N\times D\times T\right)$. In sum, the time complexity of SFOA is $O\left(N\times D\times \left(T+1\right)\right)$. If we ignore the fixed number, the total time complexity of SFOA is $O\left(N\times D\times T\right)$. The same population initialization method is used by ASFOA without increasing the time complexity. For the three improved strategies integrated by ASFOA, ASF and ADS replace the original selection mechanism without changing the time complexity. Although TSG replaces the original young birds’ growth stage, it adds no additional fitness evaluation and is still selected along with the other two update strategies. Therefore, ASFOA has the same time complexity of $O\left(N\times D\times T\right)$ as SFOA.

## 4. Experimental Results and Discussion

### 4.1. Benchmark Functions and Setup of Numerical Experiments

In this section, the performance of ASFOA is tested on CEC 2018 [67] and CEC 2022 [68] benchmark test suites. The CEC2018 test functions consist of 29 minimization problems, which can be divided into 4 groups: unimodal functions (F1–F2), multimodal functions (F3–F9), hybrid functions (F10–F19), and composition functions (F20–F29). The CEC 2022 test functions consist of unimodal functions (F1), basic functions (F2–F5), hybrid functions (F6–F8), and composition functions (F9–F12). Optimization problems with *D* = [10,30,50,100] from the CEC 2018 test suite, *D* = [10,20] from the CEC2022 test suite are considered. The search range of [−100,100] *^D^* is used. A broad summary of these test functions is presented in Table 1 and Table 2.

In order to fully demonstrate the performance of ASFOA, a series of comparison experiments is conducted. First, ASFOA and six derived algorithms integrating a single strategy or two strategies are subjected to ablation experiments on the CEC2018 test set. Second, ASFOA is compared with eight classical/highly cited/newly proposed metaheuristics on the CEC2018 test set and the CEC2022 test set to fully illustrate the superiority of ASFOA. Details of the derived algorithms are shown in Section 4.2. The eight comparison algorithms chosen include the classic and influential algorithms LSHADE [69], the highly cited algorithms MPA [70], EO [71], the newly proposed EDO [72], AE [73], RIME [74], ECO [75], BKA [76]. These comparison algorithms come from different classes of metaheuristics, and by comparing them across a wide range of classes, the superior performance of ASFOA is better demonstrated. Table 3 shows the parameter settings for the algorithms considered; their controlling parameters are set at the values recommended in their literature, except for population size (N) and the max number of fitness function evaluations (MaxFEs). All experiments were run on a computer equipped with MATLAB 2021b, an AMD R9 9950X processor running at 4.3 GHz, and 48 GB of RAM. To ensure a fair comparison, the population size was set at 30*D*, and every algorithm was executed on the basis of 30 independent runs, in line with protocols established in previous investigations. The maximum number of function evaluations was fixed at 1000*D*.

In this paper, we will record the optimal value (Best), standard deviation (Std), and average value (Mean) obtained by all the algorithms. The average value reflects the average performance of the algorithms over 30 independent runs, with lower fitness values representing higher quality solutions in minimization problems. However, the average levels may be affected by outliers; the standard deviation is introduced to analyze the stability of runs. A small standard deviation reveals less volatility and strong robustness of the algorithm. In view of the above evaluation metrics, if an algorithm is characterized by a better mean objective function value and a small standard deviation, the algorithm is considered stable and excellent. The complete data is obtained in Appendix A. In the analysis of the main section, we primarily use the Wilcoxon rank sum test, the Friedman test, and the Nemenyi test to analyze the differences between ASFOA and the comparison algorithms. Among them, the Wilcoxon rank sum test can analyze the variability of ASFOA and comparison algorithms on different functions. The Friedman test is used to analyze the overall performance difference between ASFOA and comparison algorithms. The Nemenyi test is used as a post hoc test to further evaluate the magnitude of difference between ASFOA and comparison algorithms.

### 4.2. Ablation Experiments Using CEC 2018 Test Suite

SFOA achieves its performance gains by integrating three improvement strategies. In order to evaluate the impact of these improvement techniques on SFOA and the compatibility between them, six different variants of SFOA are developed in this section, as shown in Table 4. Y indicates that the SFOA integrates the strategy, and N indicates that the SFOA does not employ the strategy.

The experimental results obtained by ASFOA, SFOA, and the derived algorithms on the CEC2018 test set are recorded in Table A1, Table A2, Table A3 and Table A4 in Appendix A. ASFOA achieves the minimum Ave for all functions on 30*D* and 100*D* and obtains the minimum Ave for 27 (28) functions on 10*D* (50*D*). Compared to SFOA and the derived algorithms, ASFOA exhibits the ability to consistently deliver high-quality solutions.

**Figure 4:**
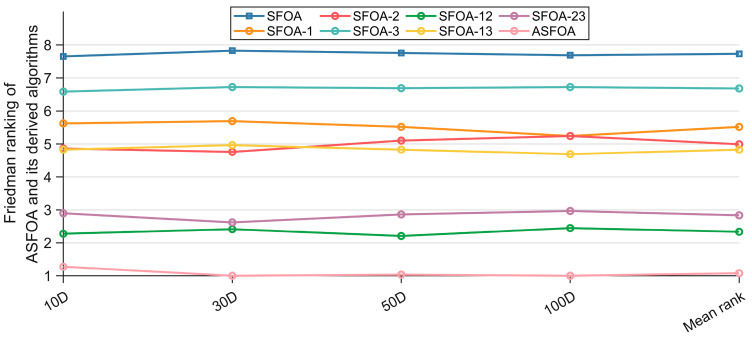
Friedman rankings of ASFOA, SFOA, and their derived algorithms.

**Figure 5:**
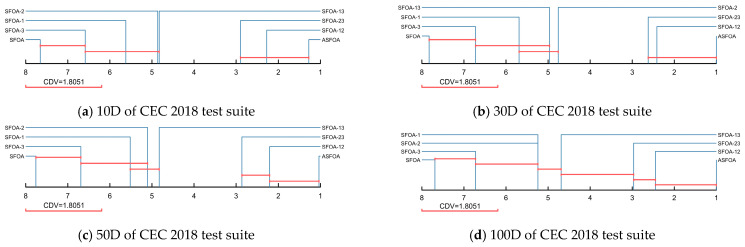
Multiple comparisons using CDV to evaluate ASFOA, SFOA, and their derived algorithms.

Table 5 summarizes the results of the Friedman test for ASFOA, SFOA, and the derived algorithms, which are obtained with a significance level of a = 0.05. In Table 5, “Mean rank” denotes the average rank under the four dimensions, and “Overall rank” denotes the order of the average rank. As shown in Table 5, the *p*-values for all four dimensions are less than 0.05, which indicates that there is a performance difference between ASFOA, SFOA, and the derived algorithms. The Friedman rankings of ASFOA, SFOA, and derived algorithms are visualized in Figure 4. To further measure the difference between ASFOA, SFOA, and the derived algorithms, we conducted a post hoc test using the Nemenyi test. Figure 5 presents the magnitude of differences among ASFOA, SFOA, and their derived algorithms, in which the algorithms with no significant difference in terms of performance can be connected using CDV. A discussion based on Figure 4 and Figure 5 is presented below.

The influence of ASF on ASFOA can be investigated by comparing ASFOA, SFOA-1, SFOA-23, and SFOA. ASFOA is superior to SFOA-23 in four dimensions, which indicates that ASF can enhance the performance of SFOA. SFOA-1 is superior to basic SFOA in four dimensions, which also proves that ASF can enhance the performance of SFOA. Also, according to the results in Figure 5, there is a significant difference between the performance of SFOA-1 and basic SFOA, which indicates that the enhancement effect of ASF is obvious. There is no significant difference between SFOA-23 and ASFOA in 10D/30D, which indicates that the enhancement of the performance by ASF in high-dimensional problems is greater than the enhancement of the performance in low-dimensional problems. In conclusion, ASF is effective.

The influence of TSG on ASFOA can be investigated by comparing ASFOA, SFOA-2, SFOA-13, and SFOA. ASFOA is superior to SFOA-13 in all four dimensions, which indicates that TSG can enhance the performance of SFOA. SFOA-2 is superior to basic SFOA in all dimensions, which also proves that TSG can enhance the performance of SFOA. According to the results in Figure 5, there is a significant difference between the performance of SFOA-2 and basic SFOA, which indicates that the enhancement effect of TSG is obvious. There is a significant difference between SFOA-23 and ASFOA in all dimensions, which suggests that TSG can positively affect SFOA in both low and high latitudes. In conclusion, TSG is effective.

The influence of ADS on ASFOA can be investigated by comparing ASFOA, SFOA-3, SFOA-12, and SFOA. ASFOA is superior to SFOA-12 in all four dimensions, which indicates that ADS can enhance the performance of SFOA. SFOA-3 is superior to the basic SFOA in all four dimensions, which also proves that ADS can enhance the performance of SFOA. However, according to the results in Figure 5, there is no significant difference between SFOA-3 and basic SFOA, which indicates that the enhancement effect of ADS is limited. There is no difference between SFOA-12 without ADS and ASFOA in all dimensions, which also indicates that the contribution of ADS to ASFOA is limited and cannot significantly enhance SFOA. In conclusion, ADS has a positive effect on SFOA, but not significant enough.

Overall, all three improvement strategies can enhance the performance of SFOA.TSG contributes the most to ASFOA, followed by ASF, and ADS contributes the least to ASFOA. Meanwhile, we can conclude that the three improvement strategies can be compatible and jointly enhance SFOA.

### 4.3. Comparison Experiments Using CEC 2018 Test Suite

**Figure 6:**
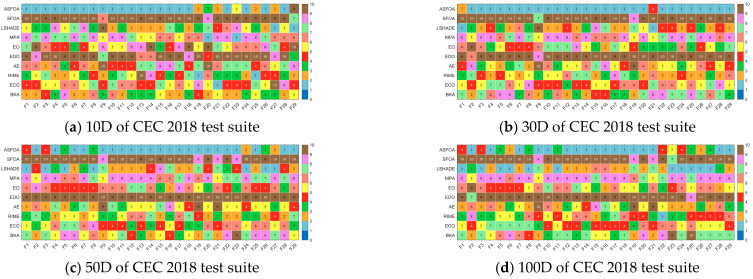
Ave-based rankings of ASFOA and comparison algorithms.

In this subsection, we compare ASFOA with eight comparison algorithms on the CEC 2018 test suite, and the complete results obtained are summarized in Table A5, Table A6, Table A7 and Table A8 in Appendix A. Among them, ASFOA obtained the smallest Ave on 22/26/22/17 test functions for 10*D*/30*D*/50*D*/100*D*, which indicates that ASFOA is able to provide satisfactory results on most of the functions with efficient solving ability. The Ave-based rankings of ASFOA and comparison algorithms on the CEC 2018 test set are shown in Figure 6.

**Figure 7:**
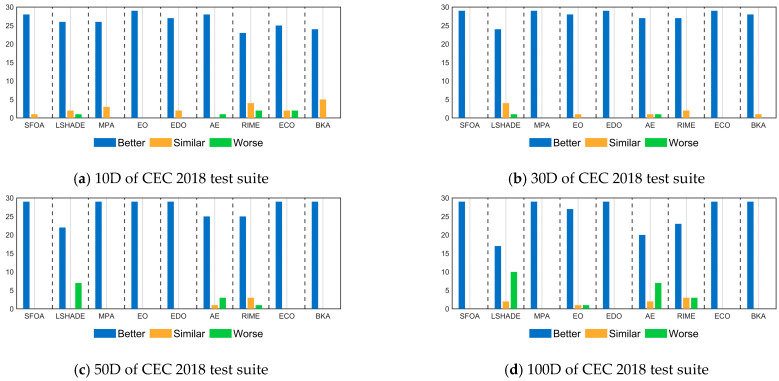
The visualization of Wilcoxon rank sum test results of ASFOA and comparison algorithms.

Table 6 shows the results of the Wilcoxon rank sum test for ASFOA and LSHADE, MPA, EO, EDO, AE, RIME, ECO, and BKA based on their experimental data on the CEC2018 test set. The Wilcoxon rank sum test is a nonparametric pairwise test used to assess significant differences in the performance of two algorithms. The returned *p* values represent whether to accept the original hypothesis (*p* > 0.05) or deny it (*p* < 0.05). The results of the test for each dimension this article selected, shown in Table 4, indicate that ASFOA is significantly better or worse with symbols ‘+’ and ‘−’ than other algorithms, and ‘=’ was used to denote indistinguishable results of two algorithms. Figure 7 visualizes the Wilcoxon rank sum test results for ASFOA and LSHADE, MPA, EO, EDO, AE, RIME, ECO, BKA. A detailed analysis is as follows.

For 10*D*, ASFOA is better (worse) than SFOA, LSHADE, MPA, EO, EDO, AE, RIME, ECO, BKA on 28(0), 26(1), 26(0), 29(0), 27(0), 28(1), 23(2), 25(2), 24(0) benchmark functions. That is, ASFOA is dominant in at least 23 functions when compared to different algorithms when solving 10*D* functions.

For 30*D*, ASFOA is better (worse) than SFOA, LSHADE, MPA, EO, EDO, AE, RIME, ECO, BKA on 29(0), 24(1), 29(0), 28(0), 29(0), 27(1), 27(0), 29(0), 28(0) benchmark functions. That is, ASFOA is dominant in at least 27 functions when compared to different algorithms when solving 30*D* functions.

For 50*D*, ASFOA is better (worse) than SFOA, LSHADE, MPA, EO, EDO, AE, RIME, ECO, BKA on 29(0), 22(7), 29(0), 29(0), 29(0), 25(3), 25(1), 29(0), 29(0) benchmark functions. That is, ASFOA is dominant in at least 22 functions when compared to different algorithms when solving 50*D* functions.

For 100*D*, ASFOA is better (worse) than SFOA, LSHADE, MPA, EO, EDO, AE, RIME, ECO, BKA on 29(0), 17(10), 29(0), 27(1), 29(0), 20(7), 23(3), 29(0), 29(0) benchmark functions. That is, ASFOA is dominant in at least 17 functions when compared to different algorithms when solving 100*D* functions.

According to Figure 7, we can learn that ASFOA, although the gap with some of the compared algorithms narrows when facing high-dimensional problems, still has a significant advantage in more than half of the functions. Therefore, we can conclude that the overall performance of ASFOA is superior to SFOA, LSHADE, MPA, EO, EDO, AE, RIME, ECO, BKA.

**Figure 8:**
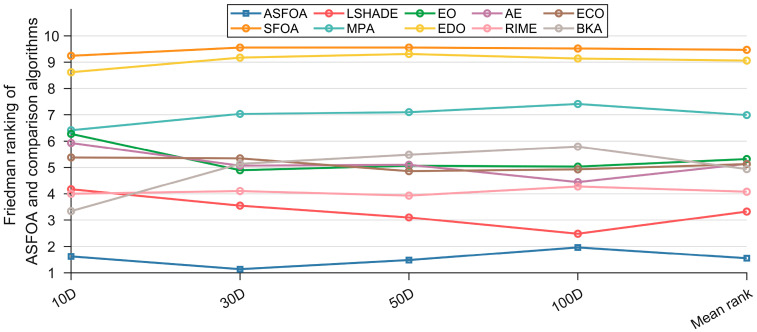
Friedman rankings of ASFOA and competition algorithms based on CEC 2018 test suite.

The results of the Wilcoxon rank sum test fully demonstrate that ASFOA provides great performance in terms of solution quality. To avoid transitivity between the results, Friedman test ranks are used for multiple samples. As reported in Table 7 and Figure 8, AROACS has a significant lead in the average ranking for all sets of benchmark functions and different dimensions. This situation indicates that ASFOA achieves better solutions on most benchmark functions compared to the remaining nine algorithms, or most consistently maintains higher overall performance in each set of tests. ASFOA’s ranking fluctuates as the dimensions change, but the individual dimensions are still ranked number one, which indicates that ASFOA scales well. Although ASFOA’s performance drops slightly when facing high-dimensional problems, it still maintains its advantage. In general, many meta-heuristic algorithms face a decrease in efficiency or solution quality, which is due to the increase in dimensionality leading to the expansion of the search space. Notably, LSHADE shows better performance in high dimensions, again emphasizing its status as a classical algorithm. Overall, ASFOA has excellent search capability, stability, and adaptability.

**Figure 9:**
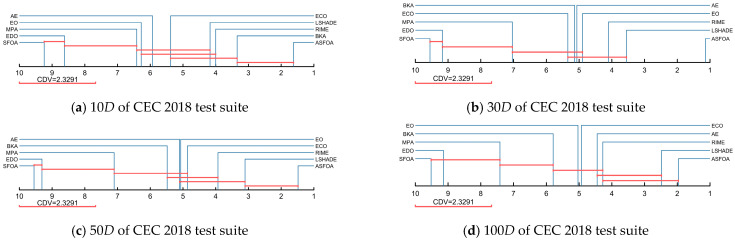
Multiple comparisons using CDV to evaluate ASFOA and its competition algorithms.

Similarly, we further evaluate the magnitude of the difference between ASFOA and LSHADE, MPA, EO, EDO, AE, RIME, ECO, BKA by using the Nemenyi test mentioned in Section 4.2, as shown in Figure 9, where the level of significance is a = 0.05. When solving the 10*D* problems, ASFOA shows a significantly superior performance to LSHADE, MPA, EO, EDO, AE, RIME, ECO, but no significant differences with BKA. When facing the 30*D* functions, ASFOA rides high and is significantly different from the other compared algorithms. For the 50*D* and 100*D* functions, there is no significant difference between ASFOA and LSHADE, but ASFOA has a significant superiority compared to other algorithms, except RIME (100*D*). Overall, by analyzing the experimental results of ASFOA and the comparison algorithms using the Friedman test and the Nemenyi post hoc test, we can conclude that ASFOA has significant performance advantages and is a promising variant of the metaheuristic algorithm.

### 4.4. Convergence and Stability Analysis Using CEC 2022 Test Suite

**Figure 10:**
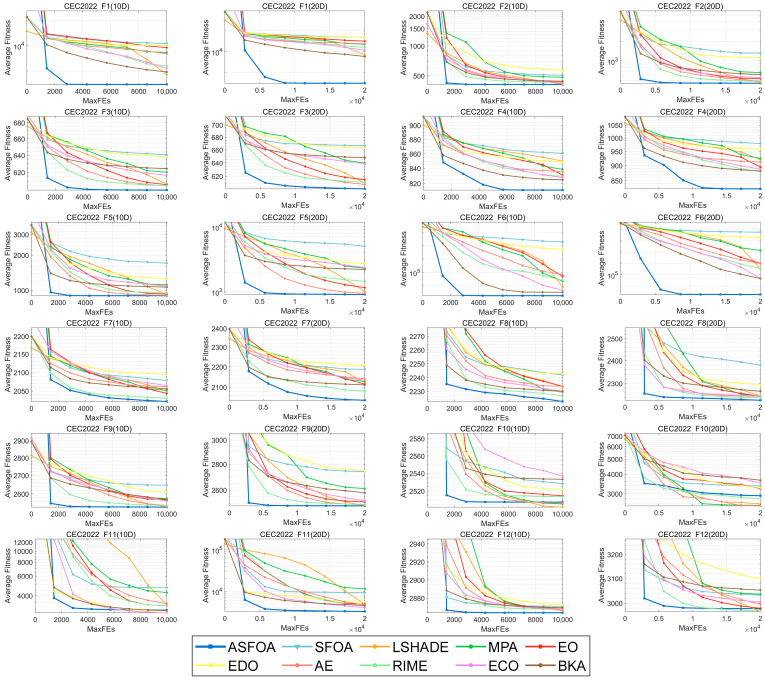
Convergence curves of ASFOA and its competition algorithms on all functions from CEC 2022 test suite.

**Figure 11:**
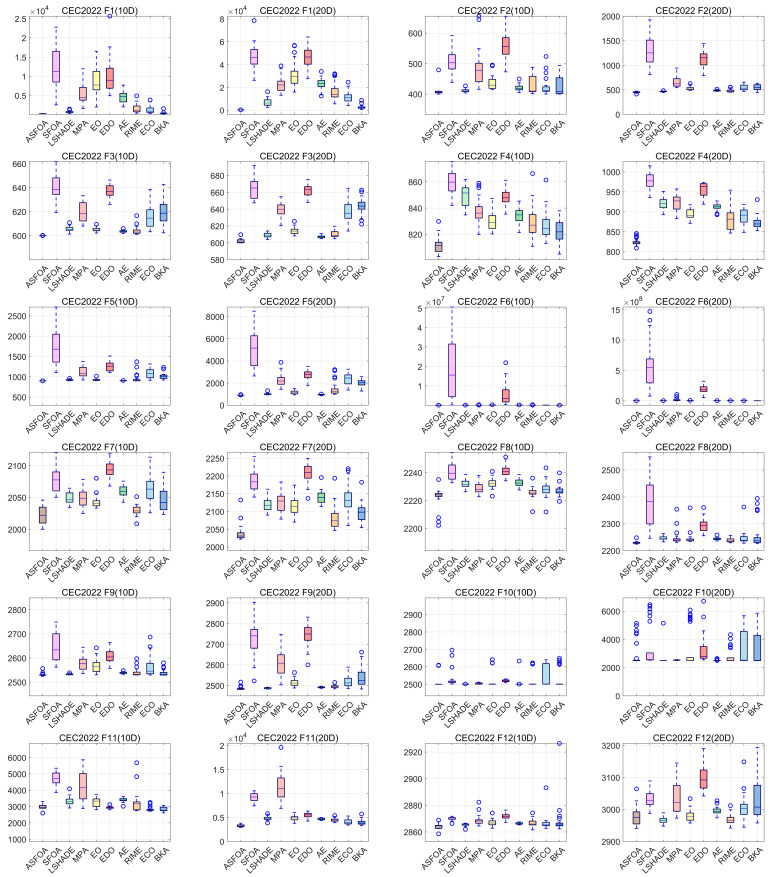
Boxplot charts of ASFOA and its competition algorithms on all functions from CEC 2022 test suite.

In this subsection, we analyze the convergence and robustness of ASFOA. To fully evaluate the performance of ASFOA, the CEC2022 test set is used for the experiments in this subsection. Figure 10 provides the convergence curves of the 10 algorithms for all functions on the CEC2022 benchmark with *D* = 10/20. The curves show the average best solutions obtained in 30 independent runs of each algorithm. To discuss the stability of the algorithm solution results, the corresponding boxplots are shown in Figure 11. The convergence curve shows the trend of the objective function value of the metaheuristic algorithms as the number of iterations changes during the optimization process. Boxplots are used to show the performance distribution of multiple algorithms in different test functions and multiple runs. Each box represents the statistical distribution of an algorithm, including the median (middle line), upper and lower quartiles (top and bottom edges of the box), maximum and minimum values (whiskers), and possible outliers (points).

As shown in Figure 10, the curves of ASFOA perform best on the functions F1-F6, F8-F9 for 10*D*/20*D*, F11 for 20*D*, and F12 for 10*D*, which converge to a near-optimal solution in the early iterations and continue to extend downward in the later stages. ASFOA jumps out of the local optimality trap into which most of the other algorithms fall. It is worth noting that ASFOA’s curve outperforms SFOA’s curve with higher convergence speed and accuracy. For both dimensions of F7, ASFOA does not converge to the optimal value early on but is able to optimize consistently and shows stable search capability.

It can be seen in Figure 11, the boxplots of ASFOA contain a lower median and box position, narrower height of the box, and fewer outliers, which show the higher stability and convergence accuracy of ASFOA compared to the other nine algorithms. The ASFOA provides acceptable and favorable solutions in terms of a balance between exploration and exploitation, which shows that it has full potential for real-world application.

### 4.5. Comparison Experiments Using Engineering Constrained Optimization Problems

To further evaluate the capability of ASFOA in real-world optimization scenarios, it was compared to other algorithms in 10 engineering-constrained optimization problems. Table 8 summarizes these engineering constraint optimization problems. Engineering constrained optimization problems are often difficult to solve due to multiple constraints with highly nonlinear objective functions, and a variety of constraint handling techniques have been proposed in the academic community, including superiority of feasible solutions, penalty functions, adaptive penalty strategies, and ε- constraint handling, etc. In this paper, the penalty function method is used: a sufficiently large penalty is imposed once the solution violates the constraints, thus transforming the original problem into unconstrained optimization. Table 9 gives the optimum, mean, standard deviation, and ranking of the 30 independent runs.

The results in Table 9 show the effectiveness of the ASFOA in solving real optimization problems, proving its power. It is worth noting that the ASFOA outperforms the other comparative algorithms on the nine constrained optimization problems and is inferior to the AE algorithm only on RW09. We further evaluate the differences between the ASFOA and the other algorithms using Friedman’s test and the Wilcoxon rank sum test. Among them, the ASFOA achieved a Friedman ranking of 1.500, and the basic SFOA ranked the worst at 9.300. The results of the Wilcoxon rank sum test also showed a significant dominance of ASFOA in at least eight real-world problems, exhausting a significant weakness to the AE algorithm on RW09. In conclusion, ASFOA successfully solves the real-world constraint challenges and exhibits satisfactory performance. Its overall performance is highly competitive compared to other algorithms.

## 5. Conclusions

In this work, the ASF, TSG, and ADS strategies are integrated into SFOA to improve its search capability. The proposed ASFOA is fully analyzed in the CEC2018 test set, including ablation experiments and comparative tests to verify its feasibility and superiority. In addition, the ASFOA is further validated on the CEC2022 test set to examine its convergence and robustness. Finally, the ability of ASFOA to solve engineering-constrained optimization problems is demonstrated in Section 4.5. Ablation experiments were performed in Section 4.2 to assess the impact of different improvement strategies on ASFOA. As shown in Table 3, ASFOA achieved a Friedman ranking of 1.078, which is ranked first. This indicates that ASFOA combining ASF, TSG, and ADS strategies has the best optimization capability. In addition, the Nemenyi test in Figure 5 further illustrates that there is a better compatibility between the three improved strategies. In Section 4.3, the performance of ASFOA and SFOA, LSHADE, MPA, EO, EDO, AE, RIME, ECO, BKA is thoroughly examined in the CEC2018 test set, and the experimental results are statistically analyzed by the Wilcoxon rank sum test, the Friedman test, and the Nemenyi post hoc test. The results indicate that ASFOA has the best overall performance. In Section 4.4, we further examine the convergence and robustness of ASFOA using the CEC2022 test set. The results indicate that ASFOA has good convergence speed and convergence accuracy, and can consistently provide high-quality solutions. However, ASFOA has some shortcomings. From the experimental results in Section 4.2, we can see that although ASFOA has the best overall performance, its performance decays more when facing high-dimensional problems, which indicates that we need to further improve its ability to solve high-dimensional complex problems. In addition, it can be seen from Figure 10 and Figure 11 that the convergence accuracy of ASFOA is not the best when dealing with the F9 and F10 functions. When solving F4 and F10, the solution of ASFOA is not concentrated enough and has more bad values. This indicates that the stability of ASFOA needs to be further strengthened, especially when facing complex problems.

In our future work, we will consider the following aspects. First, we try to enhance the ability of ASFOA to solve high-dimensional problems by introducing new mechanisms, e.g., applying a large language model to design new search mechanisms. Second, we will develop binary and multi-objective versions of ASFOA for solving more types of optimization problems. Finally, we plan to extend the application scenarios of ASFOA, such as UAV mission planning and cloud resource scheduling problems.

## Figures and Tables

**Table 1:** Detailed description of CEC2018 test functions.

Type	ID	Functions Name	Fmin
Unimodal functions	F1	Shifted and Rotated Bent Cigar Function	100
	F2	Shifted and Rotated Zakharov Function	300
Multimodal functions	F3	Shifted and Rotated Rosenbrock’s Function	400
	F4	Shifted and Rotated Rastrigin’s Function	500
	F5	Shifted and Rotated Expanded Scaffer’s F6 Function	600
	F6	Shifted and Rotated Lunacek Bi_Rastrigin Function	700
	F7	Shifted and Rotated Non-Continuous Rastrigin’s Function	800
	F8	Shifted and Rotated Levy Function	900
	F9	Shifted and Rotated Schwefel’s Function	1000
Hybrid functions	F10	Hybrid Function 1 (N = 3)	1100
	F11	Hybrid Function 2 (N = 3)	1200
	F12	Hybrid Function 3 (N = 3)	1300
	F13	Hybrid Function 4 (N = 4)	1400
	F14	Hybrid Function 5 (N = 4)	1500
	F15	Hybrid Function 6 (N = 4)	1600
	F16	Hybrid Function 6 (N = 5)	1700
	F17	Hybrid Function 6 (N = 5)	1800
	F18	Hybrid Function 6 (N = 5)	1900
	F19	Hybrid Function 6 (N = 6)	2000
Composition functions	F20	Composition Function 1 (N = 3)	2100
	F21	Composition Function 2 (N = 3)	2200
	F22	Composition Function 3 (N = 4)	2300
	F23	Composition Function 4 (N = 4)	2400
	F24	Composition Function 5 (N = 5)	2500
	F25	Composition Function 6 (N = 5)	2600
	F26	Composition Function 7 (N = 6)	2700
	F27	Composition Function 8 (N = 6)	2800
	F28	Composition Function 9 (N = 3)	2900
	F29	Composition Function 10 (N = 3)	3000

**Table 2:** Detailed description of CEC2022 test functions.

Type	ID	Functions Name	Fmin
Unimodal functions	F1	Shifted and full Rotated Zakharov Function	300
Basic functions	F2	Shifted and full Rotated Rosenbrock’s Function	400
	F3	Shifted and full Rotated Expanded Schaffer’s f6 Function	600
	F4	Shifted and full Rotated Non-Continuous Rastrigin’s Function	800
	F5	Shifted and full Rotated Levy Function	900
Hybrid functions	F6	Hybrid Function 1 (N = 3)	1800
	F7	Hybrid Function 2 (N = 6)	2000
	F8	Hybrid Function 3 (N = 5)	2200
Composition functions	F9	Composition Function 1 (N = 5)	2300
	F10	Composition Function 2 (N = 4)	2400
	F11	Composition Function 3 (N = 5)	2600
	F12	Composition Function 4 (N = 6)	2700

**Table 3:** Parameter settings for competing algorithm.

Algorithm	Parameters Setting
ASFOA	$a=0.8,b=0.2,\left|Q\right|=0.5N,Sw_{f}^{ini}=0.5$
SFOA	a=0.8,b=0.2
LSHADE	$F=0.5,Cr=0.5,p=0{.11,N}_{\min}=4$
MPA	FADs=0.2,P=0.5
EO	$a_{1}=2,a_{2}=1,GP=0.5$
EDO	α=rand
AE	λ=0.5
RIME	W=5
ECO	β=1.5,H=0.5,G=0.2,P=0.1
BKA	p=0.9,a=2

**Table 4:** Various SFOA variants with different strategies.

Strategy	SFOA-1	SFOA-2	SFOA-3	SFOA-12	SFOA-13	SFOA-23	ASFOA
ASF	Y	N	N	Y	Y	N	Y
TSG	N	Y	N	Y	N	Y	Y
ADS	N	N	Y	N	Y	Y	Y

**Table 5:** Friedman test results for ASFOA and derived algorithms based on CEC 2018 test suite.

Test Suite	Dimension	SFOA	SFOA-1	SFOA-2	SFOA-3	SFOA-12	SFOA-13	SFOA-23	ASFOA	*p*-Value
CEC 2018	10	7.655	5.621	4.862	6.586	2.276	4.828	2.897	1.276	7.66E-32
	30	7.828	5.690	4.759	6.724	2.414	4.966	2.621	1.000	4.63E-36
	50	7.759	5.517	5.103	6.690	2.207	4.828	2.862	1.034	4.66E-35
	100	7.690	5.241	5.241	6.724	2.448	4.690	2.966	1.000	3.19E-33
Mean rank		7.733	5.517	4.991	6.681	2.336	4.828	2.836	1.078	N/A
Overall rank		8	6	5	7	2	4	3	1	N/A

**Table 6:** Wilcoxon rank sum test results for ASFOA and competition algorithms based on CEC 2018 test suite.

ASFOA vs. +/=/−		SFOA	LSHADE	MPA	EO	EDO	AE	RIME	ECO	BKA
CEC-2018 test suite	10*D*	28/1/0	26/2/1	26/3/0	29/0/0	27/2/0	28/0/1	23/4/2	25/2/2	24/5/0
	30*D*	29/0/0	24/4/1	29/0/0	28/1/0	29/0/0	27/1/1	27/2/0	29/0/0	28/1/0
	50*D*	29/0/0	22/0/7	29/0/0	29/0/0	29/0/0	25/1/3	25/3/1	29/0/0	29/0/0
	100*D*	29/0/0	17/2/10	29/0/0	27/1/1	29/0/0	20/2/7	23/3/3	29/0/0	29/0/0

**Table 7:** Friedman test results for ASFOA and competition algorithms based on CEC 2018 test suite.

Algorithm	CEC-2018 Test Suite					
	10*D*	30*D*	50*D*	100*D*	Mean Rank	Overall Rank
ASFOA	1.621	1.138	1.483	1.966	1.552	1
SFOA	9.241	9.552	9.552	9.517	9.466	10
LSHADE	4.172	3.552	3.103	2.483	3.328	2
MPA	6.414	7.034	7.103	7.414	6.991	8
EO	6.276	4.897	5.069	5.034	5.319	7
EDO	8.621	9.172	9.310	9.138	9.060	9
AE	5.931	5.069	5.103	4.448	5.138	6
RIME	4.000	4.103	3.931	4.276	4.078	3
ECO	5.379	5.345	4.862	4.931	5.129	5
BKA	3.345	5.138	5.483	5.793	4.940	4
*p*-value	7.12E-29	1.41E-34	3.79E-35	1.17E-34	N/A	N/A

**Table 8:** Ten real-world constrained engineering optimization problems.

Problem	Name	*D*
RW01	Tension/compression spring design problem	3
RW02	Pressure vessel design problem	4
RW03	Three-bar truss design problem	2
RW04	Welded beam design problem	4
RW05	Speed reducer design problem	7
RW06	Gear train design problem	4
RW07	Rolling element bearing design	10
RW08	Cantilever beam design problem	5
RW09	Multiple disk clutch brake design problem	5
RW10	Step-cone pulley problem	5

**Table 9:** Results obtained by ASFOA and other competitors on real-world constrained optimization problems.

Problem ID	Index	ASFOA	SFOA	LSHADE	MPA	EO	EDO	AE	RIME	ECO	BKA
RW1	Best	1.2665E-02	1.2820E-02	1.2716E-02	1.2911E-02	1.2980E-02	1.3089E-02	1.3509E-02	1.3413E-02	1.2919E-02	1.2668E-02
	Mean	1.2702E-02	2.5461E-02	1.3367E-02	1.6123E-02	1.5631E-02	1.5207E+04	1.4847E-02	1.9756E-02	1.4441E-02	1.3159E-02
	Std	8.2232E-05	1.0015E-02	5.7727E-04	3.3888E-03	2.8563E-03	5.7873E+04	9.4987E-04	3.8827E-03	2.7979E-03	6.0885E-04
	Rank	1	9	3	7	6	10	5	8	4	2
RW2	Best	5.8701E+03	8.1947E+03	5.9892E+03	6.4273E+03	7.8141E+03	1.2421E+04	7.6381E+03	6.2728E+03	6.4790E+03	5.8855E+03
	Mean	6.0853E+03	1.2500E+05	6.7584E+03	1.8264E+04	1.1797E+04	3.5599E+04	9.3473E+03	1.0523E+04	3.4927E+04	6.6212E+03
	Std	3.5161E+02	1.1324E+05	5.7082E+02	1.0146E+04	3.1774E+03	1.2315E+04	1.1782E+03	5.6881E+03	6.2321E+04	5.0738E+02
	Rank	1	10	3	7	6	9	4	5	8	2
RW3	Best	2.6389E+02	2.6405E+02	2.6389E+02	2.6389E+02	2.6398E+02	2.6394E+02	2.6390E+02	2.6389E+02	2.6389E+02	2.6389E+02
	Mean	2.6389E+02	2.7014E+02	2.6394E+02	2.6419E+02	2.6455E+02	2.6460E+02	2.6397E+02	2.6498E+02	2.6457E+02	2.6389E+02
	Std	1.1270E-13	5.0704E+00	4.7877E-02	2.7764E-01	9.3304E-01	4.3264E-01	7.1794E-02	1.7793E+00	6.9450E-01	3.9252E-03
	Rank	1	10	3	5	6	8	4	9	7	2
RW4	Best	1.6928E+00	2.0419E+00	1.7171E+00	1.8679E+00	1.7717E+00	2.2015E+00	1.8685E+00	1.7600E+00	1.8056E+00	1.6980E+00
	Mean	1.7097E+00	1.2162E+03	1.7844E+00	2.2860E+00	2.1096E+00	2.5735E+00	1.9736E+00	2.2779E+00	2.3503E+00	1.9099E+00
	Std	4.0994E-02	4.8412E+03	5.9544E-02	2.8505E-01	2.2449E-01	2.3838E-01	8.4918E-02	3.8602E-01	3.2278E-01	3.2853E-01
	Rank	1	10	2	7	5	9	4	6	8	3
RW5	Best	2.9936E+03	3.0802E+03	2.9960E+03	3.0035E+03	3.0064E+03	3.0202E+03	2.8473E+03	3.0003E+03	2.9973E+03	2.9940E+03
	Mean	2.9952E+03	4.2685E+03	3.0010E+03	3.0381E+03	3.0224E+03	3.0437E+03	3.0366E+03	3.0389E+03	3.0170E+03	3.0034E+03
	Std	3.0261E+00	9.0632E+02	3.9677E+00	3.7076E+01	1.1160E+01	1.4414E+01	6.3908E+01	3.0922E+01	9.4244E+00	4.7740E+00
	Rank	1	10	2	7	5	9	6	8	4	3
RW6	Best	2.7009E-12	4.4624E-09	2.7009E-12	1.1661E-10	2.7009E-12	6.6021E-10	2.7009E-12	2.3078E-11	2.3078E-11	2.7009E-12
	Mean	2.9305E-10	2.4683E-06	4.6198E-09	1.0028E-08	1.4663E-08	8.9802E-09	2.2539E-09	7.5928E-09	8.5360E-09	5.5664E-10
	Std	4.6997E-10	6.0246E-06	7.1350E-09	1.0238E-08	2.5060E-08	1.0884E-08	4.9887E-09	8.9848E-09	1.0627E-08	5.9461E-10
	Rank	1	10	4	8	9	7	3	5	6	2
RW7	Best	−2.4358E+05	−2.3707E+05	−2.4358E+05	−2.4358E+05	−2.4358E+05	−2.4358E+05	−7.6151E+04	−2.4357E+05	−2.4358E+05	−2.4358E+05
	Mean	−2.4351E+05	−2.1987E+05	−2.4348E+05	−2.4290E+05	−2.4350E+05	−2.4168E+05	−5.8146E+03	−2.3557E+05	−2.4328E+05	−2.4267E+05
	Std	3.8423E+02	1.2274E+04	1.0869E+02	2.7375E+03	2.0311E+02	2.2804E+03	1.7891E+04	7.9026E+03	1.5422E+03	3.5533E+03
	Rank	1	9	3	5	2	7	10	8	4	6
RW8	Best	1.3400E+00	1.4854E+00	1.3518E+00	1.4527E+00	1.5650E+00	2.2457E+00	1.4251E+00	1.3683E+00	1.3568E+00	1.3411E+00
	Mean	1.3693E+00	3.1499E+00	1.4050E+00	2.4989E+00	1.9993E+00	3.6623E+00	1.5351E+00	1.6227E+00	1.5679E+00	1.4032E+00
	Std	4.7579E-02	1.4021E+00	4.6134E-02	5.4913E-01	2.6884E-01	9.7367E-01	5.6648E-02	2.7138E-01	2.1451E-01	1.0503E-01
	Rank	1	9	3	8	7	10	4	6	5	2
RW9	Best	3.9247E+08	3.9247E+08	3.9247E+08	3.9247E+08	3.9247E+08	3.9247E+08	2.5187E+08	3.9247E+08	3.9247E+08	3.9247E+08
	Mean	3.9247E+08	3.9247E+08	3.9247E+08	3.9247E+08	3.9247E+08	3.9247E+08	2.5233E+08	3.9247E+08	3.9247E+08	3.9247E+08
	Std	1.8187E-07	1.8187E-07	1.8187E-07	1.8187E-07	1.8187E-07	1.8187E-07	1.0054E+06	1.8187E-07	1.8187E-07	1.8187E-07
	Rank	2	2	2	2	2	2	1	2	2	2
RW10	Best	1.6086E+01	9.4388E+01	1.6467E+01	1.7460E+01	1.7464E+01	1.9748E+01	2.3127E+01	1.6518E+01	1.6932E+01	1.6087E+01
	Mean	1.6087E+01	4.8427E+02	1.7736E+01	4.2488E+01	2.3142E+01	7.3404E+01	8.9295E+01	2.1315E+01	3.0227E+01	1.6660E+01
	Std	6.4971E-03	3.0098E+02	8.3231E-01	6.3698E+01	1.1835E+01	1.6369E+02	7.3861E+01	8.9645E+00	3.9361E+01	3.5904E-01
	Rank	1	10	3	7	5	8	9	4	6	2
Friedman ranking		1.500	9.300	3.200	6.700	5.700	8.300	5.000	6.500	5.800	3.000
Wilcoxon rank-sum test		ASFOA vs. +/=/−	9/1/0	9/1/0	9/1/0	9/1/0	9/1/0	9/0/1	9/1/0	9/1/0	8/2/0

## Data Availability

The data are provided within the manuscript.

## Appendix A

**Table A1:** Results obtained by ASFOA and derived algorithms on CEC 2018 of 10D.

No.	Index	SFOA	SFOA-1	SFOA-2	SFOA-3	SFOA-12	SFOA-13	SFOA-23	ASFOA
F1	Best	1.1459E+09	1.3348E+08	5.4107E+02	5.6359E+08	1.0126E+02	1.3997E+08	5.3816E+02	1.0000E+02
	Mean	2.5482E+09	1.0013E+09	1.6880E+09	2.0298E+09	8.3087E+07	9.2358E+08	1.8285E+08	1.2094E+02
	Std	8.9391E+08	7.4447E+08	1.5249E+09	9.4482E+08	1.7645E+08	7.4322E+08	3.8414E+08	7.5259E+01
	Rank	8	5	6	7	2	4	3	1
F2	Best	4.1181E+03	1.7307E+03	3.0000E+02	2.0148E+03	3.0000E+02	1.4481E+03	3.0000E+02	3.0000E+02
	Mean	1.0998E+04	7.4343E+03	2.7719E+03	9.5512E+03	3.0722E+02	6.8666E+03	3.5710E+02	3.0000E+02
	Std	4.5201E+03	3.9013E+03	3.2032E+03	4.8119E+03	2.6126E+01	3.5073E+03	1.2562E+02	1.1071E-13
	Rank	8	6	4	7	2	5	3	1
F3	Best	4.4632E+02	4.2018E+02	4.0467E+02	4.2400E+02	4.0006E+02	4.1079E+02	4.0267E+02	4.0147E+02
	Mean	5.7096E+02	4.9063E+02	5.4368E+02	5.4063E+02	4.1351E+02	4.9978E+02	4.2806E+02	4.0445E+02
	Std	6.0407E+01	4.7150E+01	1.9147E+02	5.3413E+01	1.5977E+01	4.3812E+01	3.3008E+01	1.5531E+00
	Rank	8	4	7	6	2	5	3	1
F4	Best	5.4563E+02	5.3342E+02	5.1054E+02	5.3541E+02	5.0711E+02	5.1080E+02	5.0579E+02	5.0113E+02
	Mean	5.6351E+02	5.5386E+02	5.2727E+02	5.5923E+02	5.1645E+02	5.4423E+02	5.1900E+02	5.0888E+02
	Std	8.1003E+00	1.0293E+01	1.2145E+01	1.1440E+01	8.7298E+00	1.4460E+01	8.0029E+00	4.7143E+00
	Rank	8	6	4	7	2	5	3	1
F5	Best	6.2764E+02	6.1681E+02	6.0022E+02	6.1665E+02	6.0005E+02	6.1841E+02	6.0005E+02	6.0000E+02
	Mean	6.3962E+02	6.3363E+02	6.1565E+02	6.3596E+02	6.0503E+02	6.3302E+02	6.0541E+02	6.0008E+02
	Std	8.6715E+00	7.8302E+00	8.2229E+00	8.8124E+00	5.5545E+00	7.5232E+00	5.9260E+00	3.2027E-01
	Rank	8	6	4	7	2	5	3	1
F6	Best	7.8537E+02	7.4234E+02	7.2947E+02	7.8548E+02	7.1614E+02	7.5290E+02	7.1670E+02	7.1309E+02
	Mean	8.5008E+02	8.2037E+02	7.7517E+02	8.3271E+02	7.2927E+02	8.0804E+02	7.4328E+02	7.2049E+02
	Std	2.8201E+01	3.2761E+01	2.9537E+01	3.3218E+01	1.0763E+01	2.9336E+01	2.1164E+01	6.5643E+00
	Rank	8	6	4	7	2	5	3	1
F7	Best	8.3862E+02	8.3251E+02	8.0684E+02	8.3223E+02	8.0497E+02	8.2131E+02	8.0399E+02	8.0497E+02
	Mean	8.6268E+02	8.5891E+02	8.2794E+02	8.5754E+02	8.1523E+02	8.4981E+02	8.1851E+02	8.1141E+02
	Std	1.0428E+01	1.3482E+01	1.2134E+01	1.3946E+01	7.2536E+00	1.6037E+01	7.3218E+00	5.7764E+00
	Rank	8	7	4	6	2	5	3	1
F8	Best	1.1834E+03	1.0766E+03	9.0086E+02	1.0904E+03	9.0000E+02	1.0420E+03	9.0001E+02	9.0000E+02
	Mean	1.7254E+03	1.5854E+03	1.0589E+03	1.6896E+03	9.1520E+02	1.5370E+03	9.4483E+02	9.0037E+02
	Std	3.4645E+02	4.1828E+02	1.8612E+02	4.0727E+02	2.1355E+01	4.5052E+02	7.8294E+01	9.8049E-01
	Rank	8	6	4	7	2	5	3	1
F9	Best	1.8057E+03	1.6908E+03	1.3078E+03	1.7421E+03	1.3642E+03	1.7955E+03	1.2857E+03	1.1408E+03
	Mean	2.3483E+03	2.2604E+03	2.0139E+03	2.3051E+03	1.8636E+03	2.2424E+03	1.9512E+03	1.7111E+03
	Std	2.8407E+02	2.5899E+02	3.6451E+02	2.3691E+02	3.0546E+02	2.8718E+02	3.3833E+02	3.1320E+02
	Rank	8	6	4	7	2	5	3	1
F10	Best	1.2578E+03	1.1576E+03	1.1069E+03	1.2464E+03	1.1051E+03	1.1680E+03	1.1040E+03	1.1008E+03
	Mean	1.6612E+03	1.4346E+03	1.6232E+03	1.6315E+03	1.1271E+03	1.4380E+03	1.1392E+03	1.1057E+03
	Std	2.6349E+02	1.7419E+02	1.2255E+03	2.4369E+02	2.2811E+01	2.3589E+02	7.5946E+01	3.5232E+00
	Rank	8	4	6	7	2	5	3	1
F11	Best	2.2736E+06	8.5350E+04	1.4044E+03	5.1136E+05	1.2009E+03	6.3994E+05	1.2106E+03	1.2000E+03
	Mean	5.8247E+07	1.3039E+07	1.4716E+07	2.7974E+07	2.5424E+03	1.1590E+07	1.3625E+06	1.3816E+03
	Std	4.0723E+07	1.1961E+07	2.0904E+07	2.9200E+07	2.1307E+03	1.2377E+07	6.3811E+06	1.5236E+02
	Rank	8	5	6	7	2	4	3	1
F12	Best	1.4773E+04	3.3314E+03	1.3081E+03	3.2794E+03	1.3086E+03	9.3504E+03	1.3102E+03	1.3041E+03
	Mean	4.2214E+05	7.3943E+04	5.7391E+03	1.1807E+05	1.4411E+03	7.0678E+04	2.1131E+03	1.3140E+03
	Std	6.4680E+05	1.0129E+05	6.6826E+03	1.4066E+05	3.0524E+02	1.9540E+05	2.5200E+03	8.8401E+00
	Rank	8	6	4	7	2	5	3	1
F13	Best	1.5199E+03	1.4864E+03	1.4105E+03	1.4683E+03	1.4169E+03	1.5665E+03	1.4163E+03	1.4066E+03
	Mean	4.2235E+03	3.1563E+03	1.5559E+03	4.0495E+03	1.4266E+03	3.7976E+03	1.4306E+03	1.4203E+03
	Std	4.0619E+03	4.9987E+03	5.1292E+02	2.9990E+03	3.6084E+00	4.2954E+03	1.7471E+01	4.2389E+00
	Rank	8	5	4	7	2	6	3	1
F14	Best	2.2323E+03	1.7394E+03	1.5045E+03	1.8095E+03	1.5032E+03	1.9729E+03	1.5038E+03	1.5023E+03
	Mean	1.3572E+04	1.2063E+04	3.3624E+03	1.9512E+04	1.5115E+03	1.4370E+04	1.5220E+03	1.5042E+03
	Std	1.1988E+04	8.0804E+03	7.4827E+03	1.7997E+04	1.2354E+01	1.1113E+04	7.0320E+01	2.6355E+00
	Rank	6	5	4	8	2	7	3	1
F15	Best	1.6699E+03	1.6596E+03	1.6023E+03	1.6493E+03	1.6033E+03	1.6394E+03	1.6047E+03	1.6005E+03
	Mean	1.8892E+03	1.8882E+03	1.7928E+03	1.8233E+03	1.7320E+03	1.8073E+03	1.6816E+03	1.6486E+03
	Std	9.8735E+01	1.2987E+02	1.6746E+02	7.7882E+01	1.3000E+02	1.0343E+02	7.8317E+01	8.1537E+01
	Rank	8	7	4	6	3	5	2	1
F16	Best	1.7736E+03	1.7652E+03	1.7352E+03	1.7631E+03	1.7308E+03	1.7587E+03	1.7228E+03	1.7227E+03
	Mean	1.8689E+03	1.8492E+03	1.8172E+03	1.8350E+03	1.7749E+03	1.8284E+03	1.7661E+03	1.7463E+03
	Std	6.7151E+01	5.8931E+01	9.2672E+01	4.7003E+01	4.3118E+01	5.1921E+01	3.2527E+01	2.8140E+01
	Rank	8	7	4	6	3	5	2	1
F17	Best	3.7866E+04	7.0534E+03	1.8178E+03	2.7468E+04	1.8125E+03	1.6503E+04	1.8115E+03	1.8011E+03
	Mean	4.6676E+05	1.8841E+05	4.3494E+04	3.8756E+05	1.8411E+03	1.0151E+05	2.4891E+03	1.8177E+03
	Std	5.6187E+05	1.6886E+05	2.0074E+05	5.0917E+05	3.3464E+01	9.1048E+04	3.5896E+03	7.2711E+00
	Rank	8	6	4	7	2	5	3	1
F18	Best	1.9804E+03	2.4156E+03	1.9039E+03	2.6823E+03	1.9036E+03	2.5465E+03	1.9039E+03	1.9016E+03
	Mean	2.4548E+04	1.6095E+04	4.0669E+03	2.6368E+04	1.9064E+03	2.1527E+04	2.1122E+03	1.9037E+03
	Std	1.9037E+04	1.3280E+04	5.2502E+03	2.7010E+04	3.6739E+00	1.4745E+04	1.1274E+03	9.8836E-01
	Rank	7	5	4	8	2	6	3	1
F19	Best	2.0863E+03	2.0830E+03	2.0245E+03	2.0650E+03	2.0363E+03	2.0383E+03	2.0258E+03	2.0133E+03
	Mean	2.1805E+03	2.1754E+03	2.1318E+03	2.1716E+03	2.1086E+03	2.1611E+03	2.0807E+03	2.0584E+03
	Std	7.5474E+01	6.1676E+01	8.0992E+01	6.9841E+01	6.5988E+01	5.9634E+01	6.3674E+01	3.7106E+01
	Rank	8	7	4	6	3	5	2	1
F20	Best	2.2166E+03	2.2062E+03	2.2026E+03	2.2134E+03	2.2002E+03	2.2101E+03	2.2000E+03	2.2000E+03
	Mean	2.2954E+03	2.2784E+03	2.2929E+03	2.2830E+03	2.2811E+03	2.2628E+03	2.2768E+03	2.2599E+03
	Std	6.5901E+01	6.7289E+01	5.0938E+01	6.6767E+01	5.5446E+01	6.7542E+01	4.9798E+01	5.6292E+01
	Rank	8	4	7	6	5	2	3	1
F21	Best	2.3373E+03	2.2497E+03	2.2593E+03	2.2857E+03	2.2342E+03	2.2671E+03	2.2619E+03	2.2458E+03
	Mean	2.5360E+03	2.4162E+03	2.4504E+03	2.5145E+03	2.3157E+03	2.4059E+03	2.3586E+03	2.2998E+03
	Std	1.1180E+02	7.6116E+01	1.4142E+02	1.1805E+02	1.9617E+01	7.2381E+01	1.0184E+02	1.0295E+01
	Rank	8	5	6	7	2	4	3	1
F22	Best	2.6426E+03	2.6273E+03	2.6084E+03	2.6372E+03	2.6052E+03	2.6297E+03	2.6111E+03	2.6000E+03
	Mean	2.6564E+03	2.6476E+03	2.6411E+03	2.6546E+03	2.6211E+03	2.6465E+03	2.6294E+03	2.6158E+03
	Std	7.1629E+00	1.1268E+01	2.5796E+01	8.3104E+00	1.0588E+01	1.0285E+01	1.3688E+01	7.8317E+00
	Rank	8	6	4	7	2	5	3	1
F23	Best	2.6320E+03	2.5739E+03	2.6002E+03	2.6122E+03	2.5272E+03	2.5616E+03	2.5418E+03	2.5000E+03
	Mean	2.7760E+03	2.7557E+03	2.7456E+03	2.7676E+03	2.7132E+03	2.7580E+03	2.7348E+03	2.7218E+03
	Std	4.0479E+01	6.8290E+01	5.8057E+01	4.2412E+01	8.2717E+01	5.6780E+01	5.7576E+01	6.5603E+01
	Rank	8	5	4	7	1	6	3	2
F24	Best	2.9808E+03	2.9307E+03	2.9043E+03	2.9651E+03	2.8978E+03	2.9161E+03	2.8986E+03	2.8977E+03
	Mean	3.0424E+03	2.9910E+03	3.0197E+03	3.0462E+03	2.9347E+03	3.0053E+03	2.9452E+03	2.9198E+03
	Std	3.4495E+01	3.7654E+01	9.6693E+01	5.3003E+01	2.9115E+01	4.6215E+01	2.6348E+01	2.3226E+01
	Rank	7	4	6	8	2	5	3	1
F25	Best	3.1038E+03	2.9872E+03	2.9002E+03	3.0405E+03	2.9000E+03	2.9551E+03	2.9001E+03	2.9000E+03
	Mean	3.2527E+03	3.1583E+03	3.1988E+03	3.2309E+03	3.1259E+03	3.1131E+03	3.0126E+03	2.9453E+03
	Std	9.7136E+01	1.2383E+02	2.0886E+02	1.0243E+02	2.4989E+02	8.8797E+01	1.2600E+02	1.8904E+02
	Rank	8	5	6	7	4	3	2	1
F26	Best	3.1013E+03	3.0973E+03	3.0898E+03	3.0960E+03	3.0908E+03	3.0941E+03	3.0891E+03	3.0890E+03
	Mean	3.1057E+03	3.1056E+03	3.1145E+03	3.1034E+03	3.1008E+03	3.1017E+03	3.0996E+03	3.0954E+03
	Std	2.0629E+00	1.6014E+01	2.4390E+01	2.5522E+00	6.9218E+00	2.7617E+00	1.4563E+01	5.7849E+00
	Rank	7	6	8	5	3	4	2	1
F27	Best	3.2879E+03	3.2062E+03	3.1799E+03	3.2550E+03	3.1002E+03	3.1968E+03	3.1006E+03	3.1000E+03
	Mean	3.3809E+03	3.3395E+03	3.3856E+03	3.3659E+03	3.2737E+03	3.3569E+03	3.3221E+03	3.2429E+03
	Std	5.5347E+01	7.9362E+01	1.2143E+02	6.6338E+01	1.1514E+02	7.4798E+01	1.2854E+02	1.2358E+02
	Rank	7	4	8	6	2	5	3	1
F28	Best	3.2088E+03	3.1545E+03	3.1546E+03	3.1849E+03	3.1448E+03	3.1802E+03	3.1539E+03	3.1362E+03
	Mean	3.2876E+03	3.2884E+03	3.2326E+03	3.2809E+03	3.2070E+03	3.2736E+03	3.1987E+03	3.1722E+03
	Std	5.5842E+01	7.6299E+01	5.7943E+01	5.9732E+01	5.0016E+01	5.7940E+01	4.4788E+01	3.8889E+01
	Rank	7	8	4	6	3	5	2	1
F29	Best	2.1833E+05	1.4938E+05	3.4239E+03	1.3352E+05	3.5646E+03	2.0144E+04	3.4127E+03	3.3953E+03
	Mean	1.4143E+06	1.6106E+06	1.3471E+06	1.3102E+06	1.2763E+06	1.4109E+06	1.5290E+06	1.7467E+06
	Std	1.2559E+06	1.3785E+06	1.6046E+06	1.0941E+06	2.1682E+06	1.1944E+06	2.5210E+06	2.9411E+06
	Rank	5	7	3	2	1	4	6	8

**Table A2:** Results obtained by ASFOA and derived algorithms on CEC 2018 of 30D.

No.	Index	SFOA	SFOA-1	SFOA-2	SFOA-3	SFOA-12	SFOA-13	SFOA-23	ASFOA
F1	Best	2.3062E+10	1.1332E+10	3.7143E+09	2.6346E+10	1.3527E+08	1.0286E+10	7.2295E+06	8.7715E+03
	Mean	4.0583E+10	2.0742E+10	2.3856E+10	3.9614E+10	6.0979E+09	2.0736E+10	1.0756E+10	1.2953E+08
	Std	9.0925E+09	5.1434E+09	8.9528E+09	8.3731E+09	3.5896E+09	6.0672E+09	1.0273E+10	1.3463E+08
	Rank	8	5	6	7	2	4	3	1
F2	Best	7.6789E+04	6.5651E+04	4.9905E+02	6.7309E+04	3.7679E+02	4.2892E+04	3.7272E+02	3.0008E+02
	Mean	1.4961E+05	1.3169E+05	1.1367E+04	1.3345E+05	3.1150E+03	1.1119E+05	3.5286E+03	9.2575E+02
	Std	3.4071E+04	3.2708E+04	2.0055E+04	3.5688E+04	2.5840E+03	3.2355E+04	4.2932E+03	9.1157E+02
	Rank	8	6	4	7	2	5	3	1
F3	Best	3.1490E+03	1.4675E+03	1.0619E+03	2.7560E+03	6.0168E+02	1.4545E+03	5.3734E+02	4.7146E+02
	Mean	5.6440E+03	3.0650E+03	4.7720E+03	4.9031E+03	9.7236E+02	3.2226E+03	1.2254E+03	5.3925E+02
	Std	2.0115E+03	1.0375E+03	2.6823E+03	1.8556E+03	3.5880E+02	1.2155E+03	9.4281E+02	5.4055E+01
	Rank	8	4	6	7	2	5	3	1
F4	Best	7.5829E+02	7.6133E+02	5.9031E+02	7.7331E+02	5.6116E+02	7.2494E+02	5.4869E+02	5.3003E+02
	Mean	8.9229E+02	8.6380E+02	6.8371E+02	8.6903E+02	6.0796E+02	8.2015E+02	6.3354E+02	5.5436E+02
	Std	5.6233E+01	4.2196E+01	6.6195E+01	4.2877E+01	2.7649E+01	4.8724E+01	4.7238E+01	1.5198E+01
	Rank	8	6	4	7	2	5	3	1
F5	Best	6.5179E+02	6.4876E+02	6.1965E+02	6.5092E+02	6.1935E+02	6.5379E+02	6.1088E+02	6.0058E+02
	Mean	6.8059E+02	6.7631E+02	6.4310E+02	6.7527E+02	6.3032E+02	6.6905E+02	6.2288E+02	6.0945E+02
	Std	1.1313E+01	1.5805E+01	1.3859E+01	1.2804E+01	6.7148E+00	9.1620E+00	7.2091E+00	6.7964E+00
	Rank	8	7	4	6	3	5	2	1
F6	Best	1.2703E+03	1.1647E+03	9.3447E+02	1.2869E+03	8.0523E+02	1.0801E+03	8.0921E+02	7.7428E+02
	Mean	1.4370E+03	1.3114E+03	1.2494E+03	1.3961E+03	9.3679E+02	1.2793E+03	1.0206E+03	8.3649E+02
	Std	7.5722E+01	7.4528E+01	1.2064E+02	6.7706E+01	5.4807E+01	9.4493E+01	1.1957E+02	3.5786E+01
	Rank	8	6	4	7	2	5	3	1
F7	Best	1.0535E+03	1.0377E+03	8.5618E+02	9.9993E+02	8.4511E+02	9.9616E+02	8.5274E+02	8.2781E+02
	Mean	1.1578E+03	1.1384E+03	9.8965E+02	1.1337E+03	8.9484E+02	1.1149E+03	9.0731E+02	8.5352E+02
	Std	4.1707E+01	4.4203E+01	6.2546E+01	4.6896E+01	2.1516E+01	4.6840E+01	3.3569E+01	1.5237E+01
	Rank	8	7	4	6	2	5	3	1
F8	Best	7.9784E+03	5.0528E+03	1.4939E+03	6.2679E+03	1.2695E+03	6.9102E+03	9.6777E+02	9.0151E+02
	Mean	1.5035E+04	1.3520E+04	4.5809E+03	1.3224E+04	2.4192E+03	1.2186E+04	2.2239E+03	1.0122E+03
	Std	3.9334E+03	3.7953E+03	2.8109E+03	3.3600E+03	8.5455E+02	3.0768E+03	1.1344E+03	1.2474E+02
	Rank	8	7	4	6	3	5	2	1
F9	Best	6.7987E+03	6.2435E+03	3.6712E+03	7.1389E+03	4.2757E+03	6.0311E+03	4.3080E+03	2.6977E+03
	Mean	8.0903E+03	7.7218E+03	6.2513E+03	7.8578E+03	5.3626E+03	7.6344E+03	6.5034E+03	4.9296E+03
	Std	6.0038E+02	5.2151E+02	1.2329E+03	3.9466E+02	8.5405E+02	6.1688E+02	7.8391E+02	8.7884E+02
	Rank	8	6	3	7	2	5	4	1
F10	Best	3.9174E+03	2.0439E+03	1.1631E+03	3.0786E+03	1.1605E+03	2.4947E+03	1.1463E+03	1.1243E+03
	Mean	7.5730E+03	5.0852E+03	2.3243E+03	7.6999E+03	1.2767E+03	4.9885E+03	1.2453E+03	1.1579E+03
	Std	1.6913E+03	1.8377E+03	1.8247E+03	2.7222E+03	6.8924E+01	1.8129E+03	6.8373E+01	2.8265E+01
	Rank	7	6	4	8	3	5	2	1
F11	Best	1.8942E+09	7.3362E+08	2.3661E+04	1.7418E+09	4.0802E+03	4.8251E+08	6.9321E+03	1.5079E+03
	Mean	4.3124E+09	1.5744E+09	3.0626E+09	3.7358E+09	9.9156E+07	1.8218E+09	5.2492E+08	1.5591E+04
	Std	1.3274E+09	7.7061E+08	2.6668E+09	1.4249E+09	1.5654E+08	1.0778E+09	1.0770E+09	5.9738E+04
	Rank	8	4	6	7	2	5	3	1
F12	Best	5.8001E+08	6.6147E+07	2.4283E+03	3.4049E+08	1.9827E+03	2.7991E+07	2.0295E+03	1.4355E+03
	Mean	2.2868E+09	5.1938E+08	9.3887E+08	1.4248E+09	2.9097E+06	2.5417E+08	3.4122E+07	2.4167E+03
	Std	1.0420E+09	4.4648E+08	1.1507E+09	8.8317E+08	1.5887E+07	1.9317E+08	7.4521E+07	9.3027E+02
	Rank	8	5	6	7	2	4	3	1
F13	Best	1.6309E+05	4.5849E+04	1.4737E+03	1.3307E+05	1.4673E+03	2.9327E+04	1.4791E+03	1.4595E+03
	Mean	9.0457E+05	7.4134E+05	4.2486E+04	6.9203E+05	1.5048E+03	5.9363E+05	1.4965E+03	1.4741E+03
	Std	7.3331E+05	6.9612E+05	1.7087E+05	4.4168E+05	2.7998E+01	6.0676E+05	1.0767E+01	1.1964E+01
	Rank	8	7	4	6	3	5	2	1
F14	Best	8.6793E+06	7.3667E+05	1.6683E+03	5.4169E+06	1.6292E+03	8.2041E+05	1.6095E+03	1.5469E+03
	Mean	1.8412E+08	2.0095E+07	8.7484E+06	1.3051E+08	2.2528E+03	2.3353E+07	6.6859E+05	1.6323E+03
	Std	1.5659E+08	3.1722E+07	1.9182E+07	1.1838E+08	1.4058E+03	4.7923E+07	3.2255E+06	5.7809E+01
	Rank	8	5	4	7	2	6	3	1
F15	Best	3.4950E+03	3.1851E+03	2.2617E+03	3.5497E+03	2.2460E+03	2.8886E+03	2.0663E+03	1.7415E+03
	Mean	4.1139E+03	3.8613E+03	2.7948E+03	4.0304E+03	2.7055E+03	3.7361E+03	2.6369E+03	2.3225E+03
	Std	3.2679E+02	3.5293E+02	3.8217E+02	2.4831E+02	2.9583E+02	4.1050E+02	3.0971E+02	2.7402E+02
	Rank	8	6	4	7	3	5	2	1
F16	Best	2.2715E+03	2.3483E+03	1.8255E+03	2.3899E+03	1.8147E+03	2.2781E+03	1.8207E+03	1.7459E+03
	Mean	3.0158E+03	2.9316E+03	2.3755E+03	2.8507E+03	2.1444E+03	2.7162E+03	2.1529E+03	1.9583E+03
	Std	2.9685E+02	2.6779E+02	2.8997E+02	2.7930E+02	2.0315E+02	2.8821E+02	2.5691E+02	1.8630E+02
	Rank	8	7	4	6	2	5	3	1
F17	Best	1.7934E+06	1.4119E+06	1.8590E+03	3.2830E+06	1.8600E+03	6.7779E+05	1.8675E+03	1.8336E+03
	Mean	1.4446E+07	8.4917E+06	5.1785E+06	1.2587E+07	1.9920E+03	8.0244E+06	5.3506E+05	1.8583E+03
	Std	1.1927E+07	7.6543E+06	1.3648E+07	6.9163E+06	1.0429E+02	6.9179E+06	2.0389E+06	1.8105E+01
	Rank	8	6	4	7	2	5	3	1
F18	Best	2.5945E+07	6.7003E+06	1.9834E+03	1.4427E+07	1.9398E+03	2.2360E+06	1.9384E+03	1.9256E+03
	Mean	3.4420E+08	3.9773E+07	4.0303E+07	2.5558E+08	2.1619E+03	4.0446E+07	4.9114E+05	1.9352E+03
	Std	2.6808E+08	4.4814E+07	5.7113E+07	2.2908E+08	5.2124E+02	4.9721E+07	1.6668E+06	4.9904E+00
	Rank	8	4	5	7	2	6	3	1
F19	Best	2.5312E+03	2.5581E+03	2.1108E+03	2.4150E+03	2.2150E+03	2.3966E+03	2.2015E+03	2.0587E+03
	Mean	2.7960E+03	2.8447E+03	2.5914E+03	2.8310E+03	2.5767E+03	2.7792E+03	2.5139E+03	2.3943E+03
	Std	1.3038E+02	1.5667E+02	2.2999E+02	1.7145E+02	2.2121E+02	1.8847E+02	1.8909E+02	2.1490E+02
	Rank	6	8	4	7	3	5	2	1
F20	Best	2.5942E+03	2.5357E+03	2.3487E+03	2.5622E+03	2.3645E+03	2.5351E+03	2.3668E+03	2.3271E+03
	Mean	2.6481E+03	2.6051E+03	2.4614E+03	2.6245E+03	2.4071E+03	2.5973E+03	2.4161E+03	2.3597E+03
	Std	3.2907E+01	3.8252E+01	7.7012E+01	3.2602E+01	2.9149E+01	3.1460E+01	3.0232E+01	1.5430E+01
	Rank	8	6	4	7	2	5	3	1
F21	Best	4.6053E+03	3.5872E+03	4.0909E+03	4.3958E+03	2.8327E+03	3.3027E+03	2.5208E+03	2.3282E+03
	Mean	8.4972E+03	7.6504E+03	6.8620E+03	8.2869E+03	4.3821E+03	7.6585E+03	5.7025E+03	3.7336E+03
	Std	1.6511E+03	2.2168E+03	1.5038E+03	1.6055E+03	1.5769E+03	2.1636E+03	1.8743E+03	2.0511E+03
	Rank	8	5	4	7	2	6	3	1
F22	Best	2.9548E+03	2.9032E+03	2.7803E+03	2.9334E+03	2.7031E+03	2.8722E+03	2.7156E+03	2.6984E+03
	Mean	3.0096E+03	2.9846E+03	2.9618E+03	3.0076E+03	2.8815E+03	2.9725E+03	2.8476E+03	2.7355E+03
	Std	2.8092E+01	3.7646E+01	9.1541E+01	3.2850E+01	9.4904E+01	4.6576E+01	8.2119E+01	2.6554E+01
	Rank	8	6	4	7	3	5	2	1
F23	Best	3.0882E+03	3.0718E+03	2.9658E+03	3.0582E+03	2.9340E+03	3.0613E+03	2.8766E+03	2.8563E+03
	Mean	3.1340E+03	3.1235E+03	3.1504E+03	3.1203E+03	3.0704E+03	3.1178E+03	3.0234E+03	2.9139E+03
	Std	2.4826E+01	2.9097E+01	1.0205E+02	2.7499E+01	7.9248E+01	3.1077E+01	8.6692E+01	4.2684E+01
	Rank	7	6	8	5	3	4	2	1
F24	Best	3.9130E+03	3.2875E+03	3.1865E+03	3.6699E+03	2.9872E+03	3.3173E+03	2.9144E+03	2.8989E+03
	Mean	5.0242E+03	3.8828E+03	3.9545E+03	4.9633E+03	3.1187E+03	3.8896E+03	3.2368E+03	2.9273E+03
	Std	7.1347E+02	4.0867E+02	6.3131E+02	5.0159E+02	9.3023E+01	3.0310E+02	2.7505E+02	2.5989E+01
	Rank	8	4	6	7	2	5	3	1
F25	Best	7.1398E+03	6.6554E+03	5.1262E+03	6.9929E+03	4.2164E+03	6.3003E+03	3.8880E+03	3.2477E+03
	Mean	8.0251E+03	7.7021E+03	6.8750E+03	7.8678E+03	5.7609E+03	7.4803E+03	5.2333E+03	4.3298E+03
	Std	5.1601E+02	6.1563E+02	1.0745E+03	6.9437E+02	7.5582E+02	5.4748E+02	6.6299E+02	3.6463E+02
	Rank	8	6	4	7	3	5	2	1
F26	Best	3.3213E+03	3.3087E+03	3.2383E+03	3.3268E+03	3.2404E+03	3.2744E+03	3.1880E+03	3.1906E+03
	Mean	3.4372E+03	3.4105E+03	3.5078E+03	3.4080E+03	3.3666E+03	3.3853E+03	3.3044E+03	3.2469E+03
	Std	6.5346E+01	5.0443E+01	1.9151E+02	5.7879E+01	1.0883E+02	6.5141E+01	7.7998E+01	4.4932E+01
	Rank	7	6	8	5	3	4	2	1
F27	Best	4.6022E+03	3.9526E+03	3.4201E+03	4.2384E+03	3.3443E+03	4.0551E+03	3.2312E+03	3.2115E+03
	Mean	5.7794E+03	4.6941E+03	4.9612E+03	5.7335E+03	3.8030E+03	4.7481E+03	3.7020E+03	3.2638E+03
	Std	6.2047E+02	4.7693E+02	8.3169E+02	6.7247E+02	3.6111E+02	5.5623E+02	5.3675E+02	3.6083E+01
	Rank	8	4	6	7	3	5	2	1
F28	Best	4.6879E+03	4.4359E+03	3.5981E+03	4.6662E+03	3.5398E+03	4.2560E+03	3.3440E+03	3.3869E+03
	Mean	5.2452E+03	5.0608E+03	4.4744E+03	5.1875E+03	4.0161E+03	4.9291E+03	3.9223E+03	3.6442E+03
	Std	3.2416E+02	3.8601E+02	6.1060E+02	2.6852E+02	3.0297E+02	2.9903E+02	3.1189E+02	1.5722E+02
	Rank	8	6	4	7	3	5	2	1
F29	Best	5.7196E+07	1.6955E+07	9.0756E+03	2.0386E+07	6.2369E+03	1.2247E+07	6.5995E+03	5.1136E+03
	Mean	2.8815E+08	6.4837E+07	8.1146E+07	1.9853E+08	2.8432E+04	7.5541E+07	3.7099E+06	6.4971E+03
	Std	1.4218E+08	4.1090E+07	9.0817E+07	1.1972E+08	5.6500E+04	6.2724E+07	1.3281E+07	1.4613E+03
	Rank	8	4	6	7	2	5	3	1

**Table A3:** Results obtained by ASFOA and derived algorithms on CEC 2018 of 50D.

No.	Index	SFOA	SFOA-1	SFOA-2	SFOA-3	SFOA-12	SFOA-13	SFOA-23	ASFOA
F1	Best	6.2605E+10	3.7383E+10	4.1882E+10	6.8361E+10	1.0670E+10	3.7833E+10	7.9222E+09	1.2014E+09
	Mean	8.9943E+10	5.4110E+10	6.5968E+10	8.6665E+10	2.4609E+10	5.5498E+10	3.4090E+10	3.6468E+09
	Std	1.0922E+10	9.9319E+09	1.1625E+10	1.0174E+10	9.0513E+09	7.6444E+09	1.9890E+10	1.9007E+09
	Rank	8	4	6	7	2	5	3	1
F2	Best	1.7917E+05	1.7117E+05	6.8082E+03	1.4649E+05	4.5598E+03	1.4530E+05	1.0994E+04	4.1412E+03
	Mean	3.0206E+05	2.6205E+05	3.0560E+04	2.6527E+05	1.6837E+04	2.4961E+05	2.2392E+04	1.2049E+04
	Std	6.1305E+04	4.5029E+04	1.7558E+04	5.3559E+04	6.9977E+03	4.5998E+04	9.9979E+03	6.0465E+03
	Rank	8	6	4	7	2	5	3	1
F3	Best	9.7232E+03	5.4805E+03	6.9767E+03	1.4349E+04	1.5839E+03	7.5234E+03	8.9312E+02	6.3479E+02
	Mean	2.1569E+04	1.1145E+04	1.4977E+04	2.2154E+04	3.5093E+03	1.2628E+04	4.8022E+03	8.6764E+02
	Std	6.1640E+03	3.2256E+03	4.9234E+03	5.5580E+03	1.5897E+03	2.6456E+03	2.8442E+03	1.4890E+02
	Rank	7	4	6	8	2	5	3	1
F4	Best	1.1379E+03	1.0486E+03	6.9536E+02	1.0584E+03	6.6819E+02	1.0101E+03	6.2703E+02	5.9620E+02
	Mean	1.2332E+03	1.1939E+03	9.2659E+02	1.1875E+03	7.2843E+02	1.1344E+03	7.5058E+02	6.4324E+02
	Std	6.1630E+01	7.6222E+01	1.0167E+02	7.9834E+01	3.2866E+01	6.9874E+01	7.7408E+01	2.9718E+01
	Rank	8	7	4	6	2	5	3	1
F5	Best	6.7198E+02	6.7811E+02	6.4206E+02	6.6865E+02	6.2783E+02	6.7113E+02	6.1718E+02	6.0970E+02
	Mean	6.9689E+02	6.9583E+02	6.6220E+02	6.9576E+02	6.4044E+02	6.8883E+02	6.3743E+02	6.1875E+02
	Std	1.1551E+01	9.2229E+00	9.5341E+00	1.0565E+01	5.4849E+00	9.1111E+00	1.0224E+01	5.3238E+00
	Rank	8	7	4	6	3	5	2	1
F6	Best	1.8791E+03	1.7221E+03	1.3780E+03	1.7856E+03	1.0701E+03	1.6190E+03	1.0506E+03	9.0910E+02
	Mean	2.0549E+03	1.8889E+03	1.7449E+03	1.9631E+03	1.2181E+03	1.8495E+03	1.3597E+03	1.0026E+03
	Std	1.0393E+02	9.0064E+01	1.6698E+02	9.6678E+01	7.6138E+01	1.0542E+02	2.0484E+02	6.1592E+01
	Rank	8	6	4	7	2	5	3	1
F7	Best	1.3530E+03	1.3416E+03	1.0416E+03	1.3911E+03	9.5665E+02	1.3057E+03	9.8342E+02	9.0567E+02
	Mean	1.5359E+03	1.4947E+03	1.2592E+03	1.5024E+03	1.0304E+03	1.4339E+03	1.0797E+03	9.4550E+02
	Std	8.6487E+01	6.6768E+01	1.3325E+02	5.0975E+01	4.3878E+01	7.0786E+01	7.5927E+01	3.0032E+01
	Rank	8	6	4	7	2	5	3	1
F8	Best	2.7063E+04	2.3448E+04	8.6183E+03	2.0752E+04	5.9913E+03	1.7459E+04	2.2886E+03	1.1600E+03
	Mean	4.4370E+04	4.1145E+04	1.7660E+04	4.0880E+04	9.4509E+03	3.8479E+04	7.6985E+03	3.0973E+03
	Std	9.1525E+03	9.1650E+03	6.1511E+03	1.0053E+04	2.2586E+03	8.9767E+03	4.2051E+03	1.2012E+03
	Rank	8	7	4	6	3	5	2	1
F9	Best	1.2839E+04	1.2678E+04	6.5160E+03	1.3095E+04	6.0579E+03	1.0273E+04	7.7825E+03	5.9846E+03
	Mean	1.4400E+04	1.3911E+04	9.7133E+03	1.4016E+04	9.2408E+03	1.3737E+04	1.1510E+04	8.4901E+03
	Std	5.5837E+02	4.9623E+02	1.5743E+03	6.5229E+02	1.7241E+03	9.3029E+02	1.5991E+03	1.3167E+03
	Rank	8	6	3	7	2	5	4	1
F10	Best	1.2768E+04	4.7724E+03	1.3186E+03	4.3739E+03	1.3071E+03	5.6452E+03	1.2876E+03	1.2022E+03
	Mean	1.7665E+04	1.3164E+04	6.8745E+03	1.4906E+04	1.6579E+03	1.1949E+04	1.9775E+03	1.3071E+03
	Std	3.1293E+03	4.2430E+03	5.1973E+03	4.1780E+03	2.9972E+02	4.0368E+03	2.5773E+03	5.9800E+01
	Rank	8	6	4	7	2	5	3	1
F11	Best	1.3864E+10	4.6634E+09	6.6536E+09	9.3733E+09	1.5848E+07	3.0968E+09	7.0008E+05	1.1225E+04
	Mean	2.7590E+10	1.3070E+10	2.6430E+10	2.3475E+10	2.2447E+09	1.3563E+10	6.0549E+09	5.4765E+06
	Std	7.5838E+09	4.3632E+09	1.0221E+10	6.6948E+09	2.4040E+09	5.5953E+09	1.1372E+10	1.5034E+07
	Rank	8	4	7	6	2	5	3	1
F12	Best	3.2125E+09	3.3726E+08	1.4482E+04	1.2385E+09	4.6026E+03	5.3249E+08	6.3153E+03	2.4749E+03
	Mean	9.0579E+09	2.8780E+09	5.1317E+09	7.7891E+09	1.5302E+08	2.5813E+09	3.5863E+08	5.0669E+03
	Std	2.9781E+09	1.8294E+09	2.9677E+09	3.2272E+09	3.9584E+08	1.6262E+09	1.0802E+09	1.5213E+03
	Rank	8	5	6	7	2	4	3	1
F13	Best	1.2021E+06	9.6042E+05	1.5534E+03	1.1149E+06	1.5638E+03	3.9999E+05	1.5539E+03	1.5367E+03
	Mean	5.2809E+06	5.2044E+06	1.2494E+06	5.2909E+06	1.6304E+03	4.6874E+06	4.8737E+03	1.5727E+03
	Std	2.7323E+06	3.1857E+06	2.6216E+06	2.6567E+06	5.0677E+01	2.8220E+06	1.7846E+04	2.7398E+01
	Rank	7	6	4	8	2	5	3	1
F14	Best	8.6568E+08	7.5715E+07	2.3006E+03	5.6437E+08	1.9636E+03	3.4231E+07	1.8834E+03	1.7308E+03
	Mean	2.8958E+09	6.1491E+08	8.0393E+08	2.3711E+09	4.2987E+03	4.3148E+08	1.8937E+07	1.9395E+03
	Std	1.2947E+09	5.1109E+08	1.0268E+09	1.2349E+09	6.6637E+03	3.5968E+08	5.5362E+07	1.5412E+02
	Rank	8	5	6	7	2	4	3	1
F15	Best	5.5715E+03	4.9009E+03	3.1202E+03	5.2220E+03	2.7340E+03	4.1959E+03	2.5663E+03	2.0975E+03
	Mean	6.5505E+03	6.0054E+03	4.1928E+03	5.9604E+03	3.3511E+03	5.7489E+03	3.4441E+03	2.7883E+03
	Std	4.1820E+02	5.1566E+02	8.0775E+02	3.8284E+02	4.6434E+02	6.4186E+02	5.9090E+02	3.4178E+02
	Rank	8	7	4	6	2	5	3	1
F16	Best	4.3054E+03	4.1854E+03	2.5480E+03	4.1951E+03	2.3818E+03	3.4812E+03	2.3442E+03	2.1025E+03
	Mean	6.2455E+03	4.8494E+03	3.6173E+03	5.8733E+03	2.9236E+03	4.6375E+03	3.0534E+03	2.5931E+03
	Std	1.2959E+03	4.8934E+02	6.5180E+02	8.8413E+02	3.4954E+02	5.0829E+02	3.3432E+02	2.7915E+02
	Rank	8	6	4	7	2	5	3	1
F17	Best	9.0326E+06	7.8518E+06	5.7065E+03	5.1332E+06	2.0308E+03	5.9151E+06	2.0110E+03	1.9165E+03
	Mean	4.9978E+07	3.8542E+07	1.2433E+07	3.3863E+07	4.0506E+03	3.5863E+07	1.6912E+04	2.0246E+03
	Std	2.5767E+07	2.4130E+07	1.8184E+07	2.7024E+07	3.5842E+03	1.9772E+07	4.9350E+04	9.1449E+01
	Rank	8	7	4	5	2	6	3	1
F18	Best	3.2114E+08	1.9486E+07	2.1637E+03	2.1079E+08	1.9676E+03	1.3505E+07	2.0174E+03	1.9660E+03
	Mean	1.3203E+09	1.9870E+08	4.4626E+08	8.2552E+08	2.2471E+04	1.3521E+08	4.3300E+07	1.9973E+03
	Std	6.5718E+08	1.6465E+08	4.6109E+08	4.8280E+08	8.3730E+04	1.0855E+08	1.8531E+08	2.3899E+01
	Rank	8	5	6	7	2	4	3	1
F19	Best	3.5041E+03	3.2644E+03	2.4763E+03	3.5051E+03	2.3452E+03	3.3529E+03	2.3852E+03	2.3302E+03
	Mean	3.9761E+03	3.8751E+03	3.0945E+03	3.9790E+03	2.8458E+03	3.8321E+03	3.1177E+03	2.8724E+03
	Std	2.9686E+02	2.4829E+02	3.0855E+02	2.2398E+02	3.4967E+02	2.5703E+02	3.0086E+02	3.0268E+02
	Rank	7	6	3	8	1	5	4	2
F20	Best	2.8959E+03	2.7929E+03	2.4925E+03	2.8642E+03	2.4287E+03	2.8141E+03	2.4726E+03	2.4013E+03
	Mean	3.0457E+03	3.0024E+03	2.7383E+03	3.0190E+03	2.5522E+03	2.9530E+03	2.5708E+03	2.4473E+03
	Std	7.2876E+01	9.0723E+01	1.0740E+02	6.4693E+01	6.3834E+01	6.7954E+01	6.6623E+01	2.8049E+01
	Rank	8	6	4	7	2	5	3	1
F21	Best	1.4456E+04	1.3980E+04	7.9005E+03	1.3854E+04	8.1193E+03	1.3337E+04	9.5064E+03	7.1652E+03
	Mean	1.5929E+04	1.5484E+04	1.1993E+04	1.5622E+04	1.0641E+04	1.5151E+04	1.2418E+04	1.0052E+04
	Std	6.2138E+02	5.5819E+02	2.3284E+03	6.4571E+02	1.6608E+03	9.1199E+02	1.7662E+03	1.2379E+03
	Rank	8	6	3	7	2	5	4	1
F22	Best	3.3763E+03	3.3497E+03	3.1860E+03	3.3634E+03	3.0145E+03	3.2633E+03	2.9364E+03	2.8319E+03
	Mean	3.5202E+03	3.4790E+03	3.5338E+03	3.4945E+03	3.2411E+03	3.4590E+03	3.1889E+03	2.9610E+03
	Std	6.7160E+01	6.8616E+01	2.2325E+02	4.9183E+01	1.5106E+02	8.7204E+01	1.2409E+02	6.1996E+01
	Rank	7	5	8	6	3	4	2	1
F23	Best	3.5232E+03	3.4473E+03	3.3814E+03	3.4656E+03	3.2434E+03	3.4243E+03	3.2641E+03	3.0360E+03
	Mean	3.5827E+03	3.5389E+03	3.7280E+03	3.5611E+03	3.4773E+03	3.5335E+03	3.4542E+03	3.1612E+03
	Std	3.8643E+01	5.0694E+01	2.2864E+02	4.6767E+01	1.6793E+02	3.9679E+01	1.3985E+02	6.9973E+01
	Rank	7	5	8	6	3	4	2	1
F24	Best	9.9035E+03	6.5639E+03	4.4963E+03	9.7054E+03	3.6192E+03	6.3585E+03	3.1936E+03	3.0845E+03
	Mean	1.3998E+04	8.7312E+03	9.5314E+03	1.2737E+04	4.8320E+03	9.0186E+03	5.7065E+03	3.2972E+03
	Std	1.8987E+03	1.0845E+03	2.6087E+03	1.4076E+03	7.7187E+02	1.3141E+03	2.1337E+03	1.1791E+02
	Rank	8	4	6	7	2	5	3	1
F25	Best	1.1218E+04	9.7724E+03	9.3706E+03	1.0460E+04	6.6549E+03	1.0163E+04	6.5217E+03	4.9980E+03
	Mean	1.2648E+04	1.2324E+04	1.3127E+04	1.2285E+04	9.0728E+03	1.1990E+04	8.8479E+03	6.1490E+03
	Std	7.0377E+02	1.3942E+03	2.2163E+03	1.1052E+03	1.2702E+03	7.8685E+02	1.4631E+03	6.5485E+02
	Rank	7	6	8	5	3	4	2	1
F26	Best	4.0294E+03	3.7885E+03	3.3352E+03	3.8616E+03	3.5246E+03	3.7349E+03	3.3896E+03	3.2767E+03
	Mean	4.3115E+03	4.1968E+03	4.5113E+03	4.2570E+03	4.0213E+03	4.1132E+03	3.7754E+03	3.6322E+03
	Std	1.6948E+02	2.3173E+02	4.9649E+02	1.9586E+02	3.4176E+02	1.9840E+02	1.9972E+02	2.0819E+02
	Rank	7	5	8	6	3	4	2	1
F27	Best	8.6529E+03	6.7553E+03	6.5179E+03	8.1070E+03	4.4186E+03	6.3506E+03	3.5646E+03	3.3601E+03
	Mean	1.0964E+04	7.8591E+03	8.8980E+03	1.0393E+04	5.6011E+03	8.0263E+03	5.8958E+03	3.6330E+03
	Std	1.3244E+03	6.7141E+02	1.2935E+03	9.6691E+02	5.7735E+02	8.8784E+02	1.5713E+03	2.6263E+02
	Rank	8	4	6	7	2	5	3	1
F28	Best	7.9662E+03	6.4942E+03	4.6348E+03	7.6367E+03	3.8778E+03	6.2878E+03	3.6940E+03	3.5158E+03
	Mean	1.0372E+04	8.4590E+03	7.6303E+03	9.4283E+03	5.4058E+03	8.7496E+03	4.8057E+03	3.9012E+03
	Std	1.3260E+03	8.7777E+02	2.5954E+03	1.2320E+03	1.1299E+03	1.5288E+03	9.2396E+02	2.4946E+02
	Rank	8	5	4	7	3	6	2	1
F29	Best	8.4153E+08	2.8786E+08	1.6765E+07	3.1837E+08	1.9613E+06	2.7312E+08	2.6784E+06	9.2487E+05
	Mean	2.0408E+09	7.2200E+08	1.0122E+09	1.5699E+09	1.8770E+07	7.3635E+08	1.4048E+08	2.0087E+06
	Std	7.5488E+08	3.9560E+08	9.2999E+08	6.0839E+08	4.7994E+07	4.7001E+08	4.2246E+08	6.1840E+05
	Rank	8	4	6	7	2	5	3	1

**Table A4:** Results obtained by ASFOA and derived algorithms on CEC 2018 of 100D.

No.	Index	SFOA	SFOA-1	SFOA-2	SFOA-3	SFOA-12	SFOA-13	SFOA-23	ASFOA
F1	Best	1.9678E+11	1.2571E+11	1.1268E+11	1.8734E+11	6.0088E+10	1.3392E+11	4.7374E+10	1.8656E+10
	Mean	2.2991E+11	1.6910E+11	1.8338E+11	2.2331E+11	8.6059E+10	1.6305E+11	1.3791E+11	3.1238E+10
	Std	1.6906E+10	1.7934E+10	2.5483E+10	1.8643E+10	1.3156E+10	1.0788E+10	4.4304E+10	7.4148E+09
	Rank	8	5	6	7	2	4	3	1
F2	Best	4.9257E+05	5.1812E+05	4.6512E+04	5.5178E+05	6.4062E+04	5.3841E+05	7.4289E+04	4.6400E+04
	Mean	7.4518E+05	6.6881E+05	1.0337E+05	7.3305E+05	8.7122E+04	6.8886E+05	1.3120E+05	7.0330E+04
	Std	1.0978E+05	1.0118E+05	3.1915E+04	9.0475E+04	1.5845E+04	7.4593E+04	3.2640E+04	1.2969E+04
	Rank	8	5	3	7	2	6	4	1
F3	Best	4.0945E+04	2.3548E+04	1.1852E+04	5.1289E+04	6.5433E+03	2.1130E+04	6.2060E+03	2.1365E+03
	Mean	7.0085E+04	3.2604E+04	4.7788E+04	6.8371E+04	1.0816E+04	3.5353E+04	2.2196E+04	3.3471E+03
	Std	1.2625E+04	5.7928E+03	1.5150E+04	8.2239E+03	2.5641E+03	7.3465E+03	1.0654E+04	9.3507E+02
	Rank	8	4	6	7	2	5	3	1
F4	Best	1.9988E+03	1.8836E+03	1.1833E+03	1.8822E+03	1.0590E+03	1.8231E+03	1.0342E+03	8.9943E+02
	Mean	2.1487E+03	2.0776E+03	1.5861E+03	2.1065E+03	1.1967E+03	2.0111E+03	1.3088E+03	9.9622E+02
	Std	1.0326E+02	1.0659E+02	1.9093E+02	1.3527E+02	7.3000E+01	1.2066E+02	1.8661E+02	5.9191E+01
	Rank	8	6	4	7	2	5	3	1
F5	Best	7.0476E+02	7.0409E+02	6.4392E+02	7.0113E+02	6.4286E+02	6.8194E+02	6.4264E+02	6.2464E+02
	Mean	7.2200E+02	7.1819E+02	6.6908E+02	7.1521E+02	6.5274E+02	7.0880E+02	6.5223E+02	6.3347E+02
	Std	1.0532E+01	9.3223E+00	9.3194E+00	8.4305E+00	3.8329E+00	1.5305E+01	7.4261E+00	4.9829E+00
	Rank	8	7	4	6	3	5	2	1
F6	Best	3.5922E+03	3.3823E+03	2.7202E+03	3.5355E+03	1.9971E+03	3.1538E+03	1.8517E+03	1.4159E+03
	Mean	3.9403E+03	3.6757E+03	3.4709E+03	3.8520E+03	2.2846E+03	3.6630E+03	2.9192E+03	1.7219E+03
	Std	1.5200E+02	1.5022E+02	3.1314E+02	1.6484E+02	1.9173E+02	1.8571E+02	4.5168E+02	1.3583E+02
	Rank	8	6	4	7	2	5	3	1
F7	Best	2.4347E+03	2.2304E+03	1.6560E+03	2.3205E+03	1.4206E+03	2.1542E+03	1.3398E+03	1.1970E+03
	Mean	2.5900E+03	2.4516E+03	2.0210E+03	2.5122E+03	1.5595E+03	2.4023E+03	1.6141E+03	1.3092E+03
	Std	1.0929E+02	1.1436E+02	2.1018E+02	1.0047E+02	7.4509E+01	1.3572E+02	1.9202E+02	5.7236E+01
	Rank	8	6	4	7	2	5	3	1
F8	Best	6.8194E+04	7.1459E+04	2.7652E+04	7.0944E+04	2.2420E+04	6.6772E+04	1.5891E+04	7.9993E+03
	Mean	1.1544E+05	1.1833E+05	5.4879E+04	1.1069E+05	3.4233E+04	1.0754E+05	3.2400E+04	1.6693E+04
	Std	2.9601E+04	2.4741E+04	1.7900E+04	3.1140E+04	5.7666E+03	3.0076E+04	1.1439E+04	5.0865E+03
	Rank	7	8	4	6	3	5	2	1
F9	Best	2.9316E+04	2.8795E+04	1.6214E+04	2.9148E+04	1.5416E+04	2.7478E+04	1.8372E+04	1.4099E+04
	Mean	3.1001E+04	3.0364E+04	2.1429E+04	3.0827E+04	2.0080E+04	3.0370E+04	2.3174E+04	1.8575E+04
	Std	8.0885E+02	8.9567E+02	3.0971E+03	6.8805E+02	3.2324E+03	9.4118E+02	3.0283E+03	2.2514E+03
	Rank	8	5	3	7	2	6	4	1
F10	Best	1.1902E+05	7.3929E+04	3.3289E+03	1.0051E+05	2.8667E+03	7.7694E+04	2.9228E+03	2.0433E+03
	Mean	2.0638E+05	1.3608E+05	1.9259E+04	1.6499E+05	4.2501E+03	1.2442E+05	4.0497E+03	2.7581E+03
	Std	4.6062E+04	3.7968E+04	2.9406E+04	3.4062E+04	9.5283E+02	3.4390E+04	8.5796E+02	3.0281E+02
	Rank	8	6	4	7	3	5	2	1
F11	Best	9.1249E+10	4.2651E+10	1.9707E+10	8.4295E+10	4.1939E+09	4.8159E+10	1.7805E+09	3.4767E+07
	Mean	1.2639E+11	6.3927E+10	8.3388E+10	1.2520E+11	1.9361E+10	7.2009E+10	3.1044E+10	8.7817E+08
	Std	1.9121E+10	1.0503E+10	2.6986E+10	1.9487E+10	9.6554E+09	1.3895E+10	2.2486E+10	7.0848E+08
	Rank	8	4	6	7	2	5	3	1
F12	Best	1.1654E+10	7.1379E+09	5.0326E+04	9.2069E+09	2.9650E+04	5.3423E+09	3.6917E+04	7.1107E+03
	Mean	2.4452E+10	1.1500E+10	1.7927E+10	2.3855E+10	1.2565E+09	1.1562E+10	3.3895E+09	1.3052E+04
	Std	6.4288E+09	3.6150E+09	7.2414E+09	5.6638E+09	1.4262E+09	4.1050E+09	4.3972E+09	3.9578E+03
	Rank	8	4	6	7	2	5	3	1
F13	Best	2.1113E+07	1.6810E+07	1.9567E+03	2.4313E+07	1.8573E+03	7.6261E+06	1.9019E+03	1.7604E+03
	Mean	6.1706E+07	4.1855E+07	1.9435E+07	5.1367E+07	2.0695E+03	3.6323E+07	1.8141E+06	1.8259E+03
	Std	2.4854E+07	1.8542E+07	2.0252E+07	2.1312E+07	1.4595E+02	1.9663E+07	6.8648E+06	3.9498E+01
	Rank	8	6	4	7	2	5	3	1
F14	Best	3.4866E+09	6.8316E+08	7.8270E+03	2.5618E+09	3.0548E+03	1.7055E+08	4.5102E+03	2.0140E+03
	Mean	8.6943E+09	2.7372E+09	5.5432E+09	6.9839E+09	2.3196E+07	2.3358E+09	6.5030E+08	2.4686E+03
	Std	2.3791E+09	1.3851E+09	4.0148E+09	2.8221E+09	4.6075E+07	1.2322E+09	1.2356E+09	3.3476E+02
	Rank	8	5	6	7	2	4	3	1
F15	Best	1.2340E+04	1.1020E+04	5.5952E+03	1.2060E+04	4.6491E+03	9.3278E+03	4.4307E+03	3.1097E+03
	Mean	1.4401E+04	1.3551E+04	1.0093E+04	1.3896E+04	6.1019E+03	1.2663E+04	6.3753E+03	4.8073E+03
	Std	1.3565E+03	1.5987E+03	2.9759E+03	1.0992E+03	8.1299E+02	1.1343E+03	1.0100E+03	8.3085E+02
	Rank	8	6	4	7	2	5	3	1
F16	Best	1.4705E+04	8.9105E+03	4.1143E+03	1.1362E+04	3.1802E+03	8.4590E+03	2.9351E+03	2.9288E+03
	Mean	7.4825E+04	2.5030E+04	8.4380E+04	5.9283E+04	4.3867E+03	2.3857E+04	5.4546E+03	4.0847E+03
	Std	6.4345E+04	1.7514E+04	1.2738E+05	4.8369E+04	7.3460E+02	3.8168E+04	1.8202E+03	4.7091E+02
	Rank	7	5	8	6	2	4	3	1
F17	Best	2.7518E+07	1.4083E+07	3.1149E+03	3.0085E+07	2.6581E+03	1.7162E+07	2.9699E+03	2.1844E+03
	Mean	9.6594E+07	5.4592E+07	1.9855E+07	7.6321E+07	1.2994E+04	4.5856E+07	1.9800E+05	2.3936E+03
	Std	4.1099E+07	2.0365E+07	3.4283E+07	2.8876E+07	1.4389E+04	2.0156E+07	9.8298E+05	1.8990E+02
	Rank	8	6	4	7	2	5	3	1
F18	Best	3.8417E+09	5.1506E+08	3.5654E+04	1.7052E+09	2.2831E+03	1.8556E+08	3.1454E+03	2.0863E+03
	Mean	9.3343E+09	2.3825E+09	5.7050E+09	7.7003E+09	7.8941E+07	2.8109E+09	5.9200E+08	2.2280E+03
	Std	3.1914E+09	1.2435E+09	4.5826E+09	3.2587E+09	1.9647E+08	1.6683E+09	1.1305E+09	9.4775E+01
	Rank	8	4	6	7	2	5	3	1
F19	Best	6.2739E+03	6.2728E+03	4.0486E+03	6.2115E+03	3.6264E+03	5.5633E+03	3.6102E+03	3.0980E+03
	Mean	7.3320E+03	7.1964E+03	5.0387E+03	7.2927E+03	4.5921E+03	7.1648E+03	5.1865E+03	4.5095E+03
	Std	4.6963E+02	3.9379E+02	6.9936E+02	4.9235E+02	5.3039E+02	5.6122E+02	6.6095E+02	5.5392E+02
	Rank	8	6	3	7	2	5	4	1
F20	Best	4.0296E+03	3.7351E+03	3.2126E+03	4.0326E+03	3.0351E+03	3.8720E+03	2.9816E+03	2.7606E+03
	Mean	4.2323E+03	4.0955E+03	3.7381E+03	4.1798E+03	3.2739E+03	4.0913E+03	3.1945E+03	2.8846E+03
	Std	1.2648E+02	1.2682E+02	2.8911E+02	9.6855E+01	1.6667E+02	1.4747E+02	1.4309E+02	7.1527E+01
	Rank	8	6	4	7	3	5	2	1
F21	Best	3.1092E+04	2.9433E+04	1.8592E+04	3.0495E+04	1.7163E+04	3.0225E+04	1.9950E+04	1.6813E+04
	Mean	3.2953E+04	3.2259E+04	2.5912E+04	3.2496E+04	2.2881E+04	3.1911E+04	2.6262E+04	2.0884E+04
	Std	7.4832E+02	1.0562E+03	3.9972E+03	7.7691E+02	3.0400E+03	1.0076E+03	3.1334E+03	2.1981E+03
	Rank	8	6	3	7	2	5	4	1
F22	Best	4.3098E+03	4.2545E+03	4.0204E+03	4.2972E+03	3.9084E+03	4.3113E+03	3.8340E+03	3.5001E+03
	Mean	4.5918E+03	4.4569E+03	4.7038E+03	4.5301E+03	4.3085E+03	4.4829E+03	4.1710E+03	3.7487E+03
	Std	1.0128E+02	1.0059E+02	4.6535E+02	1.0206E+02	1.8212E+02	9.2533E+01	2.1492E+02	1.6862E+02
	Rank	7	4	8	6	3	5	2	1
F23	Best	5.2053E+03	5.1493E+03	4.8558E+03	5.2302E+03	4.7596E+03	5.0305E+03	4.7970E+03	4.1806E+03
	Mean	5.5709E+03	5.4461E+03	7.1635E+03	5.5025E+03	5.7784E+03	5.4387E+03	5.6832E+03	4.6037E+03
	Std	1.6942E+02	1.5581E+02	1.4424E+03	1.4070E+02	5.1758E+02	1.8828E+02	6.0525E+02	2.6655E+02
	Rank	5	3	8	4	7	2	6	1
F24	Best	1.9394E+04	1.2124E+04	1.0759E+04	1.9609E+04	5.7260E+03	1.3206E+04	5.9521E+03	4.2124E+03
	Mean	2.5889E+04	1.7062E+04	1.9441E+04	2.4050E+04	8.7357E+03	1.6448E+04	1.3180E+04	5.0318E+03
	Std	3.2108E+03	2.4394E+03	3.9547E+03	2.7246E+03	1.5423E+03	1.8912E+03	5.0212E+03	4.7829E+02
	Rank	8	5	6	7	2	4	3	1
F25	Best	2.6709E+04	2.4457E+04	2.1184E+04	2.6635E+04	1.9780E+04	2.5421E+04	1.5324E+04	1.1921E+04
	Mean	3.0351E+04	2.9974E+04	3.6654E+04	3.0946E+04	2.7400E+04	2.8390E+04	2.5303E+04	1.6267E+04
	Std	1.9096E+03	3.6654E+03	6.7477E+03	3.6915E+03	3.1768E+03	1.6232E+03	5.6632E+03	2.4070E+03
	Rank	6	5	8	7	3	4	2	1
F26	Best	5.4857E+03	4.8743E+03	3.8785E+03	5.4084E+03	3.7357E+03	5.1149E+03	3.7898E+03	3.4635E+03
	Mean	6.2147E+03	5.8702E+03	6.6020E+03	6.1187E+03	4.9686E+03	5.6340E+03	4.5083E+03	3.6228E+03
	Std	4.9885E+02	4.3014E+02	1.2819E+03	4.1971E+02	8.5275E+02	3.2454E+02	5.9691E+02	1.5747E+02
	Rank	7	5	8	6	3	4	2	1
F27	Best	2.2163E+04	1.5989E+04	9.4755E+03	2.4320E+04	9.8945E+03	1.7961E+04	6.3143E+03	4.2261E+03
	Mean	3.1044E+04	2.1691E+04	2.4991E+04	2.9395E+04	1.3296E+04	2.2663E+04	1.5079E+04	5.9712E+03
	Std	3.5389E+03	2.1864E+03	4.1572E+03	2.7313E+03	2.0706E+03	2.3600E+03	5.0371E+03	8.6085E+02
	Rank	8	4	6	7	2	5	3	1
F28	Best	3.5826E+04	1.3760E+04	7.8932E+03	1.9161E+04	6.9353E+03	1.4746E+04	6.1759E+03	4.8569E+03
	Mean	6.9377E+04	2.1878E+04	2.5338E+04	4.2038E+04	9.1186E+03	2.1167E+04	8.9574E+03	6.2462E+03
	Std	2.6102E+04	7.2692E+03	2.0783E+04	1.7447E+04	1.5726E+03	4.8577E+03	2.6940E+03	6.8596E+02
	Rank	8	5	6	7	3	4	2	1
F29	Best	6.3811E+09	1.9787E+09	4.1077E+05	3.9192E+09	7.2814E+05	2.2741E+09	4.0355E+05	2.2672E+04
	Mean	1.4101E+10	7.6273E+09	1.2859E+10	1.2975E+10	3.9008E+08	6.0400E+09	9.5397E+08	3.2283E+05
	Std	3.2728E+09	3.0629E+09	7.9116E+09	4.0472E+09	6.5774E+08	2.6166E+09	1.5768E+09	4.6528E+05
	Rank	8	5	6	7	2	4	3	1

**Table A5:** Results obtained by ASFOA and comparison algorithms on CEC 2018 of 10D.

No.	Index	ASFOA	SFOA	LSHADE	MPA	EO	EDO	AE	RIME	ECO	BKA
F1	Best	1.0000E+02	1.1459E+09	9.2141E+05	5.5089E+07	5.1789E+06	4.4490E+08	5.4945E+06	1.3468E+06	6.7686E+05	2.1191E+04
	Mean	1.2094E+02	2.5482E+09	1.4991E+07	5.0427E+08	3.9452E+07	2.1779E+09	1.5297E+07	7.7616E+06	1.1421E+07	2.2304E+06
	Std	7.5259E+01	8.9391E+08	1.3915E+07	3.7389E+08	2.9120E+07	7.7982E+08	5.6372E+06	5.9240E+06	1.5834E+07	5.6751E+06
	Rank	1	10	5	8	7	9	6	3	4	2
F2	Best	3.0000E+02	4.1181E+03	3.6279E+02	2.4532E+03	9.3939E+02	3.5642E+03	3.0863E+03	5.4643E+02	3.3940E+02	3.0781E+02
	Mean	3.0000E+02	1.0998E+04	8.0355E+02	6.8505E+03	8.7875E+03	7.5039E+03	6.7453E+03	2.6710E+03	1.9301E+03	5.4841E+02
	Std	1.1071E-13	4.5201E+03	3.9527E+02	2.9816E+03	4.9446E+03	1.6918E+03	2.0326E+03	1.5613E+03	1.4405E+03	2.7826E+02
	Rank	1	10	3	7	9	8	6	5	4	2
F3	Best	4.0147E+02	4.4632E+02	4.0463E+02	4.1456E+02	4.0625E+02	4.9690E+02	4.0702E+02	4.0240E+02	4.0379E+02	4.0034E+02
	Mean	4.0445E+02	5.7096E+02	4.0835E+02	4.5556E+02	4.2106E+02	6.0050E+02	4.0831E+02	4.2434E+02	4.1431E+02	4.1200E+02
	Std	1.5531E+00	6.0407E+01	1.5465E+00	2.5032E+01	1.7362E+01	5.4906E+01	7.2240E-01	2.8416E+01	1.5507E+01	1.2546E+01
	Rank	1	9	3	8	6	10	2	7	5	4
F4	Best	5.0113E+02	5.4563E+02	5.3158E+02	5.1886E+02	5.0565E+02	5.5872E+02	5.2352E+02	5.0915E+02	5.1326E+02	5.0711E+02
	Mean	5.0888E+02	5.6351E+02	5.4694E+02	5.4160E+02	5.3160E+02	5.7357E+02	5.3894E+02	5.2143E+02	5.3404E+02	5.2875E+02
	Std	4.7143E+00	8.1003E+00	8.1228E+00	1.3126E+01	7.8707E+00	6.8097E+00	7.3690E+00	5.6836E+00	1.3624E+01	1.3228E+01
	Rank	1	9	8	7	4	10	6	2	5	3
F5	Best	6.0000E+02	6.2764E+02	6.0198E+02	6.0914E+02	6.0211E+02	6.2515E+02	6.0251E+02	6.0069E+02	6.0439E+02	6.0970E+02
	Mean	6.0008E+02	6.3962E+02	6.0605E+02	6.2090E+02	6.0573E+02	6.3595E+02	6.0377E+02	6.0317E+02	6.1915E+02	6.2189E+02
	Std	3.2027E-01	8.6715E+00	2.3934E+00	6.4799E+00	1.8023E+00	5.0359E+00	7.7464E-01	1.6411E+00	7.2374E+00	7.6354E+00
	Rank	1	10	5	7	4	9	3	2	6	8
F6	Best	7.1309E+02	7.8537E+02	7.4023E+02	7.4668E+02	7.3529E+02	7.7282E+02	7.3295E+02	7.2977E+02	7.2882E+02	7.1903E+02
	Mean	7.2049E+02	8.5008E+02	7.6579E+02	7.7063E+02	7.4718E+02	7.8890E+02	7.4780E+02	7.4793E+02	7.5778E+02	7.4287E+02
	Std	6.5643E+00	2.8201E+01	1.0433E+01	1.4923E+01	6.6230E+00	9.7970E+00	6.1197E+00	1.0695E+01	1.4747E+01	1.1751E+01
	Rank	1	10	7	8	3	9	4	5	6	2
F7	Best	8.0497E+02	8.3862E+02	8.3163E+02	8.1723E+02	8.1381E+02	8.3362E+02	8.1985E+02	8.1110E+02	8.1332E+02	8.0996E+02
	Mean	8.1141E+02	8.6268E+02	8.4719E+02	8.4016E+02	8.2995E+02	8.5379E+02	8.3709E+02	8.2596E+02	8.3052E+02	8.2213E+02
	Std	5.7764E+00	1.0428E+01	7.1494E+00	1.0824E+01	7.6935E+00	9.4988E+00	5.7131E+00	1.0149E+01	1.0960E+01	8.9578E+00
	Rank	1	10	8	7	4	9	6	3	5	2
F8	Best	9.0000E+02	1.1834E+03	9.0278E+02	9.6984E+02	9.0531E+02	1.0647E+03	9.0235E+02	9.0133E+02	9.0522E+02	9.1798E+02
	Mean	9.0037E+02	1.7254E+03	9.2815E+02	1.1551E+03	9.3378E+02	1.2943E+03	9.0591E+02	9.2918E+02	1.1229E+03	1.0293E+03
	Std	9.8049E-01	3.4645E+02	1.3974E+01	1.5508E+02	2.6288E+01	1.2722E+02	2.8397E+00	3.5241E+01	1.8433E+02	6.6202E+01
	Rank	1	10	3	8	5	9	2	4	7	6
F9	Best	1.1408E+03	1.8057E+03	2.2041E+03	1.3062E+03	1.8922E+03	1.8887E+03	2.3126E+03	1.1851E+03	1.5418E+03	1.4755E+03
	Mean	1.7111E+03	2.3483E+03	2.6859E+03	2.1716E+03	2.3819E+03	2.3606E+03	2.7076E+03	1.7703E+03	2.0843E+03	1.8627E+03
	Std	3.1320E+02	2.8407E+02	2.2805E+02	3.0035E+02	2.4185E+02	2.2735E+02	1.5718E+02	2.8760E+02	2.3566E+02	2.1520E+02
	Rank	1	6	9	5	8	7	10	2	4	3
F10	Best	1.1008E+03	1.2578E+03	1.1177E+03	1.1676E+03	1.1182E+03	1.2739E+03	1.1380E+03	1.1106E+03	1.1335E+03	1.1096E+03
	Mean	1.1057E+03	1.6612E+03	1.1325E+03	1.2596E+03	1.1685E+03	1.4916E+03	1.1608E+03	1.1496E+03	1.2192E+03	1.1741E+03
	Std	3.5232E+00	2.6349E+02	1.0909E+01	7.5300E+01	4.4999E+01	1.3195E+02	1.4284E+01	5.1048E+01	8.8313E+01	8.3135E+01
	Rank	1	10	2	8	5	9	4	3	7	6
F11	Best	1.2000E+03	2.2736E+06	1.1964E+04	5.6491E+04	7.9145E+04	8.1940E+06	4.3862E+05	1.5117E+05	4.8873E+03	2.9758E+03
	Mean	1.3816E+03	5.8247E+07	4.2844E+05	4.6518E+06	2.6077E+06	4.0189E+07	1.3862E+06	3.5658E+06	3.2962E+06	4.3098E+04
	Std	1.5236E+02	4.0723E+07	4.6510E+05	5.3045E+06	3.9533E+06	2.1686E+07	8.2291E+05	3.2568E+06	6.2147E+06	1.5618E+05
	Rank	1	10	3	8	5	9	4	7	6	2
F12	Best	1.3041E+03	1.4773E+04	1.6149E+03	2.1332E+03	1.9138E+03	9.6545E+03	4.6609E+03	2.2922E+03	3.2564E+03	1.4848E+03
	Mean	1.3140E+03	4.2214E+05	2.3114E+03	1.5197E+04	1.5319E+04	6.5485E+04	1.0012E+04	1.3344E+04	1.4220E+04	2.5682E+03
	Std	8.8401E+00	6.4680E+05	4.9312E+02	1.2970E+04	1.3549E+04	6.3013E+04	4.2014E+03	1.1446E+04	9.8130E+03	1.3407E+03
	Rank	1	10	2	7	8	9	4	5	6	3
F13	Best	1.4066E+03	1.5199E+03	1.4362E+03	1.4574E+03	1.5103E+03	1.4946E+03	1.5464E+03	1.4677E+03	1.4490E+03	1.4288E+03
	Mean	1.4203E+03	4.2235E+03	1.4496E+03	1.5526E+03	3.7008E+03	1.6291E+03	1.7540E+03	5.4663E+03	1.5183E+03	1.4677E+03
	Std	4.2389E+00	4.0619E+03	1.0038E+01	6.2976E+01	2.3977E+03	9.0457E+01	2.9037E+02	5.2046E+03	4.6839E+01	3.7679E+01
	Rank	1	9	2	5	8	6	7	10	4	3
F14	Best	1.5023E+03	2.2323E+03	1.5217E+03	1.6726E+03	1.8848E+03	1.7105E+03	1.7645E+03	1.6028E+03	1.5404E+03	1.5117E+03
	Mean	1.5042E+03	1.3572E+04	1.5804E+03	2.6180E+03	1.0966E+04	2.9202E+03	3.8605E+03	6.5886E+03	2.0010E+03	1.6506E+03
	Std	2.6355E+00	1.1988E+04	2.9469E+01	1.0606E+03	1.3610E+04	9.1626E+02	1.7687E+03	6.5794E+03	7.9545E+02	9.2568E+01
	Rank	1	10	2	5	9	6	7	8	4	3
F15	Best	1.6005E+03	1.6699E+03	1.6120E+03	1.6325E+03	1.6181E+03	1.7450E+03	1.6534E+03	1.6061E+03	1.6305E+03	1.6143E+03
	Mean	1.6486E+03	1.8892E+03	1.7151E+03	1.8164E+03	1.7221E+03	1.9041E+03	1.8237E+03	1.7425E+03	1.8236E+03	1.7429E+03
	Std	8.1537E+01	9.8735E+01	6.4210E+01	1.1789E+02	7.3519E+01	8.4824E+01	8.4610E+01	1.1795E+02	1.1218E+02	1.0292E+02
	Rank	1	9	2	6	3	10	8	4	7	5
F16	Best	1.7227E+03	1.7736E+03	1.7496E+03	1.7319E+03	1.7452E+03	1.7855E+03	1.7659E+03	1.7242E+03	1.7286E+03	1.7239E+03
	Mean	1.7463E+03	1.8689E+03	1.7978E+03	1.7867E+03	1.7821E+03	1.8357E+03	1.8083E+03	1.7640E+03	1.7831E+03	1.7607E+03
	Std	2.8140E+01	6.7151E+01	2.7294E+01	3.4252E+01	2.0083E+01	2.8567E+01	1.9828E+01	3.7911E+01	3.0507E+01	2.6895E+01
	Rank	1	10	7	6	4	9	8	3	5	2
F17	Best	1.8011E+03	3.7866E+04	1.9834E+03	2.8745E+03	8.0827E+03	1.6721E+04	5.1948E+03	4.5963E+03	2.1863E+03	1.8475E+03
	Mean	1.8177E+03	4.6676E+05	3.2020E+03	2.1688E+04	3.2610E+04	3.6949E+05	2.3894E+04	2.0117E+04	2.3841E+04	2.6162E+03
	Std	7.2711E+00	5.6187E+05	1.3066E+03	1.3831E+04	1.8013E+04	3.0529E+05	1.4488E+04	1.0091E+04	1.3341E+04	1.9317E+03
	Rank	1	10	3	5	8	9	7	4	6	2
F18	Best	1.9016E+03	1.9804E+03	1.9107E+03	1.9923E+03	2.2014E+03	2.1916E+03	2.1301E+03	1.9262E+03	1.9290E+03	1.9056E+03
	Mean	1.9037E+03	2.4548E+04	1.9330E+03	5.7999E+03	1.0638E+04	8.4244E+03	4.1904E+03	7.2163E+03	4.7279E+03	1.9473E+03
	Std	9.8836E-01	1.9037E+04	1.6915E+01	5.1685E+03	9.1341E+03	5.6183E+03	1.5697E+03	6.8378E+03	6.2138E+03	3.5284E+01
	Rank	1	10	2	6	9	8	4	7	5	3
F19	Best	2.0133E+03	2.0863E+03	2.0458E+03	2.0372E+03	2.0398E+03	2.1263E+03	2.0790E+03	2.0245E+03	2.0419E+03	2.0392E+03
	Mean	2.0584E+03	2.1805E+03	2.0723E+03	2.1242E+03	2.1047E+03	2.2215E+03	2.1253E+03	2.0508E+03	2.1269E+03	2.0823E+03
	Std	3.7106E+01	7.5474E+01	1.7476E+01	4.8200E+01	4.6303E+01	5.5592E+01	3.2465E+01	2.6415E+01	5.2377E+01	2.7363E+01
	Rank	2	9	3	6	5	10	7	1	8	4
F20	Best	2.2000E+03	2.2166E+03	2.2046E+03	2.2095E+03	2.2036E+03	2.2293E+03	2.2293E+03	2.2029E+03	2.2060E+03	2.2021E+03
	Mean	2.2599E+03	2.2954E+03	2.2888E+03	2.2306E+03	2.3057E+03	2.2723E+03	2.3147E+03	2.2761E+03	2.2731E+03	2.2373E+03
	Std	5.6292E+01	6.5901E+01	6.7578E+01	2.9129E+01	5.4108E+01	3.5074E+01	3.7874E+01	5.9330E+01	5.3239E+01	5.5293E+01
	Rank	3	8	7	1	9	4	10	6	5	2
F21	Best	2.2458E+03	2.3373E+03	2.2322E+03	2.2790E+03	2.2406E+03	2.3942E+03	2.3081E+03	2.2159E+03	2.2213E+03	2.2270E+03
	Mean	2.2998E+03	2.5360E+03	2.3081E+03	2.3620E+03	2.3198E+03	2.5596E+03	2.3116E+03	2.3071E+03	2.2942E+03	2.3092E+03
	Std	1.0295E+01	1.1180E+02	1.6109E+01	4.6805E+01	1.8852E+01	9.4958E+01	1.5963E+00	2.1336E+01	3.0841E+01	2.7356E+01
	Rank	2	9	4	8	7	10	6	3	1	5
F22	Best	2.6000E+03	2.6426E+03	2.6202E+03	2.6230E+03	2.6197E+03	2.6448E+03	2.6220E+03	2.6083E+03	2.6123E+03	2.6086E+03
	Mean	2.6158E+03	2.6564E+03	2.6396E+03	2.6468E+03	2.6349E+03	2.6678E+03	2.6404E+03	2.6257E+03	2.6334E+03	2.6255E+03
	Std	7.8317E+00	7.1629E+00	1.0625E+01	1.1370E+01	7.4667E+00	8.8128E+00	8.2193E+00	9.4456E+00	1.4341E+01	1.2694E+01
	Rank	1	9	6	8	5	10	7	3	4	2
F23	Best	2.5000E+03	2.6320E+03	2.6766E+03	2.5585E+03	2.5876E+03	2.6998E+03	2.6041E+03	2.5077E+03	2.5261E+03	2.5019E+03
	Mean	2.7218E+03	2.7760E+03	2.7674E+03	2.6774E+03	2.7388E+03	2.7943E+03	2.7536E+03	2.6941E+03	2.7205E+03	2.6938E+03
	Std	6.5603E+01	4.0479E+01	2.0014E+01	8.6776E+01	5.4121E+01	3.0469E+01	3.4798E+01	1.0256E+02	7.4599E+01	1.0682E+02
	Rank	5	9	8	1	6	10	7	3	4	2
F24	Best	2.8977E+03	2.9808E+03	2.8999E+03	2.9138E+03	2.9084E+03	3.0029E+03	2.9279E+03	2.6903E+03	2.6927E+03	2.8979E+03
	Mean	2.9198E+03	3.0424E+03	2.9384E+03	2.9681E+03	2.9430E+03	3.0539E+03	2.9498E+03	2.9275E+03	2.9343E+03	2.9311E+03
	Std	2.3226E+01	3.4495E+01	1.8992E+01	2.7110E+01	1.5842E+01	2.9430E+01	4.6039E+00	5.2702E+01	5.2705E+01	2.8969E+01
	Rank	1	9	5	8	6	10	7	2	4	3
F25	Best	2.9000E+03	3.1038E+03	2.9055E+03	2.9795E+03	2.9384E+03	3.1276E+03	2.9226E+03	2.6476E+03	2.9189E+03	2.8161E+03
	Mean	2.9453E+03	3.2527E+03	2.9494E+03	3.1313E+03	3.0602E+03	3.3129E+03	2.9508E+03	2.9174E+03	3.1100E+03	3.0601E+03
	Std	1.8904E+02	9.7136E+01	2.3581E+01	1.0432E+02	2.1781E+02	8.9291E+01	1.6604E+01	6.7719E+01	2.6801E+02	2.2816E+02
	Rank	2	9	3	8	6	10	4	1	7	5
F26	Best	3.0890E+03	3.1013E+03	3.0927E+03	3.0929E+03	3.0912E+03	3.1023E+03	3.0960E+03	3.0912E+03	3.0919E+03	3.0893E+03
	Mean	3.0954E+03	3.1057E+03	3.0963E+03	3.1004E+03	3.1007E+03	3.1066E+03	3.0992E+03	3.0988E+03	3.0990E+03	3.0967E+03
	Std	5.7849E+00	2.0629E+00	2.2052E+00	3.7684E+00	4.8105E+00	2.4627E+00	1.1894E+00	3.5819E+00	1.6167E+01	5.8043E+00
	Rank	1	9	2	7	8	10	6	4	5	3
F27	Best	3.1000E+03	3.2879E+03	3.1461E+03	3.1804E+03	3.1707E+03	3.2157E+03	3.1837E+03	3.1142E+03	3.1297E+03	3.1008E+03
	Mean	3.2429E+03	3.3809E+03	3.2236E+03	3.3136E+03	3.3212E+03	3.3433E+03	3.2990E+03	3.2584E+03	3.3909E+03	3.2449E+03
	Std	1.2358E+02	5.5347E+01	8.0586E+01	7.4517E+01	1.0716E+02	5.1491E+01	7.2732E+01	1.1415E+02	1.0363E+02	1.1255E+02
	Rank	2	9	1	6	7	8	5	4	10	3
F28	Best	3.1362E+03	3.2088E+03	3.1791E+03	3.1676E+03	3.1642E+03	3.1855E+03	3.2152E+03	3.1631E+03	3.1650E+03	3.1406E+03
	Mean	3.1722E+03	3.2876E+03	3.2410E+03	3.2460E+03	3.2306E+03	3.3209E+03	3.2661E+03	3.2284E+03	3.2696E+03	3.2113E+03
	Std	3.8889E+01	5.5842E+01	5.1718E+01	4.3866E+01	3.8266E+01	5.7499E+01	2.9358E+01	5.2754E+01	7.7216E+01	3.9064E+01
	Rank	1	9	5	6	4	10	7	3	8	2
F29	Best	3.3953E+03	2.1833E+05	5.8507E+03	7.4894E+03	5.2151E+04	1.4900E+05	2.1800E+05	7.1743E+03	6.0525E+03	4.2060E+03
	Mean	1.7467E+06	1.4143E+06	1.4514E+05	1.0731E+06	2.1960E+06	6.4870E+05	1.6362E+06	5.4050E+05	8.1227E+05	9.5531E+05
	Std	2.9411E+06	1.2559E+06	2.5501E+05	9.9532E+05	1.3788E+06	3.5058E+05	1.1142E+06	6.6068E+05	6.5502E+05	1.5022E+06
	Rank	9	7	1	6	10	3	8	2	4	5

**Table A6:** Results obtained by ASFOA and comparison algorithms on CEC 2018 of 30D.

No.	Index	ASFOA	SFOA	LSHADE	MPA	EO	EDO	AE	RIME	ECO	BKA
F1	Best	8.7715E+03	2.3062E+10	4.7634E+07	4.1252E+09	1.3554E+09	1.6481E+10	1.3091E+08	9.3471E+07	3.5473E+08	1.4683E+09
	Mean	1.2953E+08	4.0583E+10	1.2708E+08	1.1233E+10	2.7531E+09	2.2503E+10	2.6662E+08	2.6040E+08	1.7303E+09	5.0925E+09
	Std	1.3463E+08	9.0925E+09	6.0799E+07	3.4779E+09	8.8699E+08	3.4210E+09	7.1925E+07	1.1272E+08	1.1210E+09	2.1263E+09
	Rank	2	10	1	8	6	9	4	3	5	7
F2	Best	3.0008E+02	7.6789E+04	7.0163E+03	4.0238E+04	7.4278E+04	5.9249E+04	5.7525E+04	3.8685E+04	1.4606E+04	9.7222E+03
	Mean	9.2575E+02	1.4961E+05	2.8954E+04	6.2735E+04	1.0779E+05	7.4224E+04	7.6278E+04	7.5195E+04	3.1944E+04	1.8991E+04
	Std	9.1157E+02	3.4071E+04	9.5904E+03	1.3453E+04	2.3204E+04	6.9787E+03	8.8300E+03	2.2067E+04	7.4430E+03	6.0472E+03
	Rank	1	10	3	5	9	6	8	7	4	2
F3	Best	4.7146E+02	3.1490E+03	5.1758E+02	8.5008E+02	5.8909E+02	2.3687E+03	5.3605E+02	5.1353E+02	5.5141E+02	5.4420E+02
	Mean	5.3925E+02	5.6440E+03	5.4948E+02	1.7657E+03	6.9221E+02	3.6281E+03	5.8608E+02	5.8864E+02	6.8042E+02	9.0401E+02
	Std	5.4055E+01	2.0115E+03	1.9374E+01	7.1365E+02	6.4355E+01	6.4948E+02	2.4008E+01	5.6412E+01	1.0192E+02	3.4730E+02
	Rank	1	10	2	8	6	9	3	4	5	7
F4	Best	5.3003E+02	7.5829E+02	6.5464E+02	6.5771E+02	6.4032E+02	7.8392E+02	6.8409E+02	5.9112E+02	6.3274E+02	6.2855E+02
	Mean	5.5436E+02	8.9229E+02	7.0052E+02	7.4764E+02	6.7911E+02	8.3418E+02	7.0776E+02	6.5065E+02	7.1699E+02	7.0978E+02
	Std	1.5198E+01	5.6233E+01	2.0512E+01	3.3158E+01	2.0160E+01	1.8871E+01	1.1043E+01	3.3753E+01	4.2662E+01	3.2529E+01
	Rank	1	10	4	8	3	9	5	2	7	6
F5	Best	6.0058E+02	6.5179E+02	6.0774E+02	6.3222E+02	6.1355E+02	6.5744E+02	6.0643E+02	6.0961E+02	6.2971E+02	6.3696E+02
	Mean	6.0945E+02	6.8059E+02	6.1164E+02	6.4938E+02	6.1932E+02	6.6963E+02	6.0957E+02	6.1826E+02	6.5072E+02	6.5430E+02
	Std	6.7964E+00	1.1313E+01	2.3679E+00	7.8492E+00	3.3825E+00	5.3910E+00	1.8253E+00	5.4477E+00	9.1012E+00	7.7867E+00
	Rank	1	10	3	6	5	9	2	4	7	8
F6	Best	7.7428E+02	1.2703E+03	9.0347E+02	9.9383E+02	9.0237E+02	1.1185E+03	9.1229E+02	8.8173E+02	9.3923E+02	9.5628E+02
	Mean	8.3649E+02	1.4370E+03	9.4388E+02	1.1133E+03	9.6085E+02	1.1949E+03	9.5456E+02	9.6437E+02	1.0887E+03	1.1304E+03
	Std	3.5786E+01	7.5722E+01	1.7322E+01	5.5765E+01	2.9097E+01	3.0863E+01	1.2715E+01	4.7044E+01	6.7802E+01	7.6536E+01
	Rank	1	10	2	7	4	9	3	5	6	8
F7	Best	8.2781E+02	1.0535E+03	9.6822E+02	9.8076E+02	9.0439E+02	1.0779E+03	9.6894E+02	8.9698E+02	8.9664E+02	9.1993E+02
	Mean	8.5352E+02	1.1578E+03	9.9603E+02	1.0265E+03	9.6355E+02	1.1032E+03	9.9643E+02	9.4453E+02	9.8786E+02	9.6314E+02
	Std	1.5237E+01	4.1707E+01	1.7578E+01	2.5768E+01	2.1045E+01	1.5053E+01	1.2079E+01	2.3098E+01	3.7645E+01	2.7641E+01
	Rank	1	10	6	8	4	9	7	2	5	3
F8	Best	9.0151E+02	7.9784E+03	1.0193E+03	3.0432E+03	1.3345E+03	5.7470E+03	1.0471E+03	1.3586E+03	2.6002E+03	3.0761E+03
	Mean	1.0122E+03	1.5035E+04	1.2144E+03	5.3469E+03	2.2067E+03	7.9362E+03	1.1708E+03	3.3501E+03	5.5201E+03	4.5414E+03
	Std	1.2474E+02	3.9334E+03	1.4831E+02	1.1808E+03	6.2973E+02	1.2887E+03	7.7370E+01	1.5178E+03	1.5106E+03	8.3567E+02
	Rank	1	10	3	7	4	9	2	5	8	6
F9	Best	2.6977E+03	6.7987E+03	7.6697E+03	5.1445E+03	6.6528E+03	7.8756E+03	7.9333E+03	4.3922E+03	5.1778E+03	4.0817E+03
	Mean	4.9296E+03	8.0903E+03	8.6438E+03	6.4819E+03	7.8211E+03	8.3400E+03	8.7025E+03	5.5804E+03	6.4085E+03	5.4865E+03
	Std	8.7884E+02	6.0038E+02	4.9769E+02	4.7475E+02	5.3805E+02	2.3998E+02	2.6730E+02	5.5520E+02	6.3103E+02	7.3520E+02
	Rank	1	7	9	5	6	8	10	3	4	2
F10	Best	1.1243E+03	3.9174E+03	1.2905E+03	1.7519E+03	1.5666E+03	3.3283E+03	1.6841E+03	1.3353E+03	1.3378E+03	1.2581E+03
	Mean	1.1579E+03	7.5730E+03	1.3572E+03	2.6247E+03	2.0876E+03	7.7403E+03	2.0353E+03	1.5450E+03	1.4880E+03	1.3874E+03
	Std	2.8265E+01	1.6913E+03	4.2574E+01	6.2647E+02	4.0699E+02	1.9478E+03	2.4536E+02	1.5014E+02	1.0640E+02	1.2229E+02
	Rank	1	9	2	8	7	10	6	5	4	3
F11	Best	1.5079E+03	1.8942E+09	1.5983E+06	1.0699E+08	1.1938E+07	1.5176E+09	6.4756E+06	5.8735E+06	4.7034E+06	4.0197E+06
	Mean	1.5591E+04	4.3124E+09	5.9245E+06	6.1075E+08	5.7661E+07	3.3926E+09	1.5695E+07	3.7796E+07	3.0435E+07	5.2605E+07
	Std	5.9738E+04	1.3274E+09	3.2173E+06	3.8736E+08	3.4582E+07	7.9906E+08	5.4198E+06	2.5483E+07	2.0500E+07	7.6265E+07
	Rank	1	10	2	8	7	9	3	5	4	6
F12	Best	1.4355E+03	5.8001E+08	5.2987E+04	6.0105E+05	2.2233E+05	5.6936E+08	2.4773E+05	3.0020E+05	3.0670E+04	1.8766E+04
	Mean	2.4167E+03	2.2868E+09	2.7088E+05	4.3600E+07	1.9806E+06	1.0343E+09	9.0440E+05	1.0549E+06	1.4986E+05	1.2811E+05
	Std	9.3027E+02	1.0420E+09	2.6406E+05	4.2113E+07	3.0124E+06	3.2544E+08	4.5732E+05	7.3101E+05	9.3361E+04	7.3171E+04
	Rank	1	10	4	8	7	9	5	6	3	2
F13	Best	1.4595E+03	1.6309E+05	1.6312E+03	4.9785E+03	7.5284E+03	1.4434E+05	2.1003E+04	1.1526E+04	1.6565E+03	1.5881E+03
	Mean	1.4741E+03	9.0457E+05	1.8798E+03	8.4342E+04	4.1064E+05	5.4257E+05	7.6860E+04	1.5765E+05	2.3673E+04	3.3040E+03
	Std	1.1964E+01	7.3331E+05	2.8367E+02	8.1854E+04	4.8037E+05	2.4603E+05	3.3466E+04	1.3265E+05	3.1060E+04	6.1722E+03
	Rank	1	10	2	6	8	9	5	7	4	3
F14	Best	1.5469E+03	8.6793E+06	1.6664E+04	5.2249E+04	1.5691E+04	4.6822E+06	2.9302E+04	3.9926E+04	8.5579E+03	3.1428E+03
	Mean	1.6323E+03	1.8412E+08	4.0724E+04	5.0738E+05	7.9097E+04	5.7419E+07	1.0804E+05	1.6567E+05	5.9874E+04	1.8001E+04
	Std	5.7809E+01	1.5659E+08	2.9468E+04	5.7145E+05	7.0213E+04	2.9757E+07	5.6900E+04	8.5154E+04	3.7488E+04	9.9237E+03
	Rank	1	10	3	8	5	9	6	7	4	2
F15	Best	1.7415E+03	3.4950E+03	3.0464E+03	2.6266E+03	2.2736E+03	4.1438E+03	3.2166E+03	2.1958E+03	2.0976E+03	2.5444E+03
	Mean	2.3225E+03	4.1139E+03	3.4835E+03	3.2808E+03	2.9074E+03	4.3751E+03	3.4789E+03	2.7709E+03	3.0223E+03	2.9223E+03
	Std	2.7402E+02	3.2679E+02	1.7656E+02	2.7556E+02	2.4921E+02	1.6277E+02	1.5932E+02	3.4207E+02	4.4513E+02	2.2106E+02
	Rank	1	9	8	6	3	10	7	2	5	4
F16	Best	1.7459E+03	2.2715E+03	2.0326E+03	2.0062E+03	1.8841E+03	2.6767E+03	2.1670E+03	1.8794E+03	1.8589E+03	1.8640E+03
	Mean	1.9583E+03	3.0158E+03	2.3843E+03	2.3609E+03	2.1138E+03	3.0505E+03	2.4117E+03	2.1263E+03	2.2871E+03	2.1861E+03
	Std	1.8630E+02	2.9685E+02	1.4880E+02	2.0825E+02	1.4089E+02	1.9215E+02	1.3777E+02	1.8249E+02	2.2907E+02	1.7028E+02
	Rank	1	9	7	6	2	10	8	3	5	4
F17	Best	1.8336E+03	1.7934E+06	2.2030E+04	1.5908E+05	1.8656E+05	1.0634E+06	4.0356E+05	1.4411E+05	7.2454E+04	1.3299E+04
	Mean	1.8583E+03	1.4446E+07	5.8652E+04	1.1287E+06	1.6071E+06	9.1249E+06	1.0157E+06	2.0303E+06	2.5617E+05	8.6555E+04
	Std	1.8105E+01	1.1927E+07	2.8088E+04	9.6852E+05	1.2520E+06	4.0048E+06	4.1277E+05	1.9136E+06	1.8246E+05	1.0891E+05
	Rank	1	10	2	6	7	9	5	8	4	3
F18	Best	1.9256E+03	2.5945E+07	5.9824E+03	4.6909E+05	1.1891E+04	2.6245E+07	7.3736E+04	3.1133E+04	5.9889E+03	3.0551E+03
	Mean	1.9352E+03	3.4420E+08	4.6613E+04	7.7438E+06	3.1344E+05	8.9209E+07	2.9186E+05	9.5412E+05	5.2069E+05	8.1690E+04
	Std	4.9904E+00	2.6808E+08	4.3379E+04	1.4611E+07	4.3572E+05	3.3106E+07	9.4634E+04	1.2313E+06	8.5195E+05	1.2781E+05
	Rank	1	10	2	8	5	9	4	7	6	3
F19	Best	2.0587E+03	2.5312E+03	2.2864E+03	2.3342E+03	2.3087E+03	2.8167E+03	2.6099E+03	2.1425E+03	2.3431E+03	2.1987E+03
	Mean	2.3943E+03	2.7960E+03	2.7311E+03	2.6269E+03	2.5329E+03	3.0417E+03	2.8252E+03	2.5253E+03	2.6303E+03	2.4499E+03
	Std	2.1490E+02	1.3038E+02	2.1534E+02	1.6318E+02	1.5358E+02	9.9980E+01	1.0209E+02	1.7548E+02	1.3946E+02	1.4576E+02
	Rank	1	8	7	5	4	10	9	3	6	2
F20	Best	2.3271E+03	2.5942E+03	2.4637E+03	2.4473E+03	2.4232E+03	2.5975E+03	2.4844E+03	2.4074E+03	2.4251E+03	2.4282E+03
	Mean	2.3597E+03	2.6481E+03	2.5002E+03	2.5294E+03	2.4589E+03	2.6760E+03	2.5030E+03	2.4399E+03	2.4923E+03	2.5030E+03
	Std	1.5430E+01	3.2907E+01	1.8406E+01	3.5666E+01	2.0402E+01	3.0091E+01	9.5537E+00	2.7948E+01	3.4593E+01	3.6649E+01
	Rank	1	9	5	8	3	10	6	2	4	7
F21	Best	2.3282E+03	4.6053E+03	2.3594E+03	3.2511E+03	2.5319E+03	3.8562E+03	2.3618E+03	2.3529E+03	2.4369E+03	2.5850E+03
	Mean	3.7336E+03	8.4972E+03	2.4021E+03	4.0214E+03	3.7331E+03	6.0530E+03	2.4134E+03	4.5970E+03	4.5986E+03	4.9942E+03
	Std	2.0511E+03	1.6511E+03	2.4354E+01	4.6963E+02	2.3161E+03	8.6725E+02	2.8092E+01	2.4498E+03	2.4917E+03	1.9703E+03
	Rank	4	10	1	5	3	9	2	6	7	8
F22	Best	2.6984E+03	2.9548E+03	2.7711E+03	2.8492E+03	2.7885E+03	3.1321E+03	2.8121E+03	2.7428E+03	2.7735E+03	2.8628E+03
	Mean	2.7355E+03	3.0096E+03	2.8481E+03	2.9444E+03	2.8231E+03	3.2453E+03	2.8516E+03	2.8006E+03	2.8750E+03	2.9751E+03
	Std	2.6554E+01	2.8092E+01	2.6381E+01	4.8611E+01	2.2310E+01	5.5470E+01	1.6741E+01	3.1192E+01	5.4795E+01	7.2153E+01
	Rank	1	9	4	7	3	10	5	2	6	8
F23	Best	2.8563E+03	3.0882E+03	2.9864E+03	3.0398E+03	2.9733E+03	3.2520E+03	2.9763E+03	2.9075E+03	2.9323E+03	2.9492E+03
	Mean	2.9139E+03	3.1340E+03	3.0161E+03	3.1146E+03	2.9943E+03	3.3617E+03	3.0192E+03	2.9555E+03	3.0198E+03	3.0887E+03
	Std	4.2684E+01	2.4826E+01	1.7714E+01	5.4229E+01	1.6144E+01	5.9735E+01	1.5281E+01	2.9073E+01	4.7690E+01	8.3459E+01
	Rank	1	9	4	8	3	10	5	2	6	7
F24	Best	2.8989E+03	3.9130E+03	2.9037E+03	3.0606E+03	2.9979E+03	3.3826E+03	2.9658E+03	2.9306E+03	2.9699E+03	2.9880E+03
	Mean	2.9273E+03	5.0242E+03	2.9358E+03	3.3554E+03	3.0677E+03	3.8257E+03	2.9881E+03	2.9904E+03	3.0609E+03	3.1003E+03
	Std	2.5989E+01	7.1347E+02	1.8197E+01	1.5419E+02	4.0331E+01	1.8104E+02	1.5611E+01	4.4512E+01	4.4607E+01	7.7146E+01
	Rank	1	10	2	8	6	9	3	4	5	7
F25	Best	3.2477E+03	7.1398E+03	4.9768E+03	4.3921E+03	4.4442E+03	7.4373E+03	5.3025E+03	3.6472E+03	3.8260E+03	4.0188E+03
	Mean	4.3298E+03	8.0251E+03	5.5394E+03	6.2016E+03	5.4599E+03	8.0135E+03	5.6022E+03	5.2788E+03	5.8820E+03	6.9642E+03
	Std	3.6463E+02	5.1601E+02	2.4798E+02	8.3204E+02	3.6254E+02	2.5774E+02	1.5466E+02	5.0072E+02	7.9004E+02	9.1347E+02
	Rank	1	10	4	7	3	9	5	2	6	8
F26	Best	3.1906E+03	3.3213E+03	3.2161E+03	3.2413E+03	3.2400E+03	3.3729E+03	3.2521E+03	3.2188E+03	3.2193E+03	3.2583E+03
	Mean	3.2469E+03	3.4372E+03	3.2504E+03	3.3527E+03	3.2730E+03	3.4971E+03	3.2898E+03	3.2503E+03	3.2877E+03	3.3460E+03
	Std	4.4932E+01	6.5346E+01	1.5661E+01	6.8148E+01	1.8501E+01	5.8833E+01	1.3776E+01	1.7915E+01	3.8075E+01	8.1448E+01
	Rank	1	9	3	8	4	10	6	2	5	7
F27	Best	3.2115E+03	4.6022E+03	3.2715E+03	3.4128E+03	3.3789E+03	4.3152E+03	3.3287E+03	3.2629E+03	3.3521E+03	3.3148E+03
	Mean	3.2638E+03	5.7794E+03	3.3094E+03	4.0505E+03	3.5148E+03	4.8707E+03	3.3667E+03	3.3516E+03	3.6465E+03	3.5657E+03
	Std	3.6083E+01	6.2047E+02	2.2924E+01	4.0097E+02	9.0028E+01	3.3793E+02	1.8380E+01	4.1977E+01	4.7048E+02	1.7432E+02
	Rank	1	10	2	8	5	9	4	3	7	6
F28	Best	3.3869E+03	4.6879E+03	3.7776E+03	3.9067E+03	3.7437E+03	4.7593E+03	4.0802E+03	3.5898E+03	3.9710E+03	3.6744E+03
	Mean	3.6442E+03	5.2452E+03	4.2255E+03	4.3597E+03	4.0867E+03	5.2561E+03	4.3474E+03	3.9814E+03	4.4790E+03	4.4584E+03
	Std	1.5722E+02	3.2416E+02	2.3592E+02	2.3329E+02	1.9472E+02	2.1760E+02	1.3048E+02	2.2179E+02	3.5603E+02	4.3570E+02
	Rank	1	9	4	6	3	10	5	2	8	7
F29	Best	5.1136E+03	5.7196E+07	8.9198E+04	2.6184E+06	2.9390E+05	6.1921E+07	1.0355E+06	4.0280E+05	1.1325E+05	1.3765E+05
	Mean	6.4971E+03	2.8815E+08	4.2683E+05	2.5457E+07	4.5906E+06	1.8841E+08	2.2006E+06	3.9761E+06	3.1227E+06	1.1284E+06
	Std	1.4613E+03	1.4218E+08	2.7351E+05	2.0495E+07	3.2471E+06	6.8482E+07	7.0559E+05	3.0782E+06	2.0895E+06	1.0981E+06
	Rank	1	10	2	8	7	9	4	6	5	3

**Table A7:** Results obtained by ASFOA and comparison algorithms on CEC 2018 of 50D.

No.	Index	ASFOA	SFOA	LSHADE	MPA	EO	EDO	AE	RIME	ECO	BKA
F1	Best	1.2014E+09	6.2605E+10	1.0109E+08	1.6375E+10	5.5045E+09	3.6854E+10	7.3581E+08	7.8559E+08	3.0600E+09	1.3430E+10
	Mean	3.6468E+09	8.9943E+10	3.6733E+08	3.1222E+10	1.3096E+10	5.2542E+10	1.2620E+09	1.6917E+09	7.9429E+09	2.1938E+10
	Std	1.9007E+09	1.0922E+10	1.3885E+08	6.4826E+09	3.3999E+09	6.0560E+09	3.0445E+08	5.0777E+08	3.1413E+09	6.9216E+09
	Rank	4	10	1	8	6	9	2	3	5	7
F2	Best	4.1412E+03	1.7917E+05	4.4717E+04	8.3943E+04	1.2230E+05	1.7577E+05	1.2365E+05	1.2918E+05	4.0573E+04	3.0587E+04
	Mean	1.2049E+04	3.0206E+05	7.6437E+04	1.3390E+05	2.3837E+05	2.3932E+05	1.5970E+05	1.8355E+05	7.3366E+04	4.4971E+04
	Std	6.0465E+03	6.1305E+04	1.7061E+04	2.1143E+04	4.7123E+04	4.1180E+04	1.2525E+04	3.0597E+04	1.2689E+04	1.0899E+04
	Rank	1	10	4	5	8	9	6	7	3	2
F3	Best	6.3479E+02	9.7232E+03	6.4540E+02	2.3550E+03	1.0382E+03	6.5344E+03	7.4955E+02	7.2596E+02	1.0398E+03	1.3575E+03
	Mean	8.6764E+02	2.1569E+04	6.9276E+02	4.6147E+03	1.6404E+03	1.0923E+04	8.2100E+02	8.4803E+02	1.4821E+03	2.7339E+03
	Std	1.4890E+02	6.1640E+03	2.5539E+01	1.2016E+03	3.3990E+02	2.3404E+03	3.2547E+01	7.8909E+01	3.5961E+02	1.1655E+03
	Rank	4	10	1	8	6	9	2	3	5	7
F4	Best	5.9620E+02	1.1379E+03	7.8402E+02	8.4257E+02	7.7959E+02	1.0474E+03	8.7604E+02	7.1769E+02	7.4648E+02	7.7489E+02
	Mean	6.4324E+02	1.2332E+03	8.4455E+02	9.7865E+02	8.4521E+02	1.0912E+03	9.1117E+02	8.0625E+02	8.9618E+02	8.5741E+02
	Std	2.9718E+01	6.1630E+01	2.7492E+01	5.3013E+01	3.6486E+01	2.0453E+01	1.5763E+01	4.1520E+01	6.0749E+01	4.7145E+01
	Rank	1	10	3	8	4	9	7	2	6	5
F5	Best	6.0970E+02	6.7198E+02	6.0839E+02	6.5026E+02	6.2075E+02	6.6754E+02	6.1101E+02	6.1781E+02	6.4353E+02	6.5219E+02
	Mean	6.1875E+02	6.9689E+02	6.1234E+02	6.6186E+02	6.2977E+02	6.8265E+02	6.1364E+02	6.3043E+02	6.6265E+02	6.6306E+02
	Std	5.3238E+00	1.1551E+01	2.3676E+00	7.4156E+00	4.3530E+00	5.3900E+00	1.4713E+00	6.7673E+00	9.6293E+00	4.9903E+00
	Rank	3	10	1	6	4	9	2	5	7	8
F6	Best	9.0910E+02	1.8791E+03	1.0642E+03	1.3623E+03	1.1149E+03	1.5399E+03	1.1785E+03	1.1564E+03	1.3665E+03	1.4190E+03
	Mean	1.0026E+03	2.0549E+03	1.1340E+03	1.5199E+03	1.2492E+03	1.6169E+03	1.2019E+03	1.3078E+03	1.5246E+03	1.5846E+03
	Std	6.1592E+01	1.0393E+02	3.0229E+01	7.6339E+01	6.1844E+01	4.3933E+01	1.4766E+01	8.3006E+01	1.0560E+02	9.2711E+01
	Rank	1	10	2	6	4	9	3	5	7	8
F7	Best	9.0567E+02	1.3530E+03	1.0992E+03	1.1666E+03	1.0801E+03	1.3040E+03	1.1700E+03	1.0394E+03	1.1003E+03	1.0385E+03
	Mean	9.4550E+02	1.5359E+03	1.1482E+03	1.2803E+03	1.1579E+03	1.4041E+03	1.2078E+03	1.1106E+03	1.1908E+03	1.1667E+03
	Std	3.0032E+01	8.6487E+01	2.4291E+01	4.9708E+01	3.6242E+01	2.8890E+01	1.9600E+01	3.6703E+01	5.4073E+01	5.6478E+01
	Rank	1	10	3	8	4	9	7	2	6	5
F8	Best	1.1600E+03	2.7063E+04	1.2523E+03	1.0062E+04	5.0836E+03	1.8905E+04	1.6273E+03	4.2595E+03	1.1695E+04	1.0778E+04
	Mean	3.0973E+03	4.4370E+04	1.9947E+03	1.6681E+04	7.9667E+03	2.6381E+04	2.3793E+03	1.1246E+04	1.8458E+04	1.4597E+04
	Std	1.2012E+03	9.1525E+03	3.6855E+02	3.2521E+03	2.1391E+03	3.4604E+03	3.7622E+02	6.2274E+03	3.9512E+03	2.3083E+03
	Rank	3	10	1	7	4	9	2	5	8	6
F9	Best	5.9846E+03	1.2839E+04	1.1684E+04	9.6523E+03	1.0719E+04	1.4485E+04	1.4377E+04	8.6089E+03	8.8893E+03	7.5636E+03
	Mean	8.4901E+03	1.4400E+04	1.4230E+04	1.1184E+04	1.3312E+04	1.5212E+04	1.5177E+04	1.0078E+04	1.1177E+04	9.3341E+03
	Std	1.3167E+03	5.5837E+02	9.5139E+02	7.9068E+02	9.2096E+02	2.5801E+02	3.8160E+02	6.9259E+02	1.1822E+03	1.2549E+03
	Rank	1	8	7	5	6	10	9	3	4	2
F10	Best	1.2022E+03	1.2768E+04	1.4284E+03	4.2125E+03	2.9803E+03	7.8732E+03	3.7645E+03	1.7456E+03	1.7006E+03	1.5910E+03
	Mean	1.3071E+03	1.7665E+04	1.6185E+03	8.1313E+03	5.2044E+03	1.4666E+04	5.3010E+03	2.2892E+03	2.4406E+03	2.8800E+03
	Std	5.9800E+01	3.1293E+03	1.2177E+02	2.0082E+03	1.3683E+03	2.7702E+03	8.6871E+02	3.7664E+02	4.9570E+02	1.2228E+03
	Rank	1	10	2	8	6	9	7	3	4	5
F11	Best	1.1225E+04	1.3864E+10	7.0263E+06	1.4086E+09	1.7630E+08	1.0419E+10	7.1471E+07	1.3088E+08	5.9872E+07	5.9543E+07
	Mean	5.4765E+06	2.7590E+10	3.0715E+07	4.7644E+09	5.3056E+08	2.0539E+10	1.3242E+08	3.9545E+08	3.3126E+08	7.5801E+08
	Std	1.5034E+07	7.5838E+09	1.4872E+07	2.0965E+09	2.0343E+08	4.0819E+09	3.2686E+07	2.1170E+08	2.0308E+08	9.2660E+08
	Rank	1	10	2	8	6	9	3	5	4	7
F12	Best	2.4749E+03	3.2125E+09	1.1246E+05	1.6120E+08	3.7243E+06	3.9471E+09	2.5436E+06	1.5533E+06	1.5673E+05	7.0468E+04
	Mean	5.0669E+03	9.0579E+09	7.2799E+05	8.1521E+08	2.9161E+07	7.9061E+09	5.1427E+06	1.0991E+07	1.2758E+06	4.7856E+06
	Std	1.5213E+03	2.9781E+09	6.0858E+05	4.5205E+08	3.0205E+07	1.6942E+09	1.4578E+06	7.5797E+06	1.1332E+06	1.4498E+07
	Rank	1	10	2	8	7	9	5	6	3	4
F13	Best	1.5367E+03	1.2021E+06	2.4391E+03	1.7546E+05	1.1572E+05	2.2048E+06	1.6922E+05	7.8331E+04	1.2597E+04	2.9079E+03
	Mean	1.5727E+03	5.2809E+06	6.8727E+03	1.3189E+06	1.0975E+06	6.1593E+06	4.3185E+05	6.4309E+05	1.7972E+05	3.1148E+04
	Std	2.7398E+01	2.7323E+06	4.9476E+03	1.0004E+06	9.0725E+05	1.6355E+06	1.7037E+05	5.3499E+05	2.4020E+05	2.5987E+04
	Rank	1	9	2	8	7	10	5	6	4	3
F14	Best	1.7308E+03	8.6568E+08	2.4740E+04	3.1064E+06	7.4682E+04	4.3289E+08	2.0688E+05	1.7207E+05	1.9500E+04	1.1004E+04
	Mean	1.9395E+03	2.8958E+09	8.0268E+04	4.4085E+07	7.0416E+05	1.1468E+09	5.1057E+05	1.1859E+06	5.9783E+04	5.1244E+04
	Std	1.5412E+02	1.2947E+09	6.7360E+04	4.3044E+07	6.3534E+05	2.8664E+08	1.7788E+05	9.8585E+05	8.1154E+04	3.1846E+04
	Rank	1	10	4	8	6	9	5	7	3	2
F15	Best	2.0975E+03	5.5715E+03	3.7554E+03	3.6338E+03	3.0687E+03	5.3328E+03	4.1085E+03	2.8153E+03	2.8432E+03	3.5656E+03
	Mean	2.7883E+03	6.5505E+03	4.7561E+03	4.5307E+03	3.8556E+03	6.5602E+03	4.6941E+03	3.6045E+03	3.9033E+03	4.1299E+03
	Std	3.4178E+02	4.1820E+02	4.1696E+02	5.0977E+02	3.7264E+02	4.5176E+02	2.2903E+02	3.7027E+02	3.9192E+02	3.4206E+02
	Rank	1	9	8	6	3	10	7	2	4	5
F16	Best	2.1025E+03	4.3054E+03	3.1667E+03	3.1204E+03	2.7349E+03	4.5154E+03	3.4835E+03	2.8642E+03	2.9246E+03	2.9733E+03
	Mean	2.5931E+03	6.2455E+03	3.8540E+03	3.7491E+03	3.3530E+03	5.0692E+03	3.9271E+03	3.3806E+03	3.4660E+03	3.4085E+03
	Std	2.7915E+02	1.2959E+03	2.8583E+02	3.2309E+02	3.7475E+02	2.5022E+02	1.7306E+02	3.3067E+02	2.8537E+02	3.0452E+02
	Rank	1	10	7	6	2	9	8	3	5	4
F17	Best	1.9165E+03	9.0326E+06	4.5579E+04	7.8889E+05	1.0219E+06	9.0631E+06	1.3098E+06	2.6457E+05	1.9961E+05	7.8683E+04
	Mean	2.0246E+03	4.9978E+07	1.1204E+05	6.6161E+06	6.7710E+06	2.9733E+07	3.3860E+06	5.4604E+06	1.7714E+06	3.8506E+05
	Std	9.1449E+01	2.5767E+07	4.8492E+04	4.7747E+06	6.2883E+06	1.1067E+07	1.2434E+06	4.2543E+06	1.7326E+06	2.9781E+05
	Rank	1	10	2	7	8	9	5	6	4	3
F18	Best	1.9660E+03	3.2114E+08	1.4275E+04	1.8317E+06	9.8373E+04	9.3346E+07	1.8281E+05	8.4991E+04	1.4868E+04	6.2370E+04
	Mean	1.9973E+03	1.3203E+09	1.1193E+05	3.5342E+07	7.9579E+05	6.5569E+08	3.4533E+05	2.9482E+06	1.7692E+05	5.0970E+05
	Std	2.3899E+01	6.5718E+08	7.4344E+04	2.7529E+07	6.0452E+05	2.1372E+08	1.0095E+05	2.4548E+06	1.7371E+05	7.5040E+05
	Rank	1	10	2	8	6	9	4	7	3	5
F19	Best	2.3302E+03	3.5041E+03	3.3375E+03	2.9652E+03	2.7847E+03	3.4057E+03	3.7205E+03	2.4418E+03	2.8407E+03	2.5178E+03
	Mean	2.8724E+03	3.9761E+03	3.8004E+03	3.3978E+03	3.3845E+03	4.0474E+03	4.0335E+03	3.3119E+03	3.3018E+03	3.0549E+03
	Std	3.0268E+02	2.9686E+02	2.7878E+02	2.0897E+02	2.8948E+02	2.0792E+02	1.5223E+02	2.9544E+02	2.3682E+02	2.4245E+02
	Rank	1	8	7	6	5	10	9	4	3	2
F20	Best	2.4013E+03	2.8959E+03	2.6080E+03	2.6993E+03	2.5684E+03	2.9627E+03	2.6257E+03	2.5204E+03	2.5622E+03	2.6235E+03
	Mean	2.4473E+03	3.0457E+03	2.6508E+03	2.7659E+03	2.6329E+03	3.1360E+03	2.6833E+03	2.6069E+03	2.6932E+03	2.7566E+03
	Std	2.8049E+01	7.2876E+01	1.9554E+01	4.1346E+01	3.4899E+01	6.1987E+01	2.2327E+01	4.4654E+01	6.1885E+01	6.9395E+01
	Rank	1	9	4	8	3	10	5	2	6	7
F21	Best	7.1652E+03	1.4456E+04	3.3037E+03	8.0378E+03	5.0266E+03	1.5949E+04	1.2426E+04	9.8438E+03	4.3196E+03	9.1393E+03
	Mean	1.0052E+04	1.5929E+04	1.5540E+04	1.2449E+04	1.4578E+04	1.6596E+04	1.6400E+04	1.1455E+04	1.2351E+04	1.0845E+04
	Std	1.2379E+03	6.2138E+02	2.4726E+03	1.7655E+03	1.9325E+03	2.6989E+02	9.0171E+02	8.6942E+02	1.9877E+03	8.1893E+02
	Rank	1	8	7	5	6	10	9	3	4	2
F22	Best	2.8319E+03	3.3763E+03	2.9990E+03	3.1508E+03	3.0308E+03	3.5935E+03	3.0636E+03	2.9660E+03	3.1026E+03	3.2732E+03
	Mean	2.9610E+03	3.5202E+03	3.0885E+03	3.3928E+03	3.0963E+03	3.8342E+03	3.1400E+03	3.0782E+03	3.2513E+03	3.4653E+03
	Std	6.1996E+01	6.7160E+01	3.6494E+01	1.0322E+02	3.1806E+01	1.2037E+02	2.0991E+01	5.4797E+01	1.1131E+02	1.1657E+02
	Rank	1	9	3	7	4	10	5	2	6	8
F23	Best	3.0360E+03	3.5232E+03	3.1925E+03	3.3380E+03	3.1767E+03	3.9492E+03	3.2109E+03	3.1259E+03	3.2442E+03	3.4176E+03
	Mean	3.1612E+03	3.5827E+03	3.2523E+03	3.5735E+03	3.2461E+03	4.1531E+03	3.2922E+03	3.2066E+03	3.4144E+03	3.5983E+03
	Std	6.9973E+01	3.8643E+01	2.4800E+01	1.1765E+02	3.3718E+01	9.7301E+01	2.8774E+01	4.7618E+01	9.4350E+01	1.3389E+02
	Rank	1	8	4	7	3	10	5	2	6	9
F24	Best	3.0845E+03	9.9035E+03	3.0695E+03	4.3611E+03	3.7119E+03	6.7612E+03	3.2465E+03	3.1165E+03	3.4759E+03	3.3144E+03
	Mean	3.2972E+03	1.3998E+04	3.1446E+03	5.9064E+03	4.1378E+03	8.2197E+03	3.3391E+03	3.3282E+03	3.9647E+03	4.7963E+03
	Std	1.1791E+02	1.8987E+03	3.2689E+01	8.5262E+02	2.4045E+02	8.5175E+02	5.4971E+01	1.2127E+02	4.3846E+02	6.9242E+02
	Rank	2	10	1	8	6	9	4	3	5	7
F25	Best	4.9980E+03	1.1218E+04	6.5442E+03	7.1291E+03	6.1461E+03	8.8808E+03	7.2046E+03	6.3806E+03	5.8713E+03	5.1713E+03
	Mean	6.1490E+03	1.2648E+04	7.1024E+03	9.6973E+03	7.4591E+03	1.1311E+04	7.8435E+03	7.3157E+03	8.9920E+03	1.1111E+04
	Std	6.5485E+02	7.0377E+02	2.9141E+02	1.0929E+03	5.1689E+02	8.1773E+02	3.0472E+02	4.9337E+02	1.5598E+03	2.0526E+03
	Rank	1	10	2	7	4	9	5	3	6	8
F26	Best	3.2767E+03	4.0294E+03	3.3369E+03	3.6111E+03	3.5091E+03	4.0887E+03	3.5356E+03	3.4194E+03	3.5533E+03	3.6086E+03
	Mean	3.6322E+03	4.3115E+03	3.4483E+03	3.9396E+03	3.7035E+03	4.3580E+03	3.7531E+03	3.5895E+03	3.7878E+03	3.9597E+03
	Std	2.0819E+02	1.6948E+02	5.4801E+01	1.4122E+02	8.1110E+01	1.2251E+02	1.3423E+02	7.8526E+01	1.7485E+02	2.1049E+02
	Rank	3	9	1	7	4	10	5	2	6	8
F27	Best	3.3601E+03	8.6529E+03	3.3904E+03	5.0542E+03	4.4399E+03	7.8669E+03	3.6204E+03	3.5817E+03	3.8597E+03	4.1803E+03
	Mean	3.6330E+03	1.0964E+04	3.4564E+03	6.3410E+03	4.9428E+03	8.8914E+03	3.8425E+03	3.9951E+03	4.7247E+03	5.2426E+03
	Std	2.6263E+02	1.3244E+03	4.4240E+01	6.9317E+02	3.1678E+02	4.9662E+02	1.0682E+02	3.4015E+02	5.9114E+02	6.2180E+02
	Rank	2	10	1	8	6	9	3	4	5	7
F28	Best	3.5158E+03	7.9662E+03	4.5081E+03	5.2807E+03	4.5099E+03	7.4744E+03	5.0723E+03	4.1340E+03	4.6888E+03	4.7909E+03
	Mean	3.9012E+03	1.0372E+04	5.1142E+03	6.1673E+03	5.0455E+03	9.0991E+03	5.5709E+03	4.8693E+03	5.6659E+03	6.2555E+03
	Std	2.4946E+02	1.3260E+03	3.2453E+02	4.2309E+02	3.3965E+02	6.6017E+02	2.0880E+02	3.5640E+02	5.4666E+02	1.0085E+03
	Rank	1	10	4	7	3	9	5	2	6	8
F29	Best	9.2487E+05	8.4153E+08	1.4095E+07	8.5880E+07	4.3362E+07	6.4487E+08	2.6791E+07	4.3569E+07	1.2617E+07	1.4728E+07
	Mean	2.0087E+06	2.0408E+09	1.7906E+07	2.5536E+08	9.3774E+07	1.3899E+09	4.1665E+07	9.8391E+07	3.9031E+07	5.8144E+07
	Std	6.1840E+05	7.5488E+08	3.4218E+06	1.4260E+08	3.6600E+07	3.6389E+08	8.0977E+06	3.5126E+07	1.4809E+07	3.4327E+07
	Rank	1	10	2	8	6	9	4	7	3	5

**Table A8:** Results obtained by ASFOA and comparison algorithms on CEC 2018 of 100D.

No.	Index	ASFOA	SFOA	LSHADE	MPA	EO	EDO	AE	RIME	ECO	BKA
F1	Best	1.8656E+10	1.9678E+11	9.6513E+08	8.4722E+10	4.4775E+10	1.3661E+11	5.2402E+09	7.9819E+09	2.7387E+10	6.5239E+10
	Mean	3.1238E+10	2.2991E+11	1.4885E+09	1.1369E+11	6.3049E+10	1.5795E+11	6.6169E+09	1.2293E+10	4.4752E+10	8.8715E+10
	Std	7.4148E+09	1.6906E+10	3.0092E+08	1.5945E+10	7.3940E+09	1.2607E+10	1.0370E+09	2.5829E+09	8.5622E+09	1.3509E+10
	Rank	4	10	1	8	6	9	2	3	5	7
F2	Best	4.6400E+04	4.9257E+05	1.5097E+05	2.4507E+05	4.4705E+05	3.3671E+05	3.0675E+05	3.9345E+05	1.5995E+05	1.1710E+05
	Mean	7.0330E+04	7.4518E+05	1.9803E+05	3.0218E+05	5.6519E+05	3.7383E+05	3.4177E+05	5.5152E+05	2.0681E+05	1.5630E+05
	Std	1.2969E+04	1.0978E+05	2.2427E+04	2.9169E+04	5.8220E+04	2.7538E+04	1.8667E+04	8.3151E+04	2.2322E+04	2.2005E+04
	Rank	1	10	3	5	9	7	6	8	4	2
F3	Best	2.1365E+03	4.0945E+04	9.2026E+02	8.3641E+03	3.7678E+03	3.0940E+04	1.3314E+03	1.4159E+03	2.9506E+03	6.1069E+03
	Mean	3.3471E+03	7.0085E+04	1.0327E+03	1.7036E+04	6.7686E+03	4.1522E+04	1.5782E+03	2.0270E+03	4.7924E+03	1.2352E+04
	Std	9.3507E+02	1.2625E+04	6.1080E+01	4.3967E+03	1.5347E+03	4.9202E+03	1.2141E+02	3.7192E+02	1.3506E+03	5.1296E+03
	Rank	4	10	1	8	6	9	2	3	5	7
F4	Best	8.9943E+02	1.9988E+03	1.1414E+03	1.4455E+03	1.2741E+03	1.8276E+03	1.3678E+03	1.2234E+03	1.4030E+03	1.2595E+03
	Mean	9.9622E+02	2.1487E+03	1.2607E+03	1.6645E+03	1.4182E+03	1.9030E+03	1.4703E+03	1.3648E+03	1.5269E+03	1.4291E+03
	Std	5.9191E+01	1.0326E+02	4.2284E+01	9.9918E+01	4.9834E+01	4.2334E+01	4.3810E+01	9.3240E+01	8.5680E+01	8.8556E+01
	Rank	1	10	2	8	4	9	6	3	7	5
F5	Best	6.2464E+02	7.0476E+02	6.1244E+02	6.6895E+02	6.3926E+02	6.7931E+02	6.1792E+02	6.3811E+02	6.6441E+02	6.6254E+02
	Mean	6.3347E+02	7.2200E+02	6.1717E+02	6.7753E+02	6.4845E+02	6.9414E+02	6.2122E+02	6.5051E+02	6.7565E+02	6.7354E+02
	Std	4.9829E+00	1.0532E+01	2.1293E+00	4.4850E+00	4.9726E+00	5.8557E+00	1.4776E+00	6.9697E+00	5.3017E+00	5.1927E+00
	Rank	3	10	1	8	4	9	2	5	7	6
F6	Best	1.4159E+03	3.5922E+03	1.5479E+03	2.6940E+03	2.0558E+03	2.8689E+03	1.7300E+03	2.0310E+03	2.5705E+03	2.7669E+03
	Mean	1.7219E+03	3.9403E+03	1.6309E+03	2.9736E+03	2.3020E+03	3.1118E+03	1.8096E+03	2.4304E+03	3.0149E+03	3.0782E+03
	Std	1.3583E+02	1.5200E+02	4.2428E+01	1.7422E+02	1.2965E+02	1.1562E+02	4.1309E+01	2.6314E+02	2.6506E+02	1.2333E+02
	Rank	2	10	1	6	4	9	3	5	7	8
F7	Best	1.1970E+03	2.4347E+03	1.4825E+03	1.8838E+03	1.5507E+03	2.1566E+03	1.7261E+03	1.4747E+03	1.6689E+03	1.7071E+03
	Mean	1.3092E+03	2.5900E+03	1.5659E+03	2.0224E+03	1.7126E+03	2.2451E+03	1.7830E+03	1.6541E+03	1.8958E+03	1.8765E+03
	Std	5.7236E+01	1.0929E+02	3.5398E+01	8.0491E+01	7.2376E+01	4.6767E+01	2.4909E+01	7.3449E+01	1.1064E+02	1.0482E+02
	Rank	1	10	2	8	4	9	5	3	7	6
F8	Best	7.9993E+03	6.8194E+04	2.9067E+03	3.4004E+04	2.1613E+04	5.8454E+04	5.6457E+03	2.2683E+04	3.4351E+04	2.7917E+04
	Mean	1.6693E+04	1.1544E+05	4.6175E+03	4.3687E+04	3.3899E+04	6.7062E+04	8.8951E+03	4.1461E+04	4.2025E+04	3.3294E+04
	Std	5.0865E+03	2.9601E+04	1.0306E+03	4.8883E+03	5.6942E+03	5.1591E+03	2.0103E+03	1.2505E+04	4.6871E+03	4.8954E+03
	Rank	3	10	1	8	5	9	2	6	7	4
F9	Best	1.4099E+04	2.9316E+04	2.7872E+04	2.2573E+04	2.6196E+04	2.9854E+04	3.1041E+04	2.1327E+04	1.9326E+04	1.5062E+04
	Mean	1.8575E+04	3.1001E+04	3.0135E+04	2.4434E+04	2.8690E+04	3.1552E+04	3.2039E+04	2.3419E+04	2.3107E+04	1.9888E+04
	Std	2.2514E+03	8.0885E+02	1.2330E+03	9.4754E+02	1.1670E+03	6.0641E+02	4.9104E+02	1.2640E+03	2.2560E+03	2.2268E+03
	Rank	1	8	7	5	6	9	10	4	3	2
F10	Best	2.0433E+03	1.1902E+05	6.8318E+03	6.6685E+04	5.9133E+04	1.6517E+05	6.2536E+04	1.7560E+04	1.1484E+04	7.2907E+03
	Mean	2.7581E+03	2.0638E+05	1.0697E+04	9.9749E+04	9.1332E+04	2.3017E+05	8.7957E+04	4.3447E+04	2.8726E+04	1.5504E+04
	Std	3.0281E+02	4.6062E+04	3.2308E+03	1.8888E+04	1.6116E+04	3.9416E+04	1.2759E+04	1.4253E+04	1.1981E+04	6.0420E+03
	Rank	1	9	2	8	7	10	6	5	4	3
F11	Best	3.4767E+07	9.1249E+10	1.2250E+08	1.3899E+10	2.8179E+09	5.2136E+10	6.6385E+08	1.0473E+09	1.4613E+09	3.0392E+09
	Mean	8.7817E+08	1.2639E+11	2.3105E+08	2.5658E+10	5.2487E+09	7.1770E+10	8.7771E+08	2.1347E+09	2.6329E+09	1.2626E+10
	Std	7.0848E+08	1.9121E+10	6.4500E+07	6.1254E+09	1.7159E+09	8.8450E+09	1.1674E+08	7.1533E+08	6.7168E+08	9.6605E+09
	Rank	3	10	1	8	6	9	2	4	5	7
F12	Best	7.1107E+03	1.1654E+10	7.5002E+04	7.4891E+08	3.1842E+07	9.2340E+09	4.2546E+06	8.7734E+06	3.2770E+06	9.7022E+06
	Mean	1.3052E+04	2.4452E+10	2.9473E+05	2.5411E+09	1.2893E+08	1.2790E+10	7.7517E+06	3.4735E+07	1.9097E+07	4.6343E+08
	Std	3.9578E+03	6.4288E+09	1.5527E+05	1.1094E+09	5.9240E+07	1.5203E+09	1.5966E+06	1.4631E+07	1.6993E+07	5.9718E+08
	Rank	1	10	2	8	6	9	3	5	4	7
F13	Best	1.7604E+03	2.1113E+07	3.6469E+04	2.5680E+06	3.2177E+06	1.3802E+07	1.1819E+06	2.1453E+06	5.1634E+05	1.7029E+05
	Mean	1.8259E+03	6.1706E+07	1.0128E+05	9.2574E+06	7.4363E+06	2.6379E+07	3.8806E+06	7.2620E+06	1.2676E+06	8.4549E+05
	Std	3.9498E+01	2.4854E+07	4.4655E+04	3.9710E+06	2.6935E+06	6.9325E+06	1.1509E+06	4.1795E+06	5.5340E+05	5.1747E+05
	Rank	1	10	2	8	7	9	5	6	4	3
F14	Best	2.0140E+03	3.4866E+09	2.1536E+04	9.6798E+07	9.8695E+05	2.8063E+09	3.3852E+05	1.4688E+06	4.8884E+04	4.4999E+04
	Mean	2.4686E+03	8.6943E+09	5.2097E+04	4.6807E+08	4.9403E+06	4.8621E+09	7.4943E+05	5.0894E+06	7.0108E+05	1.0046E+07
	Std	3.3476E+02	2.3791E+09	1.8536E+04	2.7678E+08	2.1780E+06	8.2969E+08	1.9593E+05	2.6082E+06	1.1585E+06	3.6140E+07
	Rank	1	10	2	8	5	9	4	6	3	7
F15	Best	3.1097E+03	1.2340E+04	7.3038E+03	7.7732E+03	6.0929E+03	1.2245E+04	8.7706E+03	6.0078E+03	6.5677E+03	6.2050E+03
	Mean	4.8073E+03	1.4401E+04	8.9554E+03	9.5902E+03	7.5700E+03	1.3922E+04	9.7484E+03	7.1552E+03	7.7997E+03	8.5697E+03
	Std	8.3085E+02	1.3565E+03	5.0298E+02	6.4368E+02	6.5673E+02	5.1106E+02	4.1910E+02	7.1878E+02	7.7908E+02	1.4715E+03
	Rank	1	10	6	7	3	9	8	2	4	5
F16	Best	2.9288E+03	1.4705E+04	4.8112E+03	6.1194E+03	4.1039E+03	1.5080E+04	6.1757E+03	3.9820E+03	4.8552E+03	4.8044E+03
	Mean	4.0847E+03	7.4825E+04	6.4100E+03	7.7759E+03	5.6400E+03	4.8702E+04	6.9354E+03	5.7319E+03	5.8655E+03	6.2357E+03
	Std	4.7091E+02	6.4345E+04	5.5357E+02	1.0487E+03	5.3147E+02	2.9094E+04	2.6789E+02	6.2510E+02	6.5278E+02	8.9278E+02
	Rank	1	10	6	8	2	9	7	3	4	5
F17	Best	2.1844E+03	2.7518E+07	1.4544E+05	2.1676E+06	1.9515E+06	2.3269E+07	2.5023E+06	2.6055E+06	6.7299E+05	2.9731E+05
	Mean	2.3936E+03	9.6594E+07	2.5598E+05	1.3680E+07	7.9452E+06	5.4345E+07	3.7403E+06	1.0792E+07	2.3636E+06	1.1921E+06
	Std	1.8990E+02	4.1099E+07	1.0009E+05	6.9233E+06	3.9316E+06	1.3682E+07	5.9750E+05	5.4065E+06	1.5553E+06	9.3397E+05
	Rank	1	10	2	8	6	9	5	7	4	3
F18	Best	2.0863E+03	3.8417E+09	1.1447E+05	7.0503E+07	5.1722E+06	3.6403E+09	1.2781E+06	6.6541E+06	2.7503E+05	1.7599E+06
	Mean	2.2280E+03	9.3343E+09	3.4029E+05	5.0334E+08	1.3453E+07	5.0432E+09	2.2976E+06	2.1652E+07	2.9014E+06	2.7822E+07
	Std	9.4775E+01	3.1914E+09	2.1925E+05	2.3432E+08	6.0811E+06	8.1335E+08	5.5177E+05	1.2162E+07	2.0425E+06	7.6519E+07
	Rank	1	10	2	8	5	9	3	6	4	7
F19	Best	3.0980E+03	6.2739E+03	6.2193E+03	5.1026E+03	4.7703E+03	6.8285E+03	6.8509E+03	4.5485E+03	4.2392E+03	4.2777E+03
	Mean	4.5095E+03	7.3320E+03	7.0338E+03	5.9680E+03	5.7770E+03	7.5911E+03	7.3590E+03	5.7310E+03	5.6970E+03	5.1815E+03
	Std	5.5392E+02	4.6963E+02	3.3952E+02	4.2162E+02	5.3379E+02	2.7635E+02	2.2496E+02	4.8523E+02	5.7138E+02	4.5014E+02
	Rank	1	8	7	6	5	10	9	4	3	2
F20	Best	2.7606E+03	4.0296E+03	2.9631E+03	3.3939E+03	3.0679E+03	3.9664E+03	3.2060E+03	3.0855E+03	3.2040E+03	3.2653E+03
	Mean	2.8846E+03	4.2323E+03	3.1118E+03	3.5955E+03	3.1867E+03	4.3150E+03	3.2546E+03	3.2225E+03	3.4376E+03	3.6609E+03
	Std	7.1527E+01	1.2648E+02	5.0295E+01	1.0263E+02	5.0887E+01	1.4192E+02	2.6008E+01	7.3860E+01	1.2410E+02	1.9580E+02
	Rank	1	9	2	7	3	10	5	4	6	8
F21	Best	1.6813E+04	3.1092E+04	2.9991E+04	2.3050E+04	2.8101E+04	3.2761E+04	3.1842E+04	2.2367E+04	2.1735E+04	1.9290E+04
	Mean	2.0884E+04	3.2953E+04	3.2579E+04	2.7185E+04	3.0687E+04	3.4401E+04	3.4145E+04	2.5728E+04	2.6104E+04	2.2929E+04
	Std	2.1981E+03	7.4832E+02	1.3731E+03	1.2705E+03	1.2272E+03	5.5389E+02	6.9853E+02	1.6351E+03	1.6331E+03	2.6224E+03
	Rank	1	8	7	5	6	10	9	3	4	2
F22	Best	3.5001E+03	4.3098E+03	3.5823E+03	4.2166E+03	3.6064E+03	4.8468E+03	3.7023E+03	3.4889E+03	3.8529E+03	3.9977E+03
	Mean	3.7487E+03	4.5918E+03	3.6931E+03	4.4822E+03	3.7172E+03	5.4692E+03	3.8115E+03	3.6906E+03	4.1143E+03	4.5115E+03
	Std	1.6862E+02	1.0128E+02	6.2679E+01	1.6729E+02	5.5624E+01	2.2720E+02	4.6128E+01	8.3674E+01	1.3131E+02	2.3271E+02
	Rank	4	9	2	7	3	10	5	1	6	8
F23	Best	4.1806E+03	5.2053E+03	4.0056E+03	4.9379E+03	4.1498E+03	6.5504E+03	4.1355E+03	4.0733E+03	4.3556E+03	5.1642E+03
	Mean	4.6037E+03	5.5709E+03	4.1354E+03	5.6156E+03	4.2843E+03	7.5927E+03	4.2310E+03	4.2822E+03	4.9000E+03	5.8106E+03
	Std	2.6655E+02	1.6942E+02	5.9494E+01	2.7501E+02	8.1505E+01	5.0742E+02	4.6696E+01	1.0402E+02	2.2708E+02	4.7483E+02
	Rank	5	7	1	8	4	10	2	3	6	9
F24	Best	4.2124E+03	1.9394E+04	3.5903E+03	9.3271E+03	6.5777E+03	1.5312E+04	4.0796E+03	4.4186E+03	5.2481E+03	6.5143E+03
	Mean	5.0318E+03	2.5889E+04	3.7527E+03	1.1272E+04	7.6066E+03	1.6913E+04	4.4261E+03	5.0175E+03	6.4963E+03	8.8007E+03
	Std	4.7829E+02	3.2108E+03	7.6594E+01	1.3224E+03	5.7610E+02	9.2180E+02	1.3729E+02	3.4701E+02	7.0001E+02	1.2814E+03
	Rank	4	10	1	8	6	9	2	3	5	7
F25	Best	1.1921E+04	2.6709E+04	1.2830E+04	2.0338E+04	1.5195E+04	2.5416E+04	1.4654E+04	1.3650E+04	1.4390E+04	2.4303E+04
	Mean	1.6267E+04	3.0351E+04	1.3757E+04	2.4057E+04	1.7190E+04	2.7828E+04	1.5403E+04	1.6347E+04	2.1666E+04	3.0722E+04
	Std	2.4070E+03	1.9096E+03	5.3141E+02	2.3056E+03	1.3848E+03	1.5768E+03	4.5395E+02	1.1047E+03	2.9462E+03	3.5121E+03
	Rank	3	9	1	7	5	8	2	4	6	10
F26	Best	3.4635E+03	5.4857E+03	3.4969E+03	4.5231E+03	3.9213E+03	6.6897E+03	3.7268E+03	3.7523E+03	3.7636E+03	3.8855E+03
	Mean	3.6228E+03	6.2147E+03	3.6079E+03	5.0624E+03	4.1649E+03	7.6503E+03	3.8127E+03	3.9828E+03	4.0844E+03	4.6474E+03
	Std	1.5747E+02	4.9885E+02	6.3057E+01	2.8106E+02	1.4614E+02	3.1229E+02	6.1932E+01	1.5328E+02	1.8184E+02	4.0084E+02
	Rank	2	9	1	8	6	10	3	4	5	7
F27	Best	4.2261E+03	2.2163E+04	3.7978E+03	1.2833E+04	7.7251E+03	1.6912E+04	4.8490E+03	5.0573E+03	6.7135E+03	9.0964E+03
	Mean	5.9712E+03	3.1044E+04	4.0033E+03	1.5590E+04	1.0828E+04	1.9245E+04	5.3386E+03	7.5684E+03	8.6306E+03	1.2786E+04
	Std	8.6085E+02	3.5389E+03	1.5409E+02	2.0366E+03	1.3222E+03	1.1243E+03	3.1829E+02	1.4727E+03	2.3100E+03	2.1044E+03
	Rank	3	10	1	8	6	9	2	4	5	7
F28	Best	4.8569E+03	3.5826E+04	7.8629E+03	1.0075E+04	7.3281E+03	2.0323E+04	9.3521E+03	7.5146E+03	8.3651E+03	8.6699E+03
	Mean	6.2462E+03	6.9377E+04	8.7763E+03	1.2564E+04	8.7321E+03	3.4855E+04	9.9959E+03	9.2059E+03	9.6934E+03	1.1683E+04
	Std	6.8596E+02	2.6102E+04	3.5263E+02	1.6098E+03	6.1214E+02	6.9776E+03	2.9787E+02	7.5759E+02	8.5577E+02	2.2804E+03
	Rank	1	10	3	8	2	9	6	4	5	7
F29	Best	2.2672E+04	6.3811E+09	2.5512E+06	5.8605E+08	5.5275E+07	6.5489E+09	2.2376E+07	5.2031E+07	1.7421E+07	3.9103E+07
	Mean	3.2283E+05	1.4101E+10	4.8480E+06	1.7944E+09	1.7910E+08	8.7634E+09	3.3871E+07	2.3229E+08	7.3390E+07	3.3450E+08
	Std	4.6528E+05	3.2728E+09	1.6066E+06	6.7454E+08	7.0119E+07	1.2582E+09	7.4106E+06	1.0530E+08	3.8716E+07	5.4069E+08
	Rank	1	10	2	8	5	9	3	6	4	7
